# Supplementary material for: Consistency of causal claims in observational studies: a review of papers published in a general medical journal
Source: BMJ Open. 2021 May 20;11(5):e043339. doi: 10.1136/bmjopen-2020-043339 (PMC8141434; doi:10.1136/bmjopen-2020-043339)
Supplement: Supplementary data [file bmjopen-2020-043339supp001.pdf]

Supplementary Material

Table A. Statements in each of the included observational studies published in the BMJ in 2018

Published Abstract		Published Full text				Comment
Objective	Conclusion	Method	Confounder adjustment	Estimates provided	Authors Considerations	
CONSISTENTLY CAUSAL						
"To evaluate the **impact** of […] on […] in […]"	"[…] is associated with negative **effects** on […]. Given the relatively low prevalence of […], population level impacts are currently modest. Nevertheless, as […] has doubled in the US over the past generation, further investigation is warranted of the **impact** on	"To estimate the **adjusted** odds ratio for each […] outcome by […] group, we created logistic regression models with […] as the reference group. […] The **population attributable risk** was calculated using the standard formula"	"Other subgroup analyses were done to ensure that […] association was **not confounded by** […]"	"[…] had 14% higher odds of […] compared with […] (adjusted odds ratio 1.14, 99% confidence interval 1.13 to 1.15). […] 14.5% (13.6% to 15.4%) of […] (under the assumption of a **causal** relation) can be **attributed to** […]"	"The pooling of all […] during this period minimizes the risk of **confounding** from yearly fluctuations in […] outcomes. Finally, despite attempts to adjust […] using regression analysis and stratification, some **residual confounding** effects from […] could remain. […] As more than 12% of […] **might have been**	Both abstract and main text use causal language: they state that their aim is to assess the impact of the exposure on the outcome, adjust for confounders, provide population attributable risks and discuss residual confounding.

	[…] and public health.				**prevented** were […], the importance of these data are most relevant to […]. The cumulative risk over […] is also likely to be **important in terms of both economic burden and overall public health**”	
“To examine the […] **risks of […] initiation** compared with initiation of other traditional […] drugs, initiation of […], and no initiation.”	“[…] **poses a […] risk** compared with non-use, […] use, and use of other traditional […] drugs.”	“[W]e conducted a series of cohort studies, each mimicking the strict design criteria of a clinical trial (a so-called **emulated trial design**), to compare rates of […] among […] with rates among […]. […] We estimated an observational analogue of the **intention to treat** hazard ratio, as a measure of the incidence rate ratio,	“We calculated the **propensity score** for all eligible individuals initiating […] at enrolment by fitting a logistic regression model including covariates on sex, age, year, comorbidity, and	“[…] initiators had a 50% **increased rate of** […] events compared with […] non-initiators (incidence rate ratio 1.5, 95% confidence interval 1.4 to 1.7).”	“We performed the following sensitivity analyses, […] to estimate how strongly a single **unmeasured** binary **confounder** would need to be associated with […] to fully explain our	The use of causal language appears in both abstract and main text. They apply causal methods including target trial emulation and propensity

		by fitting a Cox proportional hazards model, using time since start of follow-up as the time scale and a time independent covariate for treatment assignment. We pooled data from all trials into one model and included each trial as a stratum in the regression (using values from 1 to 252)."	drug treatment use. We then **matched** non-initiators to […] initiators (1:1) by **propensity score** within a maximum **matching** range of 0.025 and without replacement."		findings. […] Finally, an **unmeasured confounder** that was twice as frequent among […] initiators versus among non-initiators would still need to increase the risk of […] by a factor of nine or more to fully explain the results, if no increased risk actually existed (eFigure 3). […] Still, the **emulated trial design** lacked baseline randomisation, and therefore, **unmeasured confounding** cannot be excluded."	score matching and discuss residual confounding.

"To explore **associations** between […] **and later**[…], **overall and by** […] **subtype** and timing of onset."	"[…] was associated with an **increased risk** of […], particularly [subtype]. […] were unlikely to **mediate** the associations substantially, suggesting that […] and […] may share underlying mechanisms or susceptibility pathways. Asking about a history of […] could help physicians to **identify women who might benefit from** screening for early signs of disease, allowing for	"We used Cox regression with age as the underlying time to estimate hazard ratios for […] comparing women with and without a history of […] We used competing risk methods when analysing associations with […] subtypes. […] We evaluated potential **mediation** by […]"	"We considered […] as a priori **confounders**."	"Women with a history of […] had a 53% **increase in risk of** […] overall, compared with women with no history of […] (incidence rate for women with a history of […]: 11.6 per 100 000 person years; incidence rate for women with no history of […]: 8.33 per 100 000 person years; hazard ratio 1.53, 95% confidence interval 1.26 to 1.85)."	"We did sensitivity analyses […] using the array approach for testing the effect of an **unmeasured or incompletely measured confounder.** […] Sensitivity analyses suggested that **confounding** by […] was **unlikely to explain** the observed associations for […]; in contrast, […] **could** conceivably **explain** a considerable part of the association between […] and […]. […] we also cannot rule out the possibility of	Causal language is present in abstract and main text. They apply mediation analysis, adjust for confounders, and discuss the unmeasured confounding assumption and residual confounding.

	early clinical intervention.”				**residual confounding** by other unmeasured covariates”	
“To investigate whether adults with […] **are at an increased risk of** […] and **whether the risk varies by** […] severity and condition activity over time.”	“Severe and predominantly active […] are associated with an increased risk of […] outcomes. **Targeting […] prevention strategies** among these patients should be considered.”	“We used Cox regression stratified by **matched** set […] with current age as the underlying timescale to generate hazard ratios for the association between [...] and each […] outcome (the unadjusted model). Subsequent multivariable analyses adjusted for […] (the **adjusted** model). The adjusted model was further adjusted for variables which may have been on the **causal** pathway (ie, **mediators**) between […] and […] outcomes […] (the **mediation** model). […] The **population attributable risk** of each […] outcome was estimated by using the estimated hazard ratio	“For each patient with […], we **randomly matched** up to five patients by age (within 15 years), sex, general practice, and calendar time at cohort entry. These unexposed patients were required to have at least one year of follow-up in CPRD and no history of […] when matched. […] We used a **directed acyclic graph** to inform the identification	“Table 3 shows that in the primary analysis, there was evidence of **associations** between […] and all […] outcomes, except for […]. **Associations were strongest with** […] (hazard ratio 1.25, 99% confidence interval 1.11 to 1.41 in the adjusted model) and […] (1.19, 1.10 to 1.30), with **partial attenuation in the mediation model**. […] The	“Limitations of the study, inherent to most large observational studies, include the possibility for **confounding**, bias, and missing data. […] We **have shown a clinically relevant increase in the risk of** […] outcomes in patients with [...].This increased risk is largely confined to patients with severe or more active […] and persists despite	The causal aim is evident in the abstract because they intend to assess how the risk of the outcome varies when the exposure is modified and the conclusion is to take action given the findings. The main text uses causal language, discusses DAGs, mediators, collider bias

		and assuming the prevalence of […] to be 10%."	of covariates and **mediators** and to avoid **collider bias**"	greatest **population attributable risks** were estimated for […] (2.4%, 1.1% to 3.9%) and […] (1.9%, 1.0% to 2.9%)."	adjusting for potential mediators, including conventional risk factors for […] outcomes. Consideration should be given to **developing prevention strategies to reduce the risk of** […] among patients with severe or predominantly […]"	and provides population attributable risks.
"To **determine the effect of** […] outcome reporting in […] on risk averse clinical practice, "gaming" of clinical data, and 90 day	"This study **did not find evidence that** the introduction of […] in […] **has led to risk** averse clinical practice behaviour or	"We used a **change point analysis** to study the change over time in **adjusted** 90 day mortality after […] and after […]. We used a multivariable logistic regression model for 90 day mortality, with a slope for calendar time and an interaction	"The **risk factors** included in this logistic regression model are […]. An adjusted outcome was then produced by indirect **standardisation**"	"The 90 day mortality in patients undergoing an […] fell during the study period from 952/33 638 (2.8%) before the introduction of […] to 552/25	"If we assume that the decrease in mortality can be **causally linked** to […], the process of […] This team response could have been	Causal language is present in abstract and main text as they state that their aim is to determine the effect of the exposure on

postoperative mortality.”	“gaming” of data. However, **its introduction coincided with** a significant reduction in 90 day mortality.”	between time pre-introduction versus post-introduction of […], in addition to all of the **risk adjustment variables**. This modelled a change in the slope of mortality at the point that […] was introduced but no immediate change in mortality.”		905 (2.1%) after (fig 4). Therefore, we carried out change point analysis which showed a steeper decline in 90 day mortality after the introduction of […] (P=0.03). The **change point analysis also found a significant effect of** […] when it was modelled as an immediate shift in 90 day mortality (P=0.01) and when it was modelled as both an immediate shift and a change in slope (P=0.04).”	**mediated** through […]”	the outcome and conclude that the exposure has not led to the outcome. The main text explains that confounder adjustment was made through standardisation and discuss possible mediators for this relationship.
“To assess the **effectiveness of**	“Little evidence was found of a	“We estimated one year **net survival** for each […]	“Survival estimates for all	“One year survival	“The lack of **consistent** results	Causal language is

the […] policy initiatives **in improving** […] **and reducing** […] in survival in England."	**direct impact** of […] on one year survival, and no evidence for a reduction in […] in cancer survival. These findings emphasise that […] in survival remain a major public health problem for a healthcare system founded on equity."	by sex, year of diagnosis (1996 to 2013), and deprivation category. Patients with a diagnosis between 1996 and 2013 had the potential to be followed up for at least one year, so we used the classic cohort approach. […] We estimated net survival using the consistent nonparametric estimator defined by Pohar-Perme."	ages combined were age **standardised** with the International Cancer Survival Standard weights. […] We used multivariable linear regression to investigate the survival patterns for each […] and by sex"	**improved** for 20 of the 21 […] examined in women and 16 of the 20 […] examined in men. […] For these […], the average annual absolute increase in one year age **standardised net survival** was often greater than 1% over the whole study period"	between men and women, as well as the lack of general patterns across […] types, provide **little evidence for any strong impact** of the […] policies on short term […] survival. The evidence is even weaker for their **impact** on the […] in […] survival. […] **These findings should be taken into consideration by** […] policy makers and inform future initiatives."	present in both abstract and main text. The aim is to evaluate the effectiveness of a policy on a given outcome, they provide standardised net survival and suggest to take action given the findings.

To investigate whether **improving** adherence to […] **interacts with** the genetic predisposition to […] in relation to long term changes in […] and […].”	“These data indicate that improving adherence to […] could **attenuate** the genetic association with […]. Moreover, the **beneficial effect** of improved […] on […] was particularly pronounced in people at high genetic risk for […].	“We used multivariable generalized linear models with repeated measures analyses to assess the main **associations of** the […] **and changes in** the […] **with change in** […]”	“We used multivariable models to **adjust for** [...]”	“In general, the […] was **associated with increases in** […] every four years: in the two cohorts combined, each additional […] was associated with 0.02 (SE 0.01) increase in […] and 0.05 (SE 0.03) kg increase in […]”	“[…] **unmeasured or unknown confounders** may also exist. Secondly, because adherence to […] was not randomized, the association between […] and […] may not imply a **causal relation**. Thirdly, the results could be underestimated by potential **reverse causality**. […] Our study provides reproducible evidence from two prospective cohorts of US men and women that **improving […] could attenuate** the […]	Assessing if improving adherence has an effect translates to an intervention that is being assessed. They conclude that there is a beneficial effect and suggest to take action. The main text discusses unmeasured confounding assumption and reverse causality. All of the above is consistent with a causal aim.

					association with […]”	
“To assess the **independent** and joint **associations** of [….] and […] with […] risk and to explore the **benefit of […] in reducing** the […] risk associated with […] and […].	“[…] is an overlooked **risk factor** for […], as important as five major lifestyle factors combined. In this study, […] **contributed** to more than one fifth of the risk	“We calculated the time to event from the date of enrollment to the date of [...] incident or [...] death, death due to causes other than [...], or the end of cohort follow-up (31 December 2008), whichever came first. We used Cox proportional hazards model to estimate	“[…] **adjusting for** [...].”	“A statistically significantly increased risk of incident […] was observed for the eight diseases and markers. Specifically, […] was inversely associated with risk of incident	“More evidence was needed to clarify whether the inverse association was **causal** or related to […]. […] the **dose-response relation**, the exclusion of […] during recent	Causal language is present in abstract and main text. They identify that the exposure contributes to the outcome, after adjusting

	for incident […] and more than one third of the risk for […] death. […] is associated with a nearly 40% **reduction in the […] risk** associated with […].”	hazard ratios and 95% confidence intervals”		[…] in a **dose-response manner**”	follow-up, and further adjustment for […] minimize the likelihood of **reverse causation** and lend support for **causality**. […] Our study uncovered a **substantial impact** of […] jointly on […] risk, which were equally as important as five lifestyle factors combined.”	for covariates. They discuss dose-response relationship and reverse causality.
“To examine the **association** between […] and […] **in later life**, and **determine** whether the maintenance of […] will offset age related […]”	“These results show that […] is **not associated with the trajectory of** […] in late life, but is associated with the acquisition of ability during	“The raw scores from the […] tests were **standardised to** a mean of 100 and a standard deviation of 15 to produce an […] scale. Age at testing was the number of years after participants’ 60th birthdays. We modelled age in this form so that	“Because our sample were all born in the same year and tested at a similar age, a **confounder** for age at entry was not used. We modelled cognitive	“The typical intellectual engagement models for each domain are shown in table 2 and indicated **an expected significant decline** in […]	“In our statistical models, we introduced **possible confounders** available from early life and life course, including […]. We also **controlled for** […]	The causal aim is suggested when the goal is to establish temporal relationship between exposure and outcome, describing the

	the life course. Overall, findings suggest that high performing adults engage and **those that engage more being protected from** relative decline.”	the intercept occurred at age 60 years rather than zero years, such that the calculation for the intercept would represent a realistic adult value rather than one extrapolated 64 years earlier. […] For each model, a probability value of P<0.05 was considered significant.”	performance with a linear mixed model, as a combination of […].”	with age, ranging from −1.09 to −1.31 standard points per year for the […] test and −0.77 to −1.69 for the […] test. […] None of the age×TIE interaction terms were significant, indicating that […] **did not influence the trajectories of** age decline.”	associated with repeated testing. […] significant **associations remained after adjustment for** age, sex, and test practice effects. […] is an **independent contributor to** late life […] and has a unique effect over and above the effect of other life course variables. […] It is, however, impossible for a **causal effect** to be inferred […]”	exposure as a trajectory, providing standardised and adjusted estimates and considering whether it is possible to infer a causal link.
“To evaluate the **associations** of a […] and […] with **incident** […].”	“In this cohort study, […] were **independently associated with incident** […]. These results	“To test the **association** of […] and […] with […] we used Cox proportional hazards models. The duration of follow-up was calculated as time	“Cox proportional hazards models included **adjustment for** age and sex for	“In Cox proportional hazards analysis, the **risk of** […] was higher for those with […]	“The present study provides further support that common […] are **implicated in the development**	The abstract describes that the goal is to establish a temporal relationship

	**emphasise the benefit of** entire populations adhering to […], independent of […] risk."	between the baseline assessment and the first event of either […] or 1 March 2016, which was the end of follow-up for the current data release. Participants who had a […] before a […] occurred were censored at the time of the respective event."	the lifestyle score models. For the models including the genetic score we additionally **adjusted for** the first 10 principal components of ancestry and genotyping batch."	(hazard ratio 1.20, 95% confidence interval 1.08 to 1.34) and […] (1.35, 1.21 to 1.50) **compared wit**h those with a low genetic risk score"	**of** […]. […] The […] was also associated with […], which suggest that the **effect of** the […] on risk of incident […] might at least in part be **mediated by** […]. The effects of […] might differ according to the cause of […], although some […] factors are shared between two or more **causal** factors"	between the exposure and the outcome and suggest to take action given the findings. The main text describes adjusting for covariates and discusses mediation.
"To determine the **longitudinal association** between […] and […]."	"In older adults, a **higher cumulative level of** […] was associated with a **higher likelihood** of […]. These	"We used a Cox proportional hazards model to evaluate the **association between time-varying** […], **adjusting for time-varying covariates** (updated at […] measurement), and the	"We selected covariates and potential **mediators** based on biological interest, current or previously observed	"Figure 1 shows that **after multivariable adjustment for** demographic, lifestyle, cardiovascular risks, dietary	"[…] we excluded participants […] who reported baseline […] (to avoid **reverse causality**; n=195). […] The community	The abstract describes that the goal is to establish a temporal relationship between the exposure and

	findings **support guidelines for** increased dietary consumption of […] in older adults."	likelihood […]. Time at risk was from the first […] measurement until the first […] event or censoring […] or the latest date of adjudicated follow-up in June 2015."	associations with […], and meaningful changes in the exposure risk estimate (±5%). Minimal **adjustments included** age and sex. **Multivariable adjustments** additionally included […]. We used the potential mediators to explore what additional associations could exist to these potential **pathways**."	habits, and other […], higher […] levels were associated with a lower likelihood of unhealthy ageing. Overall, participants in the highest group of […] had an 18% (95% confidence interval 3% to 30%; P=0.001) lower risk of […].Findings were not appreciably altered after adjustment for potential **mediators** (not shown)."	based design improves **generalizability**, and regular physical examinations ensured that demographics and other risk factors were well measured, which may help to minimize **confounding**. […]The possibility of **residual confounding** by imprecisely measured or unknown factors also cannot be excluded for an observational study. […] *Any unmeasured confounders would have to be strongly associated with*	the outcome and suggest to take action given the findings. The main text describes adjusting for covariates and discusses mediation, residual confounding and reverse causality. The statement in italic is a clear causal statement.

					both the exposure and the outcome, conditional on all the variables already in the model. Thus, it seems unlikely that either poorly measured or unmeasured confounders could fully account for our findings."	
"To **prospectively** evaluate the joint **association** of […] and […] with risk of type 2 diabetes risk, and to **quantitatively decompose** this joint association to […]"	"Among female nurses, both […] were associated with a higher risk of […]. **The excess risk of** […] was higher than the addition of risk associated with each individual factor. These findings	"Participants contributed person time from the return of the baseline questionnaire [...] until the date of diagnosis of […], death, loss to follow-up, or the end of the follow-up period (30 June 2012 for the NHS and 30 June 2013 for NHS II), whichever came first. We used **multivariable time dependent** Cox	"Information on **potential confounders** was assessed and updated every other year via the questionnaires throughout follow-up. This information included […] In multivariable analysis, we	"We observed a positive association between duration of […] and risk of […] in both cohorts. Compared with women without rotating night shift work, the pooled **multivariable**	"From a public health standpoint, because 71% of the joint effect could be attributed to an […], our findings underscore the importance of maintaining […].Our findings suggest that	Their aim is to establish a temporal relationship exposure-outcome, they discuss adjusting for confounders, provide excess risk and suggest to take action

	suggest that most cases of […] **could be prevented by** […], and the **benefits** could be greater in […].”	proportional hazards models to estimate hazard ratios and 95% confidence intervals for the associations between […] alone and in combination with […].”	**adjusted for** several **confounding** factors including […] We also examined the **decomposition of the joint effect**: the proportion **attributable** to […]”	**adjusted** hazard ratios for women with 1-5, 5-9, and 10 or more years of […] were 1.11 (95% confidence interval 1.00 to 1.22), 1.28 (1.10 to 1.49), and 1.46 (1.33 to 1.62) (P for trend <0.001) […](table 3). […] The **attributable** proportions of the joint **effect** were 17.1% (95% confidence interval 14.0% to 20.8%) for […] alone, 71.2% (66.9% to 75.8%) for […] alone, and 11.3% (7.3% to 17.3%) for their interaction.”	most cases of […] could be **prevented by** […], and the benefits would be larger in […] “	given the findings to prevent the outcome.
“To assess the **association** between […] and	“In this analysis of nationwide registers from	“Patients were followed from cohort entry to treatment cessation,	“We used an active comparator new-	“Use of […], as compared with […], was	“The findings should be interpreted in the	The goal is to evaluate the association

seven serious **adverse events** of current concern."	two countries, use of […], as compared with […], was associated with an **increased risk of** […], but not with other serious **adverse events** of current concern."	crossover to the other study drug […], the outcome event, death, emigration, or the end of the study period (31 December 2016). We used Cox proportional hazards regression to calculate hazard ratios, analysing each outcome independently. The **absolute risk difference** was calculated as hazard ratio−1 multiplied by the rate in the comparator group."	user study design and controlled for a wide range of potential **confounders** (patient characteristics that might be associated with both the outcome and the decision to initiate a drug) through a non-parsimonious **propensity score model** to minimise the risk of bias, including **confounding by indication** […]. We estimated **propensity scores** by using logistic regression for the probability of […] conditional on the status of 66	associated with an increased risk of […] (hazard ratio 2.32, 95% confidence interval 1.37 to 3.91) and […] (2.14, 1.01 to 4.52) but not with […] (1.11, 0.93 to 1.33), […] (0.69, 0.45 to 1.05), […] (0.89, 0.67 to 1.19), […] (0.99, 0.71to 1.38), or […] (1.16, 0.64 to 2.12)."	context of limitations of observational studies and the uncertainty of the **effect estimates**. […] Therefore, the studies could suffer from compromised **confounding** control, as indicated by the imbalance in […] at baseline between users of […] versus comparators, even after propensity score matching […] Finally, **residual and unmeasured confounding** affecting the findings in our study cannot be ruled out."	with the exposure to adverse events which translates into assessing safety. They discuss adjusting for confounding, resort to propensity score matching and consider residual confounding.

			covariates, defined and selected a priori, including sociodemographic characteristics, comorbidities, comedications, and healthcare utilisation […] We matched [..] and [..] users (1:1 ratio, by country) according to propensity score, by using the nearest neighbour matching algorithm (caliper width 0.2 of the standard deviation of the logit score). Analyses were performed in a pooled dataset of the two countries.”			

"To determine whether patients […] have **fewer** […] and **higher rates** of […] than patients […].	***In results*: "Per 100 000 patients, there were 2999 **fewer** follow-up appointments within 14 days, 26 **excess** deaths, […] **attributable to** […]."<br>"Patients […] are **less likely to** have […] and are at higher risk of […]."	"For all outcome comparisons we report unadjusted and **adjusted** odds ratios (with 95% confidence intervals). **Adjusted** odds ratios were obtained with logistic regression models estimated using generalised estimating equations methods and including all measured patient and hospital characteristics."	"We examined hospital type and several characteristics of patients and admissions: year of Charlson comorbidity index score, socioeconomic status (measured using median neighbourhood income), length of hospital stay, arrival by ambulance, diagnosis, discharged with home support or against medical advice, and previous healthcare usage (emergency department visits, hospital stays, outpatient visits, home care	"Patients […] were **less likely** to have follow-up with a physician within seven days (36.3% v 47.8%, adjusted odds ratio 0.61, 95% confidence interval 0.60 to 0.62) and 14 days (59.5% v 68.7%, 0.65, 0.64 to 0.66). […] Patients discharged during the holiday period were at **increased risk of** death or readmission within 30 days (25.9% v 24.7%, 1.09, 1.07 to 1.10). This was **explained by** an increased risk of return to the	"A confirmatory time-to-event analysis in a **propensity score matched cohort** (see supplementary appendix table 5) showed consistent results (death or readmission hazard ratio 1.08, 95% confidence interval 1.07 to 1.09). […] The differences in outcomes **could not be explained by** observed hospital or patient characteristics, including admission diagnosis. […] the possibility of **confounding** due to unmeasured	The abstract and main text use causal language: they discuss risk attributed to the exposure, provide adjusted estimates using propensity score matching and discuss residual confounding.

			visits). hospital discharge, age, sex, rural residence, ”	emergency department (24.3% v 23.0%, 1.09, 1.07 to 1.10), rehospitalisation (11.8% v 11.4%, 1.06, 1.04 to 1.08), and death (1.5% v 1.5%, 1.06, 1.02 to 1.10) within 30 days”	differences remains”	
“To investigate the **association** between […] and overall and specific types of […].”	“Use of […] is **associated** with a reduction in […] risk in women of reproductive age—an **effect related to duration of use**, which diminishes after stopping use. These data suggest no **protective**	“We calculated age **standardised** incidence rates of […] per 100 000 person years, using the age distribution of the cohort as standard. Risk of […] among users of the different product groups was analysed by a Poisson regression model in SAS version 9.3 (SAS Institute). […] We calculated the population prevented fraction (population prevented fraction =	“**Adjusted** incidence rate ratios (referred to here as relative risks) and their surrounding 95% confidence intervals were calculated for each model, with never users as the reference group. The **adjusted** models included the	“The age **adjusted** incidence of […] was highest in women who were never users of […] (7.5 per 100 000 person years; table 2). Among ever users of […], **reduction** in the age standardised absolute rate of […] was 3.2 per	“The data linkage study design also enabled us to **adjust** for several important **confounding** variables. We were not able to adjust for some factors, such as […] Our findings, therefore, could be subject to **residual confounding**.”	When assessing the exposure-outcome relationship, they provide standardised estimates, discuss residual confounding, dose-response and provide attributable fraction.

	**effect** from […].”	prevalence$_{exposure}$(1−relative risk)) associated with ever use of […] by using the relative risk of never use versus ever use of […]. The population prevented fraction is the proportion (expressed as a percentage) of the […] in the cohort that has been prevented by ever use of […].”	following time varying covariates […]”	100 000 person years. Overall, ever users of […] had a reduced risk of […] compared with never users (relative risk 0.66 (95% confidence interval 0.58 to 0.76)). […] **use of […] prevented** 21% of […] in the study population”		
“To assess whether […] is **associated with a reduction** in […] and mortality in old and very old adults with and without […].”	“In participants older than 74 years without […], […] was **not associated with a reduction in** […] or in all-cause mortality, even when the incidence of […] was	“To prevent survivor bias and covariate measurement bias, we selected a “new users design” over “all […] users. […] Using Cox proportional hazard regression models **adjusted by propensity score**, we calculated the hazard ratios of statin use for the outcome events. Participants were censored at the date of	“We explored the variables associated with […] to determine candidate variables for the propensity score of […]. From SIDIAPQ we obtained data on age, sex, […] Because of non-random	“In participants without […], the hazard ratios for […] were 0.94 (95% confidence interval 0.86 to 1.04) for […] and 0.98 (0.91 to 1.05) for all cause mortality in 75-84 year olds. […] The one year **number needed**	“To prevent **residual confounding** we performed additional regression adjustments after adjustment of **propensity score**. Variables that remained imbalanced after propensity score	They use causal language and causal methods. To estimate the effect of the exposure on the outcome. They use propensity score methods to adjust for

	**statistically significantly higher** than the risk thresholds proposed for […]. In the presence of […], […] was statistically significantly associated with reductions in the incidence of […] and in all-cause mortality. This **effect** decreased after age 85 years and disappeared in nonagenarians. ”	transfer from SIDIAPQ or at the end of the study period. ”	treatment allocation, we used a logistic model based on **potential confounding** covariates to calculate the **propensity score** of […]. We calculated the propensity score separately for participants with and without […] and also within each age group, and standardised differences before and after adjustment for **propensity score**. Variables with standardised differences <0.10 were considered to be **well balanced**.”	**to treat** was 164 for […] and 306 for all cause mortality.”	adjustment were also included in the models. […] Despite these efforts, we acknowledge that some **residual confounding** might exist.”	confounding, provided a NNT and consider residual confounding.

"To examine the **association** between [...] and risk of [...]."	"The risk of […] was increased in people who […]. In several countries, guidelines define thresholds for […]. The present findings **encourage the downward revision of such guidelines** to promote […] at older ages."	"Cox regression was used in all analyses, with age as the timescale to model the associations with hazard of incident […]. Participants were censored at date of record of […], death, or 31 March 2017, whichever came first."	"Models were first **adjusted** for sociodemographic factors, then additionally for health behaviours, and finally for health status."	"[…] was associated with a **higher risk** of […] when the reference was […]; in these analyses […] was associated with an increased risk of […] in a linear fashion (among those […], P for non-linearity=0.97 using spline regressions). In a model **adjusted for** sociodemographic factors […] was associated with a **greater risk** of […] (hazard ratio 1.47, 1.15 to 1.89) compared with […]"	"[…] multistate models showed that part of the excess risk of […] in […] was **attributable to** the greater risk of […] in this group. […] We accounted for several sociodemographic and health related characteristics in the analysis, but **residual confounding** cannot be excluded as an explanation for the higher risk of […] among […]. Our multistate models lent partial support for a **mediating** role of […] in the association	The abstract and main text use causal language, they provide adjusted, discuss residual confounding and suggest to take action given the findings.

					between […] and increased […]”	
“To assess whether **adding or switching to** […] is associated with an increased risk of […], **compared with remaining** on […] in patients with […].”	“[…] as second line drugs are associated with an increased risk of […] compared with remaining on […]. Continuing […] when introducing […] appears to be **safer** than switching.”	“The study cohort was formed by identifying all subjects from the base cohort of […] initiators who subsequently added or switched to a […] as second line treatment. Patients who added or switched to other […] were censored. For each patient adding or switching to a […], we identified a matched reference patient who also was a […] initiator but remained on metformin, using a prevalent new-user design. […] we constructed a Cox proportional hazards regression model for each outcome that estimated the hazard ratio and the 95% confidence intervals for […] versus […].”	“[…] exposed and reference subjects were **matched on high-dimensional propensity score**. The high-dimensional **propensity score method** empirically selects covariates based on their prevalence and potential for **confounding**. For each member of each matched set, we identified all available information from seven data dimensions (five dimensions from the CPRD: drug prescriptions,	“Compared with the use of […], adding or switching to […] was associated with an increased risk of […] (7.8 v 6.2 per 1000 person years, hazard ratio 1.26, 95% confidence interval 1.01 to 1.56), all cause mortality (27.3 v 21.5, 1.28, 1.15 to 1.44), and […] (5.5 v 0.7, 7.60, 4.64 to 12.44). There was also a trend towards increased risks of […] (6.7 v 5.5, 1.24, 0.99 to 1.56) and […] (9.4 v 8.1, 1.18, 0.98 to 1.43).”	“Based on a post-hoc analysis, the findings of the primary analysis on […] unlikely to be the result of an **unmeasured confounder** under most plausible exposure-confounder and confounder-outcome associations. […] For our study, we used the recently developed prevalent new-user design. To **emulate** the randomised controlled trial, this design identifies (at the doctor visit that	The aim is to assess the effect of the intervention strategy of adding or switching to a particular drug, they emulate a target trial, use propensity score matching, discuss residual confounding and conclude that the strategy is safe.

			procedures, diagnoses, disease history, and administrative information; two dimensions form the HES: diagnoses and procedures) in the one year period before the date of the **matched** set. We then applied conditional logistic regression to estimate the **propensity** of receiving a […] drug, thereby considering the 500 most likely **confounders**."		led to the patient on […] adding or being switched to […]) a comparable patient with the same history of […] use and of other characteristics, but who on that visit continued on […]. […] owing to its observational nature there is the potential for **residual confounding**."	
"To investigate the **associations** between […] and	"Overall, […] was found to be the **safest**	"We used a new-user design to capture all events occurring after	"**Confounding** factors. It is possible that	"In patients with […], […] was associated with a	"Although many adjustments have been done using	The aim is to assess the safety of a

**risks of** […] compared […].”	drug, with reduced risks […] compared with […]. […] and low dose […] were, however, associated with increased risks of all cause mortality compared with […].”	starting treatment and to reduce the impact of **confounding**. […] Incidence rates for each outcome were calculated based on the numbers with the outcome and the person years of follow-up, and were age and sex standardised for each drug. To estimate the risks associated with each […], an outcome specific Cox model containing all **confounding** factors was used, with […] as a primary reference.”	patients at higher risk of […] may preferentially be prescribed […] rather than […], so all analyses were **adjusted for** demographic and clinical variables, either because they may have been used as indicators for prescribing a specific […] or because they have possible associations with increased risk of […]. We similarly adjusted for comorbidities, previous events, and drugs also used as indicators or associated with increased risks.”	lower risk of […] than […] (adjusted hazard ratio 0.66, 95% confidence interval 0.54 to 0.79). […]Table 5 shows the **number needed to treat** or **number needed to harm** to measure the relative benefits or risks of […] in comparison with […].”	the data available on the existing databases, there is a possibility of **unmeasured confounding** or **confounding** by indication. […] Although we used a proportional hazard model adjusting for all available **confounding** factors, we also undertook a sensitivity analysis using the **propensity score method** and obtained very similar results.”	drug and they use different strategies to adjust for confounding, including propensity scores, provide NNT and discuss the unmeasured confounding assumption.

"To examine the **association** between […] and the **risk of** developing […]."	"Our study indicates that adherence to a […] is **associated with a substantially reduced risk of** […]. These findings highlight the **potential benefits of implementing** […] interventions to curb the risk of […]."	"To evaluate the **association** between […] and […], we calculated relative risks and 95% confidence intervals using multivariable log-binomial regression models with generalized estimating equations and specified an exchangeable correlation structure."	"We first evaluated associations with […] by categories of each low risk factor, **adjusting** for […]."	"In multivariable analyses (model 2), […] had a **relative risk** of 3.10 (95% confidence interval 2.69 to 3.57) of […], compared with […]."	"Several factors could contribute to the weak **mediation effect** of […] in the association between […] and […] risk. […] Another limitation, as in any observational study, is that we cannot exclude the possibility of **uncontrolled confounding** by […] or **residual confounding**. […] Our findings highlight the potentially critical role of […] in the **etiology** of […] and lend support to […] based **intervention strategies** for reducing […]."	They provide adjusted estimates, discuss mediation and residual confounding and suggest to take action given the findings.

"To determine **rates of** [...] and all cause mortality in patients with [...] compared to patients with [...] and without [...]."	"Patients with [...] remain at higher risk of [...] than patients without [...]. The **risk** is increased even in those in whom [...] is not documented. **Guidelines** should be updated to advocate continued use of [...] in patients with [...]"	"We carried out two retrospective cohort studies to determine incidence rates of [...] (primary outcome) and all cause mortality (secondary outcome) in patients with [...] versus randomly selected **matched controls** with [...]. We calculated crude and **adjusted** incidence rate ratios comparing the incidence of [...]"	"Poisson regression was used to calculate **adjusted** incidence rate ratios, adjusting for the baseline covariates [...]"	"The crude incidence rate ratio was 0.73 (95% confidence interval 0.65 to 0.81, P<0.001). Adjusting for potential confounders [...] made little difference to the incidence rate ratio: 0.76 (95% confidence interval 0.67 to 0.85, P<0.001)"	"In light of the evidence produced by this study, it is recommended that clinical guidelines and schemes designed to incentivise appropriate management [...] are updated"	Even though the use of causal language is not explicit, they compare rates of the condition in the different groups that have been matched, provide adjusted estimates and suggest to update guidelines to reflect the findings.
"To examine the **association** between [...] at [...] and [...]."	"[...] during the period [...] is **safe** with respect to the risk of [...]."	"We estimated odds ratios of [...] and [...] and associated Wald type two sided 95% confidence intervals by logistic regression. For [...] and [...], we calculated hazard ratios and associated	"[...] to adjust for potential **confounding** due to temporal trends, we included [...]. [...] are well established risk	"In analyses without covariate adjustment (model 1), [...] was **associated** with an increased risk of [...] (hazard ratio 1.69	"[...] is **not causally related** to increased risks (…). Instead, our results suggest other factors underlying and confounding the	The abstract suggests a causal aim when describing the intention to establish a temporal

		Wald type two sided 95% confidence intervals from Cox regression models, which allow for detailed adjustment for censoring affecting the length of follow-up of each child. Days since birth was used as the underlying time scale. Each child was followed from birth until a diagnosis of the outcome, death, or end of follow-up at 31 December 2014, whichever event occurred first. ”	factors for [...]. [...] All estimates were calculated by models with increasing complexity, beginning with models without adjustment for covariates (model 1), followed by models adjusting for all included potentially **confounding** covariates (model 2).”	(95% confidence interval 1.18 to 2.41)) and [...] (2.14 (1.39 to 3.30); fig 2). After covariate **adjustment** (model 2), [...] was only associated with an increased risk of [...] (adjusted hazard ratio 1.66 (1.06 to 2.59); fig 2).”	associations between […]. Furthermore, although our results suggest that […] are not causally associated […] could be a causal factor for other outcomes. [...] although the present study did not find a causal link […], replication of the results is imperative.”	relationship between the exposure and the outcome and concluding that the exposure is safe. The main text uses causal language explicitly when describing the strategies to control for confounding and concluding that a causal relationship was discarded.
“To assess the association of [...] and risk factors for [...] with [...] at [...].”	“The **independent association** between [...] and [...] in [...] is comparable	“We used a generalised additive mixed model (GAMM) to estimate [...], with [...] as fixed effect predictors and [...] as random effect at the	“We considered [...] as potential confounders.”		“As our analyses relied on cross sectional data, these findings should be interpreted	The abstract suggests a causal aim when describing the intention to

	in strength and consistency with those for [...]. The results of this study suggest that **tackling** all these risk factors might substantially increase life years spent in good physical functioning.”	intercept and [...] slope. [...] We computed 95% confidence intervals from the uncertainty of the estimated smoothing function. We computed the **number of years of functioning lost** from the mixed model predictions”			cautiously and should not be considered as causal estimates of the impact of [...] on [...]. [...] Given that the present study is based on observational data, our study informs about associations but cannot provide evidence of causality.”	establish a temporal relationship between the exposure and the outcome and concluding that the link is comparable to those for other established risk factors. It is important to note that In the abstract the design of the study is described as “Multi-cohort population based study”. However the method and discussion refer to a “cross sectional” design that

						limits the possibility to establish causal links.
"To determine outcomes and **safety** of [...] for [...], due to [...], in routine clinical practice."	"In routine clinical practice, [...] for patients with [...] is at least as **effective** and **safe** as in the setting of a randomised controlled trial."	"We used regression models to compare baseline characteristics and outcomes in patients [...] with those in the [...] intervention and control arms. The effect of [...] on [...] at 90 days in patients [...] compared with [...] was expressed as an **adjusted** common odds ratio, derived from multivariable ordinal logistic regression (shift analysis)."	"We **adjusted** for [...]"	"After **adjustment** for [...], the shift towards [...] was significant for patients [...] compared with those [...] intervention arm (adjusted common odds ratio 1.30, 95% confidence interval 1.02 to 1.67; P=0.03) and control arm (1.85, 1.64 to 2.34; P<0.01; fig 1)."	"The results of our study might have important implications for the future of [...] for [...]. [...] is at least as **effective** and **safe** as in the setting of a randomised controlled trial."	The abstract and main text point to a causal aim as the intention is to assess the safety of an exposure in relation to an outcome and the conclusion is that not only is safe but also effective.
"To investigate whether [...] is **associated** with an **increased risk** of [...]."	"In a **propensity score matched** cohort, [...] use was associated	"Cox proportional hazards regression, with days since start of treatment as the time scale, was used to estimate the hazard ratio	"We used two major strategies to control for **confounding**. To account for	"There was an increased risk of [...] associated with [...] (hazard ratio 1.66; 95%	"An important concern in any observational study is the possibility of	Both the abstract and main text use causal language and

	with an increased risk of [...]. This association appeared to be largely **driven by** [...].”	for [...], comparing episodes of [...] and [...] use.”	potential **confounding** by indication [...], we used an **active comparator design**, [...] To control for potential **confounding** from differences in baseline health status, we used a **propensity score matched** design, taking into account demographic characteristics, medical history, [..].”	confidence interval 1.12 to 2.46). This increase corresponded to an absolute difference of 82 (95% confidence interval 15 to 181) cases of [...] per 1 million treatment episodes in the 60 day risk period.”	confounding. We used an active comparator to limit confounding by factors associated with [...], including confounding by indication, and propensity score matching derived from a range of covariates. Despite this, the possibility of residual confounding (for example, due to [...]) cannot completely be ruled out.”	causal methods including propensity score matching. They discuss the possibility of residual confounding mainly because of the observational nature of the study but also suggest possible confounders missed.
“To examine the **risks** of [...] in patients with [...] and in a general population comparison cohort.”	“[...] was associated with increased risks of [...]. [...] may be an important risk factor for [...].”	“We did a population based **matched cohort** study based on routinely and prospectively collected data. [...] We calculated the 0-1 year, >1-5 years, and >5-19	“Using the full hospital history (inpatient and outpatient diagnoses) recorded in the DNPR before the	“After adjustment for the covariables, [...] was associated with [...] (adjusted hazard ratio 1.49,	“Although we and adjusted the analyses for a wide range of potential **confounders** identified a priori	Both the abstract and main text describe the exposure-outcome relation in a

		years cumulative incidence per 1000 people for each outcome, accounting for the competing risk of death. Correspondingly, we used matching factors stratified (conditional) Cox proportional hazards regression to estimate hazard ratios, adjusting for the categorical comorbidities listed above as covariables.”	index date, we obtained information on the following [...] risk factors: [...]”	95% confidence interval 1.36 to 1.64), [...] (2.26, 2.11 to 2.41), and [...] (1.94, 1.68 to 2.23), as well as [...] (1.59, 1.45 to 1.74) and [...] (1.25, 1.16 to 1.36) (fig 2). We found no association with [...] (adjusted hazard ratio 1.12, 0.96 to 1.30) or [...] (1.04, 0.93 to 1.16).	on the basis of excisting literature, we cannot exclude influence of unknown or **residual confounding**, for example, by [...]” ** Typos copied as in the published version	matched cohort. They discuss the possibility of residual confounding and suggest possible confounders missed. All of these elements point to a causal aim.
“To determine if [...] a critical determinant of [...] is and [...].”	“[...] does not have a **clinically important association** with [...] or [...].”	“We assessed the effect of [...] compared with [...], using multivariable regression. Modified Park’s tests were used to determine the appropriate regression models (gamma, Poisson, and logistic) for discrete [...] outcomes. We also assessed the effect of [...]	“In all of our primary analyses we adjusted for the following key **confounders**: [...]”	“Table 2 shows that there was no strong evidence of a **clinically important association** of [...] and [...] with [...] or [...].”	“We recognise that we assessed multiple associations and the isolated positive association of [...] and [...] may reflect a chance finding, particularly as	Both abstract and main text use causal language and explain that the aim is to identify whether the exposure is a cause of the outcome and

		on an individual's repeat [...] outcomes scores. [...] Linear mixed effects models were fitted with time as a fixed effect and a random effect of subject."			there was no consistent association with [...] at any other [...]. [...] We would suggest the overall impact would be potentially small as there was no **clinically important impact** on [...] at any age. We acknowledge that [...] may have attenuated to the null any potential detrimental effect of [...] on [...] outcomes, but this would further support that [...] **does not have** permanent consequences for [...]."	after adjusting for potential confounders conclude that it is not, given that they only identify one positive association when multiple were assessed and consider it to be by chance.

"To determine if [...] is **associated** with an increased risk of [...] in the general population."	"[...] is **associated** with a **greater risk** of [...] compared with [...], but not a greater risk of death. The relative risk increase is similar across population groups, but the **higher baseline risk** among those [...] **translates into higher absolute risks of** [...] in these groups."	"We calculated odds ratios for each outcome [...] within 14 days of [...] comparing each [...] adjusting for potential **confounders** using logistic regression."	"Based on a priori knowledge, we considered the following variables as potential **confounders** of the relation between [...] and [...]: [...]. All covariates other than sex and ethnicity were updated over time. [...] We initially adjusted for sex and age only, and then fitted an adjusted model using [...]."	"In the 14 days after [...], [...] is associated with the highest odds of [...] (adjusted odds ratio 1.72, 95% confidence interval 1.31 to 2.24) and [...] (2.27, 1.49 to 3.45) of all the [...] investigated. [...] The odds of death within 14 days of [...] were similar to [...] for [...] (0.90, 0.76 to 1.07) and the other [...]."	"We saw minimal differences in the odds ratios for […]. [...] analyses using multivariable regression and inverse probability treatment weighting approaches […] were consistent. [...] our study also had greater ability to adjust for detailed characteristics, such as [...], which are likely to have reduced **residual confounding**. "	The elements that point to a causal aim: confounder adjustment by regression models, sensitivity analysis using inverse probability of treatment weighting and discussing residual confounding.
"To evaluate the [...] **safety** of [...], in direct comparisons with	"In this large cohort study, [...] was associated with a **lower risk** of	"For each comparison and for all outcomes, we calculated unadjusted and **propensity score matched** number of events,	"We considered the following covariates as potential **confounders**: [...]	"Table 3 shows that after **propensity score matching**, for [...] primary	"Randomized controlled trials are the best way to assess drug efficacy […] On	Both abstract and main text use causal language and they are

[...], as used in routine practice."	[...] l and with a **similar risk** of [...] in direct comparisons with [...] as used in routine care."	incidence rates, and hazard ratios with 95% confidence intervals."	To control for imbalances in patient characteristics between cohorts, we calculated exposure **propensity scores** as the predicted probability of receiving the treatment of interest (ie, [...] v each comparator) conditional upon the subjects' baseline covariates using three separate multivariable logistic regression models. All variables were included and no further selection was conducted.	outcome, the number of events for [...] and the [...] comparator were 91 and 124 respectively (8.9 v 12.8 per 1000 person years; hazard ratio 0.70, 95% confidence interval 0.54 to 0.92) in cohort 1; 94 and 148 (7.5 v 12.4; 0.61, 0.47 to 0.78) in cohort 2; and 77 and 154 (7.3 v 14.4; 0.51, 0.38 to 0.67) in cohort 3."	the other hand, strict inclusion and exclusion criteria and rigorous safety monitoring limit the **generalizability** of randomized controlled trial results. Our study [...] allowing better generalizability to routine care [...] provides data from direct comparisons. [...] while we used **propensity score matching** to balance more than 100 baseline characteristics between the groups, **residual confounding** by some unmeasured	explicit to state that the aim is to evaluate safety of the exposure and use causal methods (propensity score matching). They discuss why the design was observational and consider that due to this nature, residual confounding cannot be excluded that may have led to downtown of conclusion which is phrased more in terms of association.

			We 1:1 **matched** cohorts on their **propensity score** using a caliper width equal to 0.2 of the standard deviation of the logit of the propensity score.”		characteristic(s) cannot be ruled out. ”	
INCONSISTENT						
“To evaluate the **relation between** […] and development of […]”	“[…] was **associated with** an increased risk of […] that was **mediated by** […]. **Systematically addressing** […] may be an important public health strategy to reduce the incidence of […]	“We calculated the hazard ratios for the relation of […] to the risk of MRSA using Cox proportional hazard models. […] We also calculated the absolute risk difference. […] We performed **mediation analyses** to examine the extent to which the effect of […] on the risk of […] was through […]. Using **marginal structural models** we then estimated the **natural**	“We performed a **matched cohort** study […] **matched on** age (one year either way), sex, and study entry time (within one year either way). Such comparators were chosen to further ensure the comparability […] In the multivariable Cox	“The matched and multivariable **adjusted** hazard ratios for patients with […] were 1.69 (1.51 to 1.90) for […] and 1.26 (1.12 to 1.40) for […].”	“Our GP practice based dataset could have missed the detection of some inpatient cases of […]; however, these potential **non-differential misclassifications** would have biased our results towards the null, rendering our	The aim in the abstract limits to state that they are exploring the relationship of exposure and outcome. However, in the abstract conclusion and full text they describe mediation analysis,

	among patients wih […].”	**direct effect** […] and the **natural indirect effect** […] while adjusting for the same confounding variables”	model we **adjusted for** […].”		findings conservative”	identifying direct and indirect effects and use marginal structural models which are part of the causal toolkit.
“To quantify the **effects of** varying […]”	“[…] is **associated with** a large increase in […] among […] patients. The data from this study suggest that […] rather than […] is more strongly associated with […]”	“For **adjusted** analysis of time until […] we used Cox proportional hazards models.”	“**Adjusted** models included […]”	“Each additional […] **increased the rate of** […] by 70.7% (95% confidence interval 54.6% to 88.4%) before adjustment and increased the hazard of […] by 44.0% (40.8% to 47.2%, P<0.001) after adjusting for covariates.”	“To determine the extent to which **strong unobserved confounding** might explain the observed association, we included this synthetic confounder in a Cox model. […] As part of a sensitivity analysis, we constructed models that removed **potential**	The aim uses causal language and they provided adjusted estimates. However the conclusion is phrased in terms of association. They do use sensitivity analysis to test for residual confounding and it might be that due to concern of

					**confounders.** […]"	unmeasured confounding they decided to be conservative with the conclusions.
"To evaluate the long term **association** between […]"	"Widespread utilisation of […] may be **contributing to** long term increased risk of […]. The potential for […] **should be considered** when […]."	"We conducted Poisson regression analyses using person years as observations."	"We included several variables as known **confounders** or effect modifiers in the relation between. […]The final fully adjusted model adjusted for […]"	"After **adjustment** for covariates, the rate ratio was […] indicating that during the entire period of follow-up the risk of […] was 21% higher during […] than at other times."	"The registered active […] population is generally representative of the UK population in terms of age, sex, and regional distribution"	The aim is phrased in terms of association but the conclusion uses causal language and they discuss confounder adjustment.
"To investigate the **association** of […]"	"The shape of the **association** between […] and […] was **determined by** […].This finding suggests that the […] may be **largely**	"We used Cox proportional hazards models to estimate hazard ratios and 95% confidence intervals. We stratified the analysis by age in months and calendar year of the questionnaire cycle."	"For the main analysis, we used […] measured at baseline to minimize the effect of underlying diseases on mortality […] In multivariable	"A multivariable **adjusted** model showed a positive association between […] and all cause mortality, whereas […] showed a U	"Our findings remained robust in several sensitivity analyses […] we cannot entirely rule out the possibility of **unmeasured or unknown**	The aim is stated in terms of association but they adjust for confounders, discuss unmeasured confounding and conclude

	**explained by** […]”		models, we **adjusted for potential confounders** including […]”	shaped association with all cause mortality. In a mutually adjusted model including both […] and […] we consistently observed a **strong positive association** between […] and all cause mortality.”	**confounding** factors that may account for the associations observed in this study.”	that the outcome can be ‘largely explained’ by the exposure.
“To examine the **associations** of […]”	“This association could be **explained by** the finding that […] These results emphasise the importance of revisiting […] or establishing specific guidelines for	“We performed Cox models with penalised splines”	“In final Cox models with penalised splines, we made **adjustments** for: […]”	“[A]fter **adjustment for confounding** factors, the U shaped association with […]”		The aim is phrased in terms of association but they provide adjusted estimates, discuss confounding and conclude that the exposure

	management among […]"					could explain the outcome and suggest to take actions given the findings.
"To **estimate long term survival**, health, and educational/social functioning in patients with […]"	"[…] had **no substantial effect** on […]"	"We calculated mortality rate ratios and incidence rate ratios as measures of **relative risk**."	"For each […] patient, we used the Danish Civil Registration System and the DNPR to identify all Danish residents with the same sex and date of birth as the patient who had not tested positive […] and who met the study's inclusion and exclusion criteria […]. From this population, we extracted 10 people at random for each patient. People in	Patients and members of the comparison cohort were **well matched** with respect to […] Mortality was not higher among patients in the […] cohort"		The abstract states that they aim is to estimate survival but they use matching and conclude that the exposure has no 'substantial effect' on the outcome.

			the population comparison cohort were assigned the same date of study inclusion as […] patients to whom they were **matched**.”			
“To **compare** the risk of […]”	“Although **residual confounding** cannot be excluded, this finding deserves consideration when […] is used for […]”	“We estimated the crude hazard ratio of […] using Cox proportional hazard regression, and the adjusted hazard ratio was obtained using **propensity score matching**”	“ We identified **potential confounders** that were plausibly associated with both […]based on clinical knowledge […] In the context of this study, the propensity score is the probability of receiving […] as opposed to […], given the baseline characteristics. Patients who received […]	“The crude hazard ratio of death in the unmatched cohort was 1.51 (95% confidence interval 1.22 to 1.85) and the adjusted hazard ratio in the matched cohort was 1.50 (1.14 to 1.96)”	“Comparison of the baseline characteristics in the unmatched cohort provided little evidence of confounding […] it is unlikely that a few additional **unmeasured** variables can explain a 50% increase in the risk independent of all other confounder and proxies of **confounders** that	The abstract suggests that they aim is comparison of the risks but does not explicitly use causal language. They do adjust for confounding, using propensity score matching, and discussed unmeasured confounding which are

			were matched to patients who […] using a 1:1 nearest neighbor matching algorithm with a caliper of 0.2 of the standard deviation of the propensity score on the logit scale. Covariate balance between the two groups was assessed after matching, and we considered an absolute standardized difference less than 0.1 as evidence of balance”		are adjusted for in our study.”	applied when aiming for causal inference.
“To **determine** whether […] is associated with […]”	“[…] was independently **associated** With […]”	“We fitted both a mixed effect logistic regression model (in which the outcome was defined as	“We examined the relation between […] and	“The rate of distinct criteria met per year increased by 24%	“We did a sensitivity analysis using **propensity score**	The aim uses causal language but the conclusion

		dichotomous […] and the Prentice, Williams, and Peterson (PWP) model"	[…] **adjusted** for …"	if a patient had been admitted to hospital (hazard ratio 1.24, 95% confidence interval 1.20 to 1.28) when **controlled** for the other covariates"	**matching** to assess whether the association between […] and […] could be due to **unmeasured confounders** […] Although we adjusted for a range of characteristics of patients, as with any observational study potential exists for **unmeasured confounding**, which may partly or fully explain the relation between […]"	is phrased in terms of association. They provide adjusted estimates, use propensity score as sensitivity analysis and discuss unmeasured confounders. The only reason to present a conservative conclusion seems to be the observational nature of the study.
"To investigate associations between […] and to analyse the	"Risks of […] are inversely **associated** with […]"	"We used multivariable Cox regression analysis to **compare** the rates of […] and […]. "	"**Confounders** included in the final models were based on the literature or	"Compared with […], […] had increased hazard ratios of […]"	"We believe that our findings are widely applicable and provide justification for	The aim suggests a causal aim because they evaluate the

**effect of changes** […]"			statistical significance (P<0.10). The full model included […]"		[…] and continuing […]"	impact of changing the exposure which makes it an intervention and they provide estimates adjusted for confounders. The conclusion is phrased in terms of association.
"To estimate the **rates of** […]"	"In cases of […], approximately […] will become […], of which a third will have […]."	"We present denominators where data for the secondary outcome are missing. We defined the population **attributable fraction** as (Re–Run)/Re=(RR–1)/RR, calculated using Stata. To test the robustness of our findings, we did a sensitivity analysis."	"We compared the demographic and clinical variables of […]. We used the binomial Wilson score to calculate confidence intervals of single proportions and the Pearson exact method to calculate	"[…] had a higher risk of […] The population **attributable fraction** of […] was 47% for […] and 61% for […]"	" Considering these results when **counselling** potentially exposed […] seems reasonable"	The aim and conclusion are phrased in association terms. However, they estimate attributable fractions and suggest to act given the findings.

			confidence intervals of risk ratios and medians."			
To perform an expedited **assessment of** […] **risk** associated with exposure to […]".	"The results **do not imply** a markedly increased short term overall risk of […] in […]."	"We used Cox regression to estimate the hazard ratio with 95% confidence intervals for […] associated with […], both for ever use and for the predefined categories of cumulative use"	"Analyses were, however, performed as crude comparisons **adjusted** only for […] as well as adjusted for […] and the **potential confounding** factors."	"Overall, exposure to […] showed no association with […] compared with exposure to […] (adjusted hazard ratio 1.09, 95% confidence interval 0.85 to 1.41) and no evidence of a dose-response relation"	"This ensured that the estimates were not affected by **immortal time bias** […]. As all comparisons were performed within users of […], the exposure to […] can reasonably be expected to be a random event, and **confounding** is thus expected to be limited."	The aim does not use causal language. They do provide estimates adjusted for confounding, discuss immortal time bias and conclude that the exposure does not result in an increase survival.
"To investigate the **risks** of […] in […]"	"No increased risk of […] was detected in […], but increased risks of […] were found in this study. Our	"To calculate expected […], we multiplied the person years at risk by corresponding national incidence rates (by 5 year age band and individual calendar year) for the	"We obtained data relating to **potential confounding** factors such as […]"	"There was no overall increased risk of […] (2578 observed v 2641.2 expected […]; standardised incidence ratio	"Given previous inconsistent results, small study size, and lack of information on **potential**	The aim does not use causal language. They provide standardised estimates, discuss

	results suggest that […] **risks could be due to** […], **rather than** […].”	general female population of England and Wales. **Standardised incidence ratios** were calculated by the comparison of observed values with expected values.”		0.98 (95% confidence interval 0.94 to 1.01); absolute excess risk −2.8 cases per 100 000 person years (95% confidence interval −7.1 to 1.8); table 2)”.	**confounders**, we undertook a population based linkage study in […]”	confounding and conclude that the risk of the outcome is due to a given exposure compared to another.
CONSISTENTLY NOT CAUSAL						
“To determine whether […], compared […], is **associated** with an increased risk of […]”	“In this population based cohort study, […] was **associated** with an increased risk of […]. The association was particularly elevated among people using […] for more than five years. **Additional studies**, with	“We calculated crude incidence rates of […] and 95% confidence intervals, based on the Poisson distribution, for each exposure group. We used time dependent Cox proportional hazards models to estimate hazard ratios and 95% confidence intervals of […] associated with […] compared with […], using multiple imputation for variables with missing values.”	“Potential **confounders**. All models were **adjusted** for the following variables measured at cohort entry: […]. […] as an alternate means of controlling for **confounding**, we repeated the analysis by stratifying the model on tenths	“Compared with […], […] were **associated** with an overall 14% **greater risk of** [..] (1.6 v 1.2 per 1000 person years; hazard ratio 1.14, 95% confidence interval 1.01 to 1.29).”	“We introduced a one year exposure lag period to account for a minimum latency time window and to minimize **reverse causality**. […]The association between […] and […] is **biologically plausible**. […] although we were able to adjust for several	In the abstract they only describe associations but in the full text their interest points to a causal aim given the different methods applied to adjust for confounding and reverse causality. They

	long term follow-up, are needed to investigate the **effects** of […] on [...].”		of disease risk score. Finally, we repeated the analysis using a **marginal structural** Cox proportional hazards model using **inverse probability of treatment** and censoring weighting—a method designed to adjust for time dependent **confounding** associated with time varying exposures”		important **confounders**, this study lacked information on other potential **confounders** such as […] In this large, population based study, […] was associated with an elevated risk of […] overall, along with evidence of a **duration-response relation.**”	also consider elements as biologically plausibility and duration response relation. Residual confounding seems to be a concern because they lacked information on relevant cofounders which could lead to down tone of the conclusions.
“To determine whether […] and […] are **associated** with an **increased risk of** […] in adults with […].”	“Compared with […], […], and […], **might be associated** with an increased risk of […] in adults with […].”	“For each exposure category we calculated crude incidence rates of […] with 95% confidence intervals, based on the Poisson distribution. Time dependent Cox proportional hazards	“The models were **adjusted** for the potential **confounders** measured at cohort entry: […] To minimise potential	“Compared with […], […] was **associated** with a 77% increase in the hazards of […] (hazard ratio 1.77, 95% confidence	“Finally, we excluded those with less than one year of follow-up after cohort entry, to allow for a sufficient latency	The abstract only refers to association but the full text mentions adjusting for confounders and ways to

		models were used to estimate hazard ratios and 95% confidence intervals of […] associated with […] and […], separately, when compared with […].”	**confounding** by indication, we compared […]. […] we fit a **marginal structural model** to investigate the impact of potential time dependent **confounding** using **inverse probability of treatment** and censoring weighting.”	interval 1.04 to 3.01).”	period and to minimise **reverse causality**. […] To assess possible **duration-response relations**, we investigated the association between cumulative duration of […] on the risk of […]. An association between […] and incidence of […] is **biologically plausible.** […] as with all observational studies, **residual confounding** is possible. We conducted several sensitivity and ancillary analyses specifically designed to	minimise reverse causality. They also describe its biological plausibility. There is a concern for residual confounding due to the observational nature rather than missing information on particular relevant confounders.

					assess the potential impact of **residual** confounding.”	
“To examine the **association** between […] and […] risk of […].”	“[…] **could increase the risk of** […]. However, confirmation of these findings are warranted, preferably in an intervention setting”	“[…] was categorised by percentiles (<10, 10-20, 20-50, 50-80, 80-90, ≥90). […] With these same categories of exposure, the association between […] and […] was examined by Cox regression. We used […] age from birth up to May 2016 as the underlying timescale censoring if death or emigration from Denmark occurred (1217 events).”	“Characteristics that might influence the risk of [….] were identified a priori and included as potential **confounders** in our **adjusted** analysis. In model 1, we adjusted for: […].In model 2, additional adjustments were made for […]”	“[…] was significantly associated with **increased risk of** […] in both unadjusted and covariate **adjusted** analyses (table 3). Compared with […], offspring of those with […] had double the risk of […] during follow-up (hazard ratio 2.00 (95% confidence interval 1.02 to 4.00)). Risk of […] was positively **associated** with […]: the **association** was	“[…] the mechanism that might be responsible for this **effect** is not known, but could include […]. […] the role of unmeasured or unidentified confounders can never be fully excluded in observational studies.”	The abstract only considers associations but the full text mentions confounder adjustment and discusses potential mechanism (biological plausibility). Concern of residual confounding is due to the observational nature of the study.

				significant (Ptrend=0.016) and increased monotonically. Only minor differences were observed between the unadjusted and covariate **adjusted** analyses.”		
“To ascertain compliance **rates** with […]; to identify features **associated** with non-compliance; to **rank** […] by compliance; and to build a tool for live ongoing audit of compliance.”	“Compliance with […] has been poor, with half of all […] non-compliant. […] commonly contain inconsistencies that might prevent even […] assessing compliance. Accessible and timely information on the compliance	“We constructed a logistic regression model with all these **explanatory** variables, as they were selected prospectively on the basis of clinical and methodological interest.”	“**Explanatory** variables. We created variables for a range of features of each […], selected prospectively on the basis of clinical and methodological interest.”	“In the **adjusted** multivariable analysis, […] with a […] were **significantly more likely to** […] (adjusted odds ratio 23.3, 95% confidence interval 19.2 to 28.2); as were […] (18.4, 15.3 to 22.1).”	--	Although adjusted estimates are present, both abstract and full text limit to describe associations, rates and ranks. No causal language is used.

	status of […] and […] may help to improve reporting **rates**."					
"To assess **how often** older adults […] were […], and to identify markers of […]."	"**One in seven** older adults […] were […]. **More than half of** […] occurred in patients with […]. More attention is needed to reduce potentially harmful […] as older adults […]."	"We did multivariable mixed effect logistic regression analyses to determine **associations** between the outcome of […] and primary **predictors** of […]."	"Our primary **predictor** variables were […]. **Adjusted** analyses included the covariates noted above, a random effect term to account for clustering by hospital, and an interaction term to account for the relation between […] and […]."	"A total of 2074 (14%) patients were […]; 1293 (9%) were […] and 300 (2%) were […]. Additionally, 628 (4%) patients were […]. […] Patients with […] had a 25% (95% confidence interval 23% to 78%) probability of […]."	--	The abstract indicates that the aim is to assess the frequency of a condition and that is reflected in the main text. No causal language used.
"To describe **trends** in the **rate** and daily dose of […] used among […] from 2007 to 2016."	"[…] **rates** were high during the study period of 2007-16, with the **highest rates** in […] versus […] and	"Endpoints were defined at the person quarter level. We used logistic regression to model the **proportion** of the population […] each quarter. The average […]	"All analyses were stratified by beneficiary category including commercially insured, aged	"Averaged across the entire study period, 51.5% of disabled Medicare beneficiaries […] per year (n=1 128	--	The abstract indicates that the aim is to describe the frequency of a condition which is

	[…]. […] and average daily dose have not **substantially declined** from their peaks, despite increased attention to […] and awareness of their **risks**."	per person day by quarter was modelled by a generalized linear model with negative binomial family and log link. The dependent variable was the total […] per person in the quarter, with an exposure variable representing the number of days of insurance coverage for each person included to **standardize** daily […]."	Medicare, and disabled Medicare (beneficiaries with Medicare coverage who were under age 65 years)"	088), compared with 14.3% (n=18 721 915) of commercial beneficiaries and 25.7% (n=3 847 676) of aged Medicare beneficiaries."		reflected in the main text. No causal language used.
"To **describe** […] related mortality in the United States during 1999-2016 by age group, sex, race, cause of […], and geographic region."	"Mortality due to […] has been **increasing** in the US since 2009. Driven by deaths due to […], people aged 25-34 have experienced the **greatest relative increase** in mortality.	"Our primary aim was to describe temporal trends in death rates attributable to […] and […] as the primary or underlying cause of death for adults in the USA.[…] We then evaluated trends in death rates using the National Cancer Institute's Joinpoint program. This enabled us to identify if there were years in the study period where the	"We adjusted rates for age—that is, age specific mortality was weighted according to the age distribution in a standard year (2000). We also sought to describe how these trends differed based on demographic	"During the study period, a total of 460 760 deaths were attributed to […] (20 661 in 1999 and 34 174 in 2016) and 136 442 to […] (5112 in 1999 and 11 073 in 2016) (table 1). Men had a higher **burden** of age **adjusted**	"[…] though we have detected worsening mortality since 2009, the precise reasons for this trend and the geographic heterogeneity in our analysis require further study. For example, we identify the	The abstract indicates that the aim is to describe the frequency of a condition which is reflected in the main text. No causal language used.

	White Americans, Native Americans, and Hispanic Americans experienced the **greatest increase** in deaths from […]. Mortality due to […] is improving in Maryland but worst in Kentucky, New Mexico, and Arkansas. The rapid increase in death rates among young people due to […] highlight new challenges for optimal care of patients with […].”	rate of change in mortality was statistically significantly different. The program uses a piecewise linear regression approach to determine whether rates over time are best described by a straight line (0 joinpoints) or by multiple linear segments (≥1 joinpoints)”	subgroups; age, sex, race (Asian or Pacific Islander, Native American (designated as “American Indian” in the census database) or Alaska Native, black or African American, and white American), Hispanic ethnicity, and geographic area of residence.”	mortality due to […] compared with women by a 2:1 ratio and a higher **burden** of mortality due to […] by a nearly 4:1 ratio.”	states most at risk, but granular data are needed to determine the root causes”	

"To examine whether […] are **associated** with an increased risk of […] after […]."	"Women with […], especially […], may be at **higher risk of** […]. If these findings are replicated elsewhere, a massive amount of data exists that could aid in identifying women at **higher risk of** […] and that could be conveyed to them or their healthcare providers."	"The main model assessed the primary […] composite outcome, as well as the individual outcomes of […], in relation to each […] for the screened cohort, with censoring at a woman's death or arrival at the end of the study period of 31 March 2016, allowing for a maximum follow-up of 22 years. We did time to event analyses using multivariable Cox regression models, to derive a hazard ratio and 95% confidence interval for each study outcome."	"Hazard ratios were **adjusted** for variables chosen a priori, based on the existing literature, including: […]"	"A total of 6209 women developed the primary […] composite outcome, which was typically about 1.2 to 1.3 times **more likely** to occur in a […], even after **adjustment** for other covariates"	"Potential **confounders** between […] and the risk of […], including […] were each accounted for in the models. Nevertheless, about 10% of […] lacked information on […], and […] and […] were entirely unknown."	The abstract indicates that the aim is to identify the association between an exposure and an outcome. They consider residual confounding due to lack of information on relevant confounders.
"To examine the **association** between [...] and the risk of [...] according to levels of [...]."	"Among [...], increasingly worse [...] was **associated** with a **progressively increased** risk of [...]. Even	"Using generalised linear models with a robust sandwich estimator, we estimated risk ratios for [...], comparing [...] according to levels of [...] with [...]. To take into	"Analyses were **adjusted** for […]"	"In analyses based on [...] levels, the **adjusted** risk ratios for [...] were 2.17 (95% confidence	"It is also possible that the previously demonstrated **association** [...] has resulted in increased clinical	The abstract indicates that the aim is to identify the association between an exposure and

	with [...] within target levels recommended by guidelines [...], the risk of [...] was increased more than twofold. The risk of [...] was not statistically significantly increased at any of the [...] levels examined; the study had limited statistical power for this outcome and was based on [...] only.”	account possible dependence from repeated [...], we constructed models with [...] as a cluster variable. [...] were assumed to follow a poisson distribution, and we estimated risk ratios using a log link function”		interval 1.37 to 3.42) for [...], 3.17 (2.45 to 4.11) for [...], 2.79 (1.90 to 4.12) for [...], and 6.23 (4.32 to 9.00) for [...] versus [...]. The corresponding **adjusted** risk differences were 17 (95% confidence interval 5 to 36), 32 (21 to 46), 26 (13 to 46), and 77 (49 to 118) cases per 1000 [...].”	surveillance for defects among [...]. [...] health registers do not record data on [...] and hence we could not account for these factors.”	an outcome. They consider residual confounding due to lack of information on relevant confounders.
“To examine the **association** between risk factor burdens—categorized as	“Regardless of index ages at 55, 65, or 75 years, an optimal **risk**	“We calculated the lifetime risks for the first incident [...] from index ages 55, 65, and 75 years up to age 95 years. [...] we	“We computed lifetime **risk in subgroups** of participants according to their	“The associated lifetime risk of [...] was lowest if the risk factor profile was	“Our observational study design limits the ability to establish	The abstract and main text indicate that the study mainly aims at

optimal, borderline, or elevated—and the lifetime risk of [...].”	**factor** profile was associated with a lifetime risk of [...] of about one in five; this risk rose to more than one in three in individuals with at least one elevated risk factor.”	used a modified Kaplan-Meier estimator with age as the time scale, accounting for the competing risk of death to compute the lifetime cumulative risk of [...] and associated 95% confidence intervals”	risk profile at a specified index age (optimal, borderline, and elevated), for each **risk factor** separately and for the combination of **risk factors**. [...] we fitted a multivariable Fine and Gray model, **adjusted** for competing risk of death to **predict** the lifetime risk of [...].”	optimal. The lifetime risk of [...] increased gradually as the risk factor profile changed from optimal to borderline and elevated at each index age.”	**causal pathways**, and only **associations** between **risk factor** profiles and lifetime risk of […] can be concluded from our study.”	identifying associations. There is a concern for residual confounding due to the observational nature rather than missing information on particular relevant confounders.
“To compare **rates** of [...] for patients [...], with patients [...].”	“[...] was **associated** with lower [...] **rates** compared with [...].”	“We used a Cox proportional hazards regression model, adjusting for (…), to estimate hazard ratios and 95% confidence intervals for [...] comparing [...] with [...]. To summarize switchback estimates	“[...] **adjusting** for basic demographics (age, sex, and calendar year)”	“Figure 5 shows that in the adjusted analysis, the [...] rates remained consistently lower among [...] than [...]. The magnitude of this	“[...] our results indicate that […] may in part be **driven by** […].”	Even though causal language is not used and both the abstract and main text mainly describe associations,

		across [...], we conducted inverse variance weighted random effects meta-analyses"		effect was largest for [...] (hazard ratio 0.52, 95% confidence interval 0.43 to 0.63) and smallest for [...] (0.86, 0.77 to 0.97). The pooled hazard ratio across [...] suggested that [...] was associated with a 28% lower rate of [...] compared with [...] (0.72, 0.64 to 0.81)."		their conclusion at the end of the text suggests a causal relationship.
"To assess whether [...] is **associated** with the incidence of [...] in patients with [...]."	"In this first population based study, [...] was **associated** with an increased risk of [...]. Although these findings need to be	"We calculated crude incidence rates of [...] with 95% confidence intervals based on the Poisson distribution for the entire cohort and for each exposure group. For all analyses, we used time dependent Cox proportional hazards	"The models were **adjusted for** the following potential **confounders** measured at cohort entry: [...]"	"Compared with [...], [...] was **associated** with a 75% **increase in risk** of [...] (53.4 v 34.5 per 100 000 per year; hazard ratio 1.75, 95% confidence interval 1.22 to	"[…] as with all observational studies, **residual confounding** from unknown or unmeasured variables remains possible. However, on the basis of the rule	The abstract mainly describes associations but in the main text they describe how their estimates were adjusted for potential

	replicated, physicians should be aware of this possible association.”	models to estimate hazard ratios and 95% confidence intervals for [...] associated with [...] compared with [...]. We also calculated the **number needed to harm** for patients followed over a two year and four year period by using methods accounting for varying patient follow-up times.”		2.49). The **number needed to harm** corresponded to 2291 patients followed over a two year period and 1177 over a four year period.”	out method, a hypothetical confounder would need to be strongly associated with both the exposure (odds ratio >4.7) and the outcome (relative risk >5.0) to move the point estimate towards the null.”	confounders and they estimate numbers needed to harm (NNH). Also they consider residual confounding and suggest that only a strong unmeasured confounder will remove the association observed. All of these, point to a causal analysis.
“To assess the prospective **associations** between [...] and risk of [...].	“In this large prospective study, a 10% increase in the proportion of [...] associated with a	“We used Cox proportional hazards models with age as the primary timescale to evaluate the association between [...] and incidence of [...]. In these	“Models were **adjusted** for [...] we made additional adjustments [...]. In addition we	“In model 1, [...]. was associated with increased risks of overall cancer (hazard ratio for a 10 point increment	“Lastly, although we included a large range of **confounding factors** in the analyses, the hypothesis of	The abstract mainly describes associations but in the main text they describe how

	**significant increase** of greater than10% in risks of [...]. Further studies are needed to better understand the relative effect of the various dimensions of [...] in these associations."	models. We estimated hazard ratios and 95% confidence intervals with the lowest quarter as the reference category."	did **mediation** analyses [...]"	in the proportion of [...] 1.12 (95% confidence interval 1.06 to 1.18), P<0.001) and [...] (1.11 (1.02 to 1.22), P=0.02). ."	**residual confounding** resulting from unmeasured factors (...) cannot be entirely excluded owing to the observational design of this study"	their estimates were adjusted for potential confounders and they use mediation analysis which suggest a causal aim. They also consider residual confounding due to the observational nature of the study.
"To assess the **association** between [...] and all cause mortality in [...] with [...]."	"Giving [...] to [...] with [...] was **associated** with an increased rate of [...] but a paradoxical lowered rate of all cause mortality. Careful	"We calculated the incidence of [...] and all cause mortality per 100 person years of follow-up. We generated Kaplan-Meier survival curves for the outcomes of interest grouped by [...] status. Cox proportion regression were reported as **adjusted** hazard ratios with 95%	"We used propensity score matching with demographic and clinical variables to adjust for potential confounding from imbalances in clinical characteristics	"The crude rates for [...] and [...] were 4.6 and 1.2 after [...], and 1.5 and 0.4 in patients who [....] per 100 person years, respectively. In the Cox proportion	"The study population was derived from real world evidence with the inherent limitations of diagnostic coding and case ascertainment Despite well **matched** groups	The abstract limits to describe associations and rates of the condition but the main text suggest a causal aim as they use propensity

	consideration should be given before [...]. There remains an urgent need for adequately powered randomised trials in this population to explore these findings and to provide clarity on correct clinical management.”	confidence intervals. Because all baseline characteristics were balanced in the **propensity matched** model, [...] was the only independent variable in our Cox regression model.”	between patients receiving and not receiving [...]. Demographic variables were [...]. Clinical variables were [...]. Patients who received anticoagulation were matched in a 1:1 ratio”	regression models, the hazard ratios for [...], [...] and all cause mortality for patients [...] were 2.60 (95% confidence interval 2.00 to 3.38), 2.42 (1.44 to 4.05), and 0.82 (0.74 to 0.91) compared with those who [...].”	after **propensity score matching**, we cannot exclude that the reported associations were **confounded** by indication”	score matching to control for confounding.
“To assess the **temporal change** in [...] and to identify [...] with the greatest increase in use.”	“Total [...] use has increased markedly over time, in both sexes, and across all age groups [...]. Of the patients who [...], the **proportion** who had [...] increased	“For comparison across years, we **standardised** crude rates to the mid-2015 UK population. Rates were calculated for [...]. Joinpoint regression was used to model the temporal changes in age and sex **standardised** rates”	“For each year we calculated the total number [...], **stratified** by age and sex. We calculated total person years of observation in each age and sex stratum for each year.”	“The age and sex **adjusted** rate of [...] increased from 14 869 tests per 10 000 person years in 2000/1 to 49 267 in 2015/16 (table 1, fig 1), an annual increase of 8.5% (95% confidence	“We analysed the temporal change in [...] from UK primary care. The total use of tests increased markedly over time, even after adjustment for population growth”	The abstract and full text state that they aim to quantify changes of a condition over time. It is a descriptive study that does not use causal

	significantly over time.”			interval 7.6% to 9.3%). [...] The slope of the trend line changed significantly at two points: 2004/5 (P<0.001) and 2008/9 (P=0.004) (fig 1).”		language accordingly.
“To investigate the **association** of [...] with disease specific incidence and mortality and whether [...] enhances the **prediction** ability of an established office based **risk score**.”	“[...] was **associated** with a range of health outcomes and improved **prediction** of an office based **risk score**. Further work on the use of [...] in risk scores or risk screening is needed to establish its potential clinical utility.”	“[...] we investigated the associations of [...] with cause specific incidence and mortality over follow-up with Cox proportional hazard models. We reported the results as hazard ratios together with 95% confidence intervals.”	“We treated [...] as potential **confounders**. For Cox proportional hazard analyses, we ran four models that included an increasing number of covariates: model 0 (minimally adjusted) included [...]”	“As shown in figure 1, in both men and women, [...] was **associated** with a higher hazard for all cause mortality and incidence of and mortality from [...] in model 0. The **associations** were similar after **adjustment** for [...] in model 1; after further adjustment, the magnitude of	“To minimise the potential contribution of **reverse causality** to the findings, we did a landmark analysis excluding events occurring within the two years after recruitment in model 4 (landmark analysis). This landmark analysis was adjusted as in	The abstract mentions associations and suggests that the aim is prediction. In the main text, the authors conclude that the exposure of interest enhances prediction and identification of patients with risk of certain diseases.

				associations were slightly attenuated in models 2, 3, and 4”	model 3. [...] may, therefore, be a useful method of **identifying** people with [...] who are at high risk of a wide range of diseases. [...] **Reverse causality** is possible in any observational study. [...] Similarly, **residual confounding** is always possible and the associations observed may not imply **causality**. However, given that we are largely interested in **prediction** and identification of people at	However, there is use of causal language, including confounder adjustment and discussing reverse causality and residual confounding. They note that their goal is to do prediction and that reverse causality is not a major limitation but still adjust for it.

					increased risk, and not seeking to make strong causal inferences, **reverse causality** is not a major limitation. ”	
“To **externally validate** four commonly used rules in [...] for [...].”	“Application of the [...] rules can lead to a wide variation in [...] among patients with [...], **resulting** in many unnecessary [...] findings. Until an existing decision rule has been updated, any of the four rules can be used for patients presenting [...] at the	“The sensitivity, specificity, and proportion of patients [...] (with 95% confidence intervals) were assessed for each of the four decision rules. [...] The Cochran’s Q test was used to directly compare the sensitivities and specificities between the four decision rules [...]. Net proportional benefit has been proposed to incorporate such weighting in calculation of clinical usefulness of decision rules. For each rule, we expressed the net proportional benefit using the formula: (true	--	“The sensitivity for identifying patients with [...] ranged from 72.5% for the [...] criteria to 98.8% for the [...] rule (table 4; appendix 3). [...] The [...] criteria would have missed 11 of 74 patients with [...] (appendix 4). The CHIP criteria would have missed two patients with [...], who both had [...]. The	--	Both the abstract and the full text state that they aim to validate four decision rules for a particular condition. They compared the tests in terms of sensibility or specificity and concluded that the tests are similar and recommended the use of a particular one

	emergency department. Use of the [...] rule is recommended because it **leads to** a substantial reduction in [...] while missing few potential [...].”	positives/total number) − weight × (false positives/total number). ”		specificity for identifying [...] was lowest for the [...] rule (4.4%) and highest for the [...] criteria (60.9%). [...] The sensitivity and specificity differed significantly between all the rules (Cochran’s Q P<0.001). ”		given that it can help avoid false negatives. The wording ’resulting’ and ‘leads to’ are in fact to discuss the potential for false positives/ false negatives rather than a casual claim.
“To develop and **validate** a set of practical **prediction tools** that reliably estimate the outcome of [...].”	“The **prediction** models reliably estimate the outcome of patients who were managed in various settings for [...]. The **predictor** items are readily derived at hospital admission. The	“The **association** between predictor variables and [...] was analysed by fitting proportional odds logistic regression models **adjusting** for the fixed effect of study. Prognostic strength was quantified as odds ratios with 95% confidence intervals. The relative importance of each **predictor** in the models was estimated	“In a published systematic review, we identified relevant **predictors** of outcome in patients [...]. Based on the results of this published review, we selected the following	“Bootstrap resampling showed negligible model optimism. The models had **internally validated AUCs** between 0.77 and 0.83. There was no significant lack of fit (goodness of fit	--	The abstract and main text state that the goal of the study is to validate a prediction tool. Consistent with the prediction aim, no causal

	web based [...] **prognostic** calculator [...] and the related app could be adjunctive tools to support management of patients."	with partial R2 statistic, which estimates the independent **contribution** of the **predictor** to the variance of the outcome"	**predictor** variables that are assessable early at hospital admission and are consistently associated with outcomes for inclusion in the **prediction** models: [...]"	P≥0.2 in all models). Cross validated performance was variable across studies [...]. The partial R2 values ranged between 4% and 46%, and the pooled AUC values were between 0.74 and 0.77"		language is used.
"To prospectively **validate** [...] to triage patients with [...] in routine clinical practice."	"In a population of patients referred for [...], this new triaging approach accurately **classified** [...] for most, with half the utilisation of ABPM compared with usual care. This	"To examine model performance, we constructed a logistic regression model with true [...] as the dependant outcome variable and classification using [...] as the independent predictor variable. From this model we estimated the **area under the receiver operating characteristic (AUROC) curve** statistic."	--	"The triaging strategy [...] predicted true [...] (true positives 66%, 95% confidence interval 63% to 69%; true negatives 24%, 22% to 27%) with a low error rate (false positives 8%, 6% to 10%; false negatives 2%, 1% to 3%)	--	The abstract and main text describe that the aim is to validate a triage tool and assessed its performance compared to the standard of reference. As the aim is prediction, no causal language is

	triaging strategy can therefore be recommended for diagnosis or management of [...] in patients where [...] is being considered, particularly in settings with limited resources.”			(table 2). The triaging strategy resulted in 49% (46% to 52%) being referred for [...] and the remainder managed on the basis of their clinic measurements.”		used accordingly.
