# Supplementary material for: Consistency of causal claims in observational studies: a review of papers published in a general medical journal
Source: BMJ Open. 2021 May 20;11(5):e043339. doi: 10.1136/bmjopen-2020-043339 (PMC8141434; doi:10.1136/bmjopen-2020-043339)
Supplement: Author's manuscript [file bmjopen-2020-043339.draft_revisions.pdf]

BMJ Open

BMJ Open is committed to open peer review. As part of this commitment we make the peer review history of every article we publish publicly available.

When an article is published we post the peer reviewers' comments and the authors' responses online. We also post the versions of the paper that were used during peer review. These are the versions that the peer review comments apply to.

The versions of the paper that follow are the versions that were submitted during the peer review process. They are not the versions of record or the final published versions. They should not be cited or distributed as the published version of this manuscript.

BMJ Open is an open access journal and the full, final, typeset and author-corrected version of record of the manuscript is available on our site with no access controls, subscription charges or pay-per-view fees (http://bmjopen.bmj.com).

If you have any questions on BMJ Open's open peer review process please email info.bmjopen@bmj.com

BMJ Open

Use of causal claims in observational studies: a research on research study

Journal:	*BMJ Open*
Manuscript ID	bmjopen-2020-043339
Article Type:	Original research
Date Submitted by the Author:	02-Aug-2020

Complete List of Authors:	Olarte Parra, Camila; Ghent University, Applied Mathematics, Computer Science and Statistics; University of Amsterdam, Clinical Epidemiology, Biostatistics and Bioinformatics<br>Bertizzolo, Lorenzo; INSERM, U 1153, Equipe Methods; University of Amsterdam, Clinical Epidemiology, Biostatistics and Bioinformatics<br>Schroter, Sara; BMJ, Editorial<br>Dechartres, Agnes; Hopitaux Universitaires Pitie Salpetriere-Charles Foix, Departement biostatistique sante publique et information medicale<br>Goetghebeur, Els; Ghent University, Applied Mathematics, Computer Science and Statistics

Keywords:	STATISTICS & RESEARCH METHODS, EPIDEMIOLOGY, EDUCATION & TRAINING (see Medical Education & Training)

SCHOLARONE™
Manuscripts

I, the Submitting Author has the right to grant and does grant on behalf of all authors of the Work (as defined in the below author licence), an exclusive licence and/or a non-exclusive licence for contributions from authors who are: i) UK Crown employees; ii) where BMJ has agreed a CC-BY licence shall apply, and/or iii) in accordance with the terms applicable for US Federal Government officers or employees acting as part of their official duties; on a worldwide, perpetual, irrevocable, royalty-free basis to BMJ Publishing Group Ltd ("BMJ") its licensees and where the relevant Journal is co-owned by BMJ to the co-owners of the Journal, to publish the Work in this journal and any other BMJ products and to exploit all rights, as set out in our licence.

The Submitting Author accepts and understands that any supply made under these terms is made by BMJ to the Submitting Author unless you are acting as an employee on behalf of your employer or a postgraduate student of an affiliated institution which is paying any applicable article publishing charge ("APC") for Open Access articles. Where the Submitting Author wishes to make the Work available on an Open Access basis (and intends to pay the relevant APC), the terms of reuse of such Open Access shall be governed by a Creative Commons licence – details of these licences and which Creative Commons licence will apply to this Work are set out in our licence referred to above.

Other than as permitted in any relevant BMJ Author's Self Archiving Policies, I confirm this Work has not been accepted for publication elsewhere, is not being considered for publication elsewhere and does not duplicate material already published. I confirm all authors consent to publication of this Work and authorise the granting of this licence.

For peer review only

Use of causal claims in observational studies: a research on research study

Camila Olarte Parra[1,2], **Lorenzo Bertizzolo**[2,3], **Sara Schroter**[4], **Agnès Dechartres**[5], **Els Goetghebeur**[1]

1. Department of Applied Mathematics, Computer Science and Statistics, Ghent University, Ghent, Belgium

2. Department of Clinical Epidemiology, Biostatistics and Bioinformatics, Academisch Medisch Centrum, Amsterdam, The Netherlands

3. INSERM, U1153 Epidemiology and Biostatistics Sorbonne Paris Cité Research Center (CRESS), Methods of therapeutic evaluation of chronic diseases Team (METHODS), Paris, F-75004 France; Paris Descartes University, Sorbonne Paris Cité, France.

4. The BMJ, London, United Kingdom

5. Sorbonne Université, Institut National de la Santé et de la Recherche Médicale (INSERM), Institut Pierre Louis d'Epidémiologie et de Santé Publique, AP-HP. Sorbonne Université, Hôpital Pitié Salpêtrière, Département de Santé Publique, F75013, Paris, France

Correspondence to: Camila Olarte Parra
Department of Applied Mathematics, Computer Science and Statistics
Ghent University, Ghent, Belgium
Campus Sterre, S9, Krijgslaan 281, 9000 Ghent, Belgium
Email: camila.olarteparra@ugent.be, colartep@gmail.com

For peer review only

Abstract

Objective: To evaluate the consistency of causal statements in the abstracts of observational studies published in The BMJ.

Design: Research on research study.

Data source: All cohort or longitudinal studies describing an exposure-outcome relationship published in The BMJ during 2018. We also had access to the submitted papers and reviewer reports.

Main outcome measures: Proportion of published research papers with 'inconsistent' use of causal language in the abstract. Papers where language was consistently causal or non-causal were classified as 'consistently causal' or 'consistently not causal', respectively; those where causality may be inferred were classified as 'suggests causal'. For the 'inconsistent' papers, we then compared the published and submitted version.

Results: Of 151 published research papers, 60 described eligible studies. Of these 60, we classified the causal language used as 'consistently causal' (13%), 'suggests causal' (35%), 'inconsistent' (20%) and 'consistently not causal'(32%). The majority of the 'Inconsistent' papers (92%) were already inconsistent on submission. The inconsistencies found in both submitted and published versions was mainly due to mismatches between objectives and conclusions. One section might be carefully phrased in terms of association while the other presented causal language. When identifying only an association, some authors jumped to recommending acting on the findings as if motivated by the evidence presented.

Conclusion: Further guidance is necessary for authors on what constitutes a causal statement and how to justify or discuss assumptions involved. Based on screening these abstracts, we provide a list of expressions beyond the obvious 'cause' word which may inspire a useful more comprehensive compendium on causal language.

Strengths and limitations of this study

- We present examples of ambiguous causal statements in published abstracts of observational studies in a high impact journal
- We focused on the abstract where clear messages are especially important, as many readers just read the abstract of a study
- The focus on the abstract may miss further discussion on the validity of underlying assumptions justifying causal inference in the setting studied.
- The prevalence and nature of the problems found is a call for better instruction on and consideration of causal language throughout the editorial process in clinical and epidemiological research.
- We provide a list of words and study elements that could point in the direction of causality or otherwise, which may inspire a more comprehensive compendium.

For peer review only

Introduction

Many researchers remain tempted to draw causal conclusions from observational data despite acknowledging that mere association is not causation because causal inference is the ultimate goal of most clinical and public health research (1, 2). Gold-standard answers are typically sought through randomized controlled trials (RCTs). The unique ability of RCTs to avoid confounding bias (3) has led to demands that empirical research must be drawn from randomized studies to justify causal statements (4-6). RCTs are mainly used to assess the effect of a treatment or intervention but are not easily adapted to evaluate prognostic or risk factors rather than interventions.

There are however good reasons to look beyond RCTs for evidence on treatment effects. In many settings, RCTs are not feasible, ethical or timely and thus observational data are all that is available for some time, as in the recent COVID-19 crisis. Furthermore, observational studies typically involve broader real-world contexts than RCTs, where the costs and risks of experimentation suggest studying high risk patients without major comorbidities (7). This selection challenges generalization to the target population. Trials further suffer from treatment non-compliance which complicates analysis, as treatment-specific populations lose the benefit of randomization. Recent ICH9 guidelines therefore emphasize the importance of causal estimands beyond intention-to-treat, such as per-protocol and as-treated analysis (8, 9).

Deliberately avoiding causal statements on a hoped-for causal answer brings ambiguity and contrived reporting (10, 11). Instead, authors should openly discuss the likely distance in meaning and magnitude between the data based measure they are able to estimate and the desired targeted causal effect. Arguments would consider study design with additional assumptions in context (12). Owing to decades of progress in statistical science (involving potential outcomes, directed acyclic graphs, propensity scores and more) (13), this allows for results, often unreachable by randomized trials, with a justified causal interpretation (14).

In 2010, Cofield et al (5) assessed the use of causal language in observational studies in nutrition but deemed causal language inappropriate for all observational studies. From a different angle, Haber et al (15) examined whether the tone and strength of causal claims made in a given paper matched the language describing the findings in social media. Not surprisingly they found stronger causal statements in the media in half of the cases, emphasizing the importance of clear scientific messages.

To promote this, Lederer et al (16) recently published a guide for authors and editors on how to report causal studies in Respiratory, Sleep and Critical Care Journals. Rather than circumventing the problem by asking to avoid causal language, they provide key elements that ensure valid causal claims (17). Besides briefly explaining causal inference, they provide a definition of a confounder, outline how to identify confounding through so-called directed acyclic graphs and discuss how p-values are often misinterpreted and how their value does not reflect the magnitude, direction or clinical importance of a given association. All these elements empower their target audience to critically assess observational studies.

To find out whether and how statements in study reports present confusing use of causal language (or lack thereof), we examined abstracts of research papers concerned with exposures and outcomes published in The BMJ in 2018. Our focus was on the causal message The BMJ readers receive from the abstracts. We evaluate the consistency of causal statements in the abstracts of observational studies published abstract and if any a priori changes had been made as a result of the peer review process.

For peer review only

Methods

Sampling and inclusion criteria

COP identified all original research articles published in The BMJ in 2018 described as either cohort or longitudinal studies in the study design of the abstract. The eligible studies were identified by statements in this section of the abstract such as "cohort", "longitudinal" or "registry-based". Those identified as "observational" were included if they suggested a period of follow-up rather than being cross-sectional. Articles described as case-cohorts were excluded as their interpretation and analysis differs from other studies with follow-up assessing the exposure-outcome relationship.

Assessment of published abstracts

Two reviewers (COP, LB) independently screened all the abstracts of the eligible papers. For the text included under each of the subheadings in the abstract (objective, design, setting, participants, outcome, results, conclusion), the reviewers assessed whether there was an (implicit) causal claim using a yes/no/unclear response. After assessing each separate subheading, each reviewer then gave an overall assessment of the main claims in the paper's abstract as either 'consistently causal', 'suggests causal', 'inconsistent' or 'consistently not causal'. After the independent assessments, the overall rating of the abstract was compared between both reviewers; where there was disagreement, a third reviewer (EG) was consulted and a consensus reached.

Assessment of submitted versions

As the focus of this paper is the avoidance of misleading and ambiguous messages, we further assessed those articles judged as 'Inconsistent' to see if there were changes introduced to the manuscript between submission and publication. For this subset, we obtained the submitted version of the manuscripts and the associated peer reviewers' comments from The BMJ's manuscript tracking system. We then compared the published version with the first submitted version to identify whether the wording related to causal claims appeared in the submitted version of the abstract and whether changes occurred as a result of comments from peer reviewers and editors.

The same reviewers (COP, LB) independently evaluated the submitted versions of the papers. The reviewers assessed whether the content under each subheading of the submitted abstract differed from the published version. Where there were discrepancies between versions, each reviewer indicated the presence of a causal claim as yes/no/unclear for each abstract subheading (title, objective, design, setting, participants, outcome, results, conclusion) and made an overall assessment of the submitted abstract as either 'consistently causal', 'suggests causal', 'inconsistent' or 'consistently not causal'. As before, the assessments were compared and, in cases of disagreement, a third reviewer (EG) was consulted and consensus reached.

Assessment of full text

For the published papers classified as 'inconsistent', we further evaluated the full published text to identify the statistical method applied. We look for statements that would support a causal aim, including confounding adjustment, sources of bias and issues of generalizability.

For peer review only

Ethics and consent

This study used routinely collected data. When authors and reviewers submit manuscripts and reviews to The BMJ, they are notified that their paper or review may be entered into research projects for quality improvement purposes. COP was given access to The BMJ's data under a confidentiality agreement.

Patient and public involvement

Patients were not involved in the design, analysis or interpretation of the study. Patients were not participants in this study; it was a methodological study (research on research). Patients' opinions of causal statements and the use of ambiguous language in research papers is important and further work in this area partnered by patients is important.

Results

Assessment of published abstracts

In 2018, 151 research papers were published in The BMJ, of which 60 (40%) were eligible for inclusion in our study (Figure 1). We identified 29 studies (48%) using causal language ('consistently causal' and 'suggests causal'). A further twelve (20%) abstracts were considered inconsistent mainly because the objective stated evaluating an association while the conclusion presented a causal finding (9/12) or the opposite (3/12). Finally, there were abstracts that described studies that aim for prediction or reported associations without (implicitly) suggesting that they had a causal nature that were considered consistently not causal (n=19, 32%).

Tables

Table 1 shows excerpts from the abstracts that were evaluated. Each row corresponds to statements from the same study. The first column indicates the assigned category, based on the type of association it describes. The last column explains why a given abstract was considered to belong to the assigned category. As the assessment pertains to causal claims in general, the words referring to the particular topic of the corresponding study were removed from the statements. The examples shown are not an exhaustive list, but were chosen to illustrate the different phrasing of statements belonging to the different categories. It is worth noting that the statements presented correspond to the objective and conclusion subheading of the abstract. When assessing the abstracts, we identified that these were the subheadings under which the information to classify the abstract was mainly found. Other subheadings like Design, Setting and Participants were not as relevant for this purpose.

To further illustrate how statements in these two sections can be misleading, we tabulated a few examples in a 2 by 2 table showing mismatches between what was reported in the objectives and conclusion resulting in the paper being categorised as either 'consistently (not) causal' or 'inconsistent' (Table 2).

Assessment of submitted versions

After evaluating the first submitted version of the 12 abstracts classified as 'inconsistent', we classified 11/12 (92%) as also inconsistent on submission. There was only one study where the submitted version described a different type of association. In this case, the conclusion of both the submitted and published versions was rather conservative by stating that the intervention was "independently

For peer review only

associated" with the outcome. The submission expressed a causal objective, stating the aim of evaluating the "impact" of a particular intervention with corresponding methods: providing adjusted estimated effects and including sensitivity analysis using propensity score matching. However, in the published version the term "impact" was replaced by "association" making the abstract less clear about a causal aim because both the objectives and the conclusion described an association but the authors still provided adjusted hazard ratios and resorted to propensity score matching.

Assessment of full text

Looking at the methods used in the papers classified as 'inconsistent', we found that 11 of the 12 provided adjusted estimates. Most of the studies (8/12, 67%) used outcome regression models, mainly Cox proportional hazard models, or (propensity score) matching (3/12, 25%). Table 3 presents statements found in both the published abstract and full text of each of these papers regarding the method used and considerations suggesting a causal aim.

Discussion

Statement of principal findings

We found that the majority of the published research paper abstracts of observational studies had a consistent use of causal language. Still 20% of them contained inconsistent messages on the causal nature of the key "effect". Inconsistencies showed up in two directions: an intentional quest for causality ending in uncriticized non-causal conclusions or carefully phrased mere associations ending with recommendations to act and intervene based on the exposure outcome association.

Beyond the wording in the abstract, readers can learn much about the sought, after interpretation from described statistical methods, and assumptions made explicit in the paper. On a case by case basis, one could then assess whether additional assumptions, e.g. involving 'no-unmeasured confounders', would justify the causal assessment derived from these approaches. Identifying key elements like the ones presented in Table 3 would help to assess if causal inference is possible. If in doubt, a sensitivity analysis may be in order. It seems better to be transparent about the ultimate aim to draw a causal conclusion and to acknowledge to fall short of that, than to generate confusion.

Comparison with other studies

This is not the first study to evaluate the use of causal language in the medical literature. Cofield et al (5) assessed the use of causal language in observational studies in nutrition. However, they reduced the problem to assessing whether authors included causal language or not, as it was deemed inappropriate due to the observational nature of the study. We have made the case that merely avoiding explicit causal terms is not a real solution. Even without them, a causal conclusion is implicit when the take home message encourages interventions based on the presented findings. Avoiding inconsistency is important but equally one should be able to trust that the use of consistent causal language is not in vain. This requires a more in depth look at methods and assumptions validating the casual claims.

Strengths and weaknesses of the study

Accurate abstracts are important. In just a few brief paragraphs, the author summarizes key elements of design, methods and results, and comes to a conclusion. Many readers only read the abstract. However, a powerful abstract opens the door to readers and sets the scene for any study. It serves the

For peer review only

different roles of informing the audience about its main findings while motivating the reader to further explore the full text, all within the constraints of brevity. This demands from authors special attention to ensure that every word in the abstract is required. All of the above makes the assessment of the abstract relevant but also challenging.

Further research is needed to explore how causal claims presented in the abstract are supported by the full article, which entails assessing the methods used and evaluating whether the underlying assumptions were met (18). The ultimate conclusion should not simply label a study as black or white in causal terms. In the present study we used a convenient limited number of classifications for short statements. In practice a continuous degree of confidence in a potential causal relationship is likely to emerge based on observed association.

We are aware that by limiting our assessment to the abstract, we may have missed the discussion of the extent to which the underlying assumptions that enable causal inference were met. Indeed, when there was a clear causal aim but the authors considered that these assumptions were not fulfilled, they may have decided that a causal claim was inappropriate and phrased their conclusion in terms of association rather than causation. If this is the case, the apparent inconsistency found in the abstract would no longer hold. On the contrary, any undue causal claims can be viewed as a form of spin (19, 20).

Conclusions and policy implications

As observational data resources abound, methods for causal inference from observational data have surged in tandem with the call for real world evidence. The new opportunities bring new challenges and the responsibility for clear and well supported statements on the evidence. In this spirit and motivated by novel guidelines as proposed by ICH9 and FDA, Miguel Hernan and collaborators have embarked on a project entitled "Developing Guidelines for the Analysis of Randomized Controlled Trials in Real-World Settings"(21). The importance of such initiatives, supports a shift towards being explicit and discussing assumptions underlying causal methods that allow for causal interpretations in context, with or without an RCT (13). In the meantime, uncritical ambiguous phrasing in observational studies remains prevalent (14). Those searching for the best possible evidence supporting future treatment decisions, are best served by transparent reports on observational studies.

Faced with uncertainty when concluding on the nature of the observed exposure outcome relationship, a justifiable balance between the type I and II error rate is a natural guide for action. The cost of errors must be weighed in context, for instance as in clinical trials emphasizing control of the type I error to avoid introducing new unhelpful drugs at a potentially large cost. Alternative weights are typical in screening programs where false positives will be caught in follow-up examinations, but false negatives are lost forever. In a crisis, such as the current COVID-19 pandemic, we must act before long term randomised trials have materialised. It becomes undeniably important to learn as much as we can from observational data, be aware of the types of risk when acting or not, as displayed in the schematic Table 4.

A prerequisite for good causal language practice includes awareness of which language implies a causal statement and which does not. To support correct phrasing and raise awareness, we have compiled a short list of words and expressions with dedicated (non) causal meaning (Box). The list draws on phrases found in our study and in the references cited, particularly Hernan et al (10) and Thapa et al

For peer review only

(6). We consider that a definition of causal language that is generally recognized by the research community is needed (22, 23).

Words like “effect”, ”impact”, “determinant of”…, inevitably point in the causal direction and their use should come with the requirement of at least stating and ideally critically evaluating the necessary assumptions (6). Uncertainty on the causal nature of the conclusion should tone down any suggestion for intervening on the studied exposure. Specifying the corresponding level of evidence rather than hiding the ultimate causal aim of a study is what we recommend (19), while acknowledging a margin of error in any empirical study (20).

Box. Examples of words and study elements that could point to causality or otherwise.

Words expressing a causal relationship	• Affect<br>• Attributable<br>• Benefit<br>• Cause<br>• Contribute<br>• Determinant<br>• Efficacy<br>• Impact<br>• Improve<br>• Leads to<br>• Responsible for<br>• Results in<br>• Safety
Words that could suggest causality in a given context	• Effect<br>• Induce<br>• Higher (lower) probability<br>• Modify<br>• Risk (factor)
Specific expressions avoiding suggestions of casual effects	• Association<br>• Correlation<br>• Less (more) likely link<br>• Predict<br>• Pattern
Key aspects suggesting causal aim	• Adjusting for confounders<br>• Discussing ‘unmeasured confounders’ assumption<br>• Suggesting/recommending intervention<br>• Using instrumental variables<br>• Propensity score adjustment

For peer review only

In summary, we have found that causal messages are embedded in studies otherwise carefully phrased in terms of association. Further guidance for authors appears needed on what constitutes a causal statement, similar to the one published by Lederer et al (13) for Respiratory, Sleep and Critical Care

Journals. We look forward to similar guidance for other disease groups. From the screened BMJ abstracts, we provided a list of expressions with clear interpretation which may inspire a useful more comprehensive compendium. We argue that such awareness and special attention amongst authors and reviewers would serve our communication on the best available evidence for conceived interventions.

For peer review only

Contributorship statement

- Conceptualization: COP, LB, AD, SS, EG
- Methodology: COP, EG
- Data curation: COP, LB
- Formal analysis: COP, LB, EG
- Writing original draft: COP
- Writing-review and editing: LB, SS, AD, EG
- Approved final version: COP, LB, AD, SS, EG

Competing interests declaration

SS is a full-time employee of the BMJ Publishing Group. No other conflicts of interest to declare.

Funding

This project has received funding from the European Union's Horizon 2020 research and innovation programme under the Marie Sklodowska-Curie grant agreement No 676207

Patient and public involvement

Patients were not involved in the design, analysis or interpretation of the study. Patients were not participants in this study; it was a methodological study (research on research). Patients' opinions of causal statements and the use of ambiguous language in research papers is important and further work in this area partnered by patients is important.

Ethics Approval

This study used routinely collected data. When authors and reviewers submit manuscripts and reviews to The BMJ, they are notified that their paper or review may be entered into research projects for quality improvement purposes.

Transparency declaration

COP confirms that this manuscript is an honest, accurate, and transparent account of the study being reported and that no important aspects of the study have been omitted.

Data sharing statement

The reviews and published versions of the papers included in the study are publicly available at BMJ.com. No further data will be made available as it is confidential submission data.

References

1. Begg MD, March D. Cause and association: Missing the forest for the trees. American Journal of Public Health. 2018;108(5):620-.

2. Hernán MA. A definition of causal effect for epidemiological research. Journal of Epidemiology and Community Health. 2004;58(4):265-71.

For peer review only

3. Kunz R, Oxman aD. The unpredictability paradox: review of empirical comparisons of randomised and non-randomised clinical trials. Bmj. 1998;317(October):1185-90.

4. Ruich P. The use of cause-and-effect language in the JAMA Network journals. 2017.

5. Cofield SS, Corona RV, Allison DB. Use of causal language in observational studies of obesity and nutrition. Obesity Facts. 2010;3(6):353-6.

6. Thapa DK, Visentin DC, Hunt GE, Watson R, Cleary M. Being honest with causal language in writing for publication. Journal of Advanced Nursing. 2020;76(6):1285-8.

7. Pinsky PF, Miller A, Kramer BS, Church T, Reding D, Prorok P, et al. Evidence of a healthy volunteer effect in the prostate, lung, colorectal, and ovarian cancer screening trial. American journal of epidemiology. 2007;165:874-81.

8. Food Drug Administration (FDA), Permutt T, Scott J. E9(R1) Statistical Principles for Clinical Trials: Addendum: Estimands and Sensitivity Analysis in Clinical Trials. 2017;9.

9. European Medicine Agency (EMA). ICH E9 (R1) addendum on estimands and sensitivity analysis in clinical trials to the guideline on statistical principles for clinical trials 2020 17 February 2020

10. Hernan MA. The C-Word : Scientific Euphemisms Do Not Improve Causal Inference From Observational Data. 2018;108(5):616-9.

11. Glymour MM, Hamad R. Causal thinking as a critical tool for eliminating social inequalities in health. American Journal of Public Health. 2018;108(5):623-.

12. Green MJ. Calculating Versus Estimating Causal Effects. American Journal of Public Health. 2018;108(8):e4-e5.

13. Hernán M, Robins J. Causal Inference: What If: Boca Raton: Chapman & Hall/CRC; 2020.

14. Goetghebeur E, Cessie Sl, De Stavola B, Moodie E, Waernbaum I. Formulating causal questions and principled statistical answers. 2019.

15. Haber N, Smith ER, Moscoe E, Andrews K, Audy R, Bell W, et al. Causal language and strength of inference in academic and media articles shared in social media (CLAIMS): A systematic review. PLoS ONE. 2018;13(5):1-21.

16. Lederer DJ, Bell SC, Branson RD, Chalmers JD, Marshall R, Maslove DM, et al. Control of Confounding and Reporting of Results in Causal Inference Studies. Guidance for Authors from Editors of Respiratory, Sleep, and Critical Care Journals. Annals of the American Thoracic Society. 2019;16(1):22-8.

17. Harhay MO, Au DH, Dell SD, Gould MK, Redline S, Ryerson CJ, et al. Methodologic Guidance and Expectations for the Development and Reporting of Prediction Models and Causal Inference Studies. Annals of the American Thoracic Society. 2020;17(6):679-82.

18. Hernán MA, Robins JM. Estimating causal effects from epidemiological data. Journal of Epidemiology and Community Health. 2006;60(7):578-86.

19. Boutron I. Reporting and Interpretation of Randomized Controlled Trials With Statistically Nonsignificant Results for Primary Outcomes. JAMA. 2010;303(20):2058-.

For peer review only

20. Ghannad M, Olsen M, Boutron I, Bossuyt PM. A systematic review finds that spin or interpretation bias is abundant in evaluations of ovarian cancer biomarkers. Journal of Clinical Epidemiology. 2019;116:9-17.

21. Developing Guidelines for the Analysis of Randomized Controlled Trials in Real-World Settings [Available from: https://www.pcori.org/research-results/2015/developing-guidelines-analysis-randomized-controlled-trials-real-world.

22. Doan S, Yang EW, Tilak SS, Li PW, Zisook DS, Torii M. Extracting health-related causality from twitter messages using natural language processing. BMC Medical Informatics and Decision Making. 2019;19(Suppl 3).

23. Chiolero A. Causality in public health: One word is not enough. American Journal of Public Health. 2019;109(10):1319-20.

Figure Legends

Figure 1. Adapted Prisma flow diagram showing study selection and main study findings.

For peer review only

Tables

Table 1. Examples of statements found in the objectives and conclusions of abstracts of observational studies published in The BMJ in 2018 and their corresponding assigned category.

Assigned category	Objective	Conclusion	Comment
Consistently causal	"...assess the effectiveness of..."	"Little evidence was found of a direct impact of..."	When discussing associations, words like effect, contribution or role are similar to cause and then (direct) impact and effect will be their consequence
	"To determine the effect of ... in ..."	"...has led to risk..."	
	"To describe the contributions of..."	"... an important role in ..."	
	"To evaluate the impact of ..."	"...impacts are..."	
	"To investigate whether improving adherence to ...."	"...the beneficial effect of improved..."	Evaluates taking an action 'improving adherence' and concludes that the effect is beneficial
	"...benefit of ... in reducing ... risk"	"... is an overlooked risk factor for ..."	Evaluates how a given intervention can reduce the risk of an outcome and then labels it as an 'overlooked risk factor'
	"To determine outcomes and safety of..."	"... is at least as effective and safe as ..."	Evaluates and determines that a certain intervention is as safe as the comparator

For peer review only

	"to quantitatively decompose this joint association to ... only, to ... only, and to their interaction."	"...excess risk of...These findings suggest that most cases of ... could be prevented by ..."	Suggests interest in direct and indirect effect i.e. mediation analysis, and concludes consequently
Consistently not causal (Associations)	"...is associated with ...compared with..."	"...is associated with ...compared with..."	Describes associations without labelling them as causal or prediction
	"To describe trends in..."	"...rates were high during the study period of ... with the highest rates in ... versus ..."	Limits to describe frequency
	"To assess how often ..."	"One in ... adults ... were ..."	
	"To examine the association between..."	"...could increase ... confirmation of these findings are warranted, preferably in an intervention setting."	Suggests further research to determine the nature of the association
	"...compared with...is associated with..."	"Additional studies, with long term follow-up, are needed to investigate the effects of..."	
Consistently not causal (Prediction)	"To develop and validate a set of practical prediction tools that reliably estimate the outcome of..."	"...prediction models reliably estimate the outcome..."	Describes developing and validating prediction models

For peer review only

	“To prospectively validate the ... algorithm to ...”	“...accurately classified...”	
Inconsistent	“...evaluate safety of...”	“...associated with...”	Phrasing the objective as causal and limiting to describing an association in the conclusion
	“...analyse the effect of...”		
	“...critical determinant...”		
	“...association with...”	“...is safe...”	Phrasing the objective as just to explore an association and presenting a causal claim in the conclusion
		“... had no substantial effect on long term survival...”	
		“... was determined by... may be largely explained by...”	
		“... was found to be the safest drug, with reduced risks of...”	
		“These results emphasise the benefit of...”	
	“...association with...”	“...tackling all these risk factors might substantially... “	Phrasing the objective and conclusion as if just to assess an

For peer review only

		"...Targeting ... prevention strategies among these patients should be considered."	association but then suggesting to take action given the findings
		"Systematically addressing ... may be an important public health strategy to reduce the incidence of"	
		"...present findings encourage the downward revision of such guidelines ..."	

For peer review only

Table 2. Examples of (mis)matching causal and non-casual statements found respectively in the objectives and conclusions of abstracts of observational studies published in The BMJ in 2018.

		Conclusion	
		Causal	Not causal
Objective	Causal	Consistent<br>"...assess the effectiveness of..."and "Little evidence was found of a direct impact of..."<br>"...benefit of ... in reducing ... risk" and "... is an overlooked risk factor for ..."	Inconsistent<br>"...evaluate safety of..." and "...associated with..."<br>"...analyse the effect of..." and "...associated with..."<br>"...critical determinant..." and "...associated with..."
	Not causal	Inconsistent<br>"...association with..." and "...is safe..."<br>"...association with..." and "... had no substantial effect on long term survival..."<br>"...association with..." and "...tackling all these risk factors might substantially... "<br>"...association with..." and "Systematically addressing ... may be an important public health strategy to reduce the incidence of"	Consistent<br>"To describe trends in..." and "...rates were high during the study period of ... with the highest rates in ... versus ..."<br>"To assess how often ..." and "One in ... adults ... were ..."<br>"To develop and validate a set of practical prediction tools that reliably estimate the outcome of..." and "...prediction models reliably estimate the outcome..."

For peer review only

Table 3. Statements in each of the 12 published observational studies classified as "inconsistent"

Abstract		Full text			
Objective	Conclusion	Method	Confounder adjustment	Estimates provided	Considerations
"To evaluate the relation between […] and development of […]"	" […] was associated with an increased risk of […] that was mediated by […]. Systematically addressing […] may be an important public health strategy to reduce the incidence of […] among patients wih […]."	"We calculated the hazard ratios for the relation of […] to the risk of MRSA using Cox proportional hazard models. […] We also calculated the absolute risk difference. […] We performed mediation analyses to examine the extent to which the effect of […] on the risk of […] was through […]. Using marginal structural models we then estimated the natural direct effect […] and the natural indirect effect […] while adjusting for the same confounding variables"	"We performed a matched cohort study […]matched on age (one year either way), sex, and study entry time (within one year either way). Such comparators were chosen to further ensure the comparability […] In the multivariable Cox model we adjusted for […]."	"The matched and multivariable adjusted hazard ratios for patients with […] were 1.69 (1.51 to 1.90) for […] and 1.26 (1.12 to 1.40) for […]."	"Our GP practice based dataset could have missed the detection of some inpatient cases of […]; however, these potential non-differential misclassifications would have biased our results towards the null, rendering our findings conservative"
"To quantify the effects of varying […]"	"[…] is associated with a large increase in […] among […] patients. The data from this study	"For adjusted analysis of time until […] we used Cox proportional hazards models."	"Adjusted models included […]"	"Each additional […] increased the rate of […] by 70.7% (95% confidence interval	"To determine the extent to which strong unobserved confounding might

For peer review only

For peer review only - http://bmjopen.bmj.com/site/about/guidelines.xhtml

	suggest that […] rather than […] is more strongly associated with […]"			54.6% to 88.4%) before adjustment and increased the hazard of […] by 44.0% (40.8% to 47.2%, P<0.001) after adjusting for covariates."	explain the observed association, we included this synthetic confounder in a Cox model. […] As part of a sensitivity analysis, we constructed models that removed potential confounders. […]"
"To evaluate the long term association between […]"	"Widespread utilisation of […] may be contributing to long term increased risk of […]. The potential for […] should be considered when[…]."	"We conducted Poisson regression analyses using person years as observations."	"We included several variables as known confounders or effect modifiers in the relation between. […]The final fully adjusted model adjusted for […]"	"After adjustment for covariates, the rate ratio was […] indicating that during the entire period of follow-up the risk of […] was 21% higher during […] than at other times."	"The registered active […] population is generally representative of the UK population in terms of age, sex, and regional distribution"
"To investigate the association of […]"	"The shape of the association between […] and […] was determined by […].This finding suggests that the […] may be largely explained by […]"	"We used Cox proportional hazards models to estimate hazard ratios and 95% confidence intervals. We stratified the analysis by age in months and calendar year of the questionnaire cycle."	"For the main analysis, we used […] measured at baseline to minimize the effect of underlying diseases on mortality […] In multivariable models, we adjusted for potential confounders including […]"	"A multivariable adjusted model showed a positive association between […] and all cause mortality, whereas […] showed a U shaped association with all cause mortality. In a mutually adjusted model including both	"Our findings remained robust in several sensitivity analyses […] we cannot entirely rule out the possibility of unmeasured or unknown confounding factors that may account for

				[…] and […] we consistently observed a strong positive association between […] and all cause mortality.”	the associations observed in this study.”
“ To examine the associations of […]”	“This association could be explained by the finding that […] These results emphasise the importance of revisiting […] or establishing specific guidelines for management among […]”	“We performed Cox models with penalised splines”	“In final Cox models with penalised splines, we made adjustments for: […]”	“[A]fter adjustment for confounding factors, the U shaped association with […]”	
“ To estimate long term survival, health, and educational/social functioning in patients with […]”	“[…] had no substantial effect on […]”	“We calculated mortality rate ratios and incidence rate ratios as measures of relative risk.”	“For each […] patient, we used the Danish Civil Registration System and the DNPR to identify all Danish residents with the same sex and date of birth as the patient who had not tested positive […] and who met the study’s inclusion and exclusion criteria […]. From this population, we extracted 10 people at random for each	Patients and members of the comparison cohort were well matched with respect to […] Mortality was not higher among patients in the […] cohort”	

For peer review only

			patient. People in the population comparison cohort were assigned the same date of study inclusion as […] patients to whom they were matched.”		
“To compare the risk of […]”	“ Although residual confounding cannot be excluded, this finding deserves consideration when […] is used for […]”	“We estimated the crude hazard ratio of […] using Cox proportional hazard regression, and the adjusted hazard ratio was obtained using propensity score matching”	“ We identified potential confounders that were plausibly associated with both […]based on clinical knowledge […] In the context of this study, the propensity score is the probability of receiving […] as opposed to […], given the baseline characteristics. Patients who received […] were matched to patients who […] using a 1:1 nearest neighbor matching algorithm with a caliper of 0.2 of the standard deviation of the propensity score on the logit scale. Covariate balance between the two groups was	“The crude hazard ratio of death in the unmatched cohort was 1.51 (95% confidence interval 1.22 to 1.85) and the adjusted hazard ratio in the matched cohort was 1.50 (1.14 to 1.96)”	“Comparison of the baseline characteristics in the unmatched cohort provided little evidence of confounding […] it is unlikely that a few additional unmeasured variables can explain a 50% increase in the risk independent of all other confounder and proxies of confounders that are adjusted for in our study.”

For peer review only

			assessed after matching, and we considered an absolute standardized difference less than 0.1 as evidence of balance”		
“To determine whether […] is associated with […]”	“[…] was independently associated With […]”	“We fitted both a mixed effect logistic regression model (in which the outcome was defined as dichotomous […] and the Prentice, Williams, and Peterson (PWP) model”	“We examined the relation between […] and […] adjusted for …”	“The rate of distinct criteria met per year increased by 24% if a patient had been admitted to hospital (hazard ratio 1.24, 95% confidence interval 1.20 to 1.28) when controlled for the other covariates”	“We did a sensitivity analysis using propensity score matching to assess whether the association between […] and […] could be due to unmeasured confounders […] Although we adjusted for a range of characteristics of patients, as with any observational study potential exists for unmeasured confounding, which may partly or fully explain the relation between […]”
“To investigate associations between […]and to analyse the effect of changes […]”	“Risks of […] are inversely associated with […]”	“We used multivariable Cox regression analysis to compare the rates of […] and […].“	“Confounders included in the final models were based on the literature or statistical significance	“Compared with […], […] had increased hazard ratios of […]”	“We believe that our findings are widely applicable and provide justification

For peer review only

			(P<0.10). The full model included […]”		for […] and continuing […]”
“To estimate the rates of […]”	“In cases of […], approximately […] will become […], of which a third will have […].”	“ We present denominators where data for the secondary outcome are missing. We defined the population attributable fraction as (Re–Run)/Re=(RR–1)/RR, calculated using Stata. To test the robustness of our findings, we did a sensitivity analysis.”	“We compared the demographic and clinical variables of […]. We used the binomial Wilson score to calculate confidence intervals of single proportions and the Pearson exact method to calculate confidence intervals of risk ratios and medians.”	“[…] had a higher risk of […]The population attributable fraction of […] was 47% for […] and 61% for […]”	“ Considering these results when counselling potentially exposed […] seems reasonable”
To perform an expedited assessment of […] risk associated with exposure to […]”.	“The results do not imply a markedly increased short term overall risk of […] in […].”	“We used Cox regression to estimate the hazard ratio with 95% confidence intervals for […] associated with […], both for ever use and for the predefined categories of cumulative use”	“Analyses were, however, performed as crude comparisons adjusted only for […] as well as adjusted for […] and the potential confounding factors.”	“Overall, exposure to […] showed no association with […] compared with exposure to […] (adjusted hazard ratio 1.09, 95% confidence interval 0.85 to 1.41) and no evidence of a dose-response relation”	“This ensured that the estimates were not affected by immortal time bias [..] As all comparisons were performed within users of […], the exposure to […] can reasonably be expected to be a random event, and confounding is thus expected to be limited.”

For peer review only

“To investigate the risks of […] in […]”	“No increased risk of […] was detected in […], but increased risks of […] were found in this study. Our results suggest that […] risks could be due to […], rather than […].”	“To calculate expected […], we multiplied the person years at risk by corresponding national incidence rates (by 5 year age band and individual calendar year) for the general female population of England and Wales. Standardised incidence ratios were calculated by the comparison of observed values with expected values.”	“We obtained data relating to potential confounding factors such as […]”	“There was no overall increased risk of […] (2578 observed v 2641.2 expected […]; standardised incidence ratio 0.98 (95% confidence interval 0.94 to 1.01); absolute excess risk [illegible] cases per 100 000 person years (95% confidence interval [illegible] to 1.8); table 2)”.	“Given previous inconsistent results, small study size, and lack of information on potential confounders, we undertook a population based linkage study in […]”

For peer review only

Table 4. Impact of the errors of causal effect assignment.

		True nature of the main exposure effect	
		Causal	Not causal
Reported nature of the studied exposure effect	Causal	A true causal effect has been discovered. Recommendation to act on this should be considered. Language in the context of a study intended for causal inference.	Type I error: there is no causal effect, but it is claimed.<br>Causal language used or suggestion to take action made when the purpose was to find associations
	Not causal	Type II error: hiding the true causal objective by avoiding use of causal language	No causal language when the objective is prediction or to explore associations

For peer review only

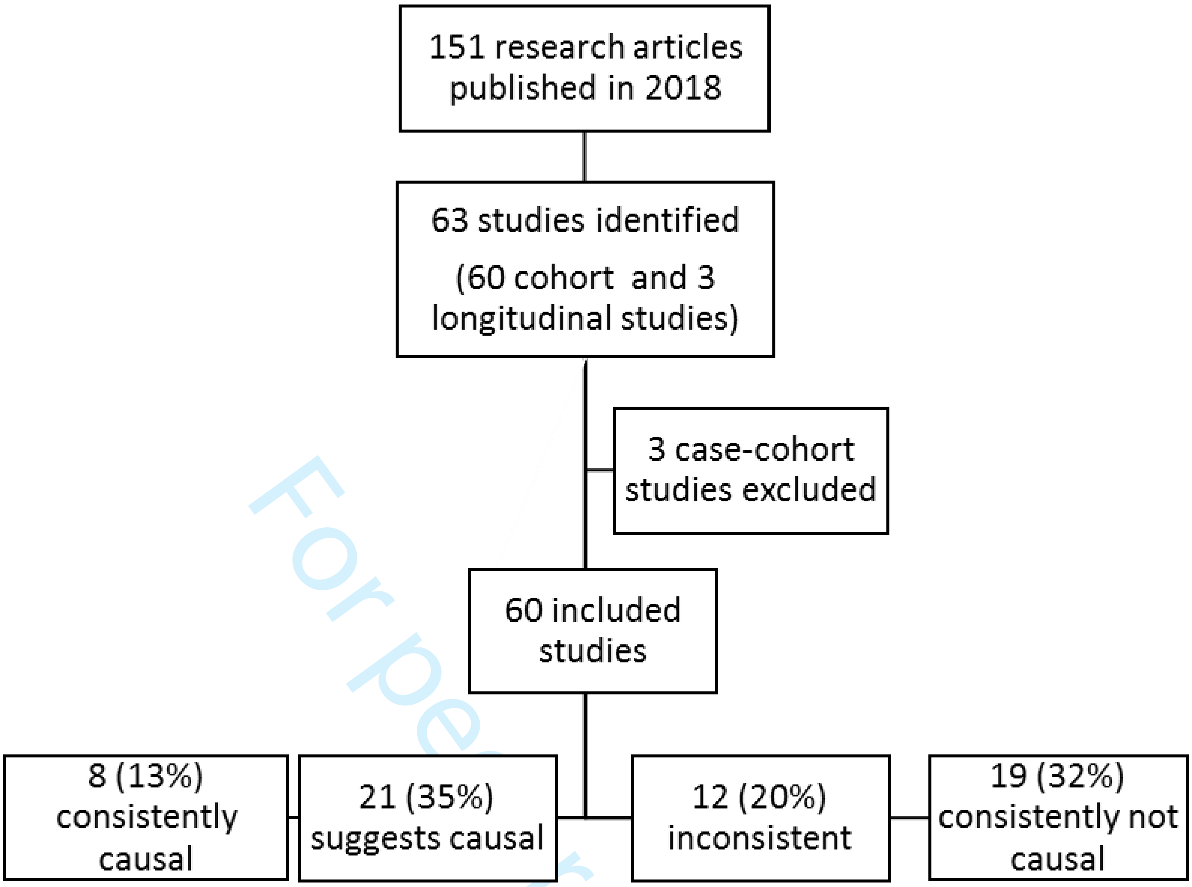

For peer review only

BMJ Open

Use of causal claims in observational studies: a research on research study

Journal:	*BMJ Open*
Manuscript ID	bmjopen-2020-043339.R1
Article Type:	Original research
Date Submitted by the Author:	16-Dec-2020
Complete List of Authors:	Olarte Parra, Camila; Ghent University, Applied Mathematics, Computer Science and Statistics; University of Amsterdam, Clinical Epidemiology, Biostatistics and Bioinformatics<br>Bertizzolo, Lorenzo; INSERM, U 1153, Equipe Methods; University of Amsterdam, Clinical Epidemiology, Biostatistics and Bioinformatics<br>Schroter, Sara; BMJ, Editorial<br>Dechartres, Agnes; Hopitaux Universitaires Pitie Salpetriere-Charles Foix, Departement biostatistique sante publique et information medicale<br>Goetghebeur, Els; Ghent University, Applied Mathematics, Computer Science and Statistics
**Primary Subject Heading**:	Research methods
Secondary Subject Heading:	Epidemiology
Keywords:	STATISTICS & RESEARCH METHODS, EPIDEMIOLOGY, EDUCATION & TRAINING (see Medical Education & Training)

SCHOLARONE™
Manuscripts

I, the Submitting Author has the right to grant and does grant on behalf of all authors of the Work (as defined in the below author licence), an exclusive licence and/or a non-exclusive licence for contributions from authors who are: i) UK Crown employees; ii) where BMJ has agreed a CC-BY licence shall apply, and/or iii) in accordance with the terms applicable for US Federal Government officers or employees acting as part of their official duties; on a worldwide, perpetual, irrevocable, royalty-free basis to BMJ Publishing Group Ltd ("BMJ") its licensees and where the relevant Journal is co-owned by BMJ to the co-owners of the Journal, to publish the Work in this journal and any other BMJ products and to exploit all rights, as set out in our licence.

The Submitting Author accepts and understands that any supply made under these terms is made by BMJ to the Submitting Author unless you are acting as an employee on behalf of your employer or a postgraduate student of an affiliated institution which is paying any applicable article publishing charge ("APC") for Open Access articles. Where the Submitting Author wishes to make the Work available on an Open Access basis (and intends to pay the relevant APC), the terms of reuse of such Open Access shall be governed by a Creative Commons licence – details of these licences and which Creative Commons licence will apply to this Work are set out in our licence referred to above.

Other than as permitted in any relevant BMJ Author's Self Archiving Policies, I confirm this Work has not been accepted for publication elsewhere, is not being considered for publication elsewhere and does not duplicate material already published. I confirm all authors consent to publication of this Work and authorise the granting of this licence.

For peer review only

Use of causal claims in observational studies: a research on research study

Camila Olarte Parra[1,2], Lorenzo Bertizzolo[2,3], Sara Schroter[4], Agnès Dechartres[5], Els Goetghebeur[1]

1. Department of Applied Mathematics, Computer Science and Statistics, Ghent University, Ghent, Belgium

2. Department of Clinical Epidemiology, Biostatistics and Bioinformatics, Academisch Medisch Centrum, Amsterdam, The Netherlands

3. INSERM, U1153 Epidemiology and Biostatistics Sorbonne Paris Cité Research Center (CRESS), Methods of therapeutic evaluation of chronic diseases Team (METHODS), Paris, F-75004 France; Paris Descartes University, Sorbonne Paris Cité, France.

4. Editorial, The BMJ, London, United Kingdom

5. Sorbonne Université, Institut National de la Santé et de la Recherche Médicale (INSERM), Institut Pierre Louis d'Epidémiologie et de Santé Publique, AP-HP. Sorbonne Université, Hôpital Pitié Salpêtrière, Département de Santé Publique, F75013, Paris, France

Correspondence to: Camila Olarte Parra
Department of Applied Mathematics, Computer Science and Statistics
Ghent University, Ghent, Belgium
Campus Sterre, S9, Krijgslaan 281, 9000 Ghent, Belgium
Email: camila.olarteparra@ugent.be, colartep@gmail.com

For peer review only

Abstract

Objective: To evaluate the consistency of causal statements in observational studies published in The BMJ.

Design: Research on research study.

Data source: Cohort and other longitudinal studies describing an exposure-outcome relationship published in The BMJ in 2018. We also had access to the submitted papers and reviewer reports.

Main outcome measures: Proportion of published research papers with 'inconsistent' use of causal language. Papers where language was consistently causal or non-causal were classified as 'consistently causal' or 'consistently not causal', respectively. For the 'inconsistent' papers, we then compared the published and submitted version.

Results: Of 151 published research papers, 60 described eligible studies. Of these 60, we classified the causal language used as 'consistently causal' (48%), 'inconsistent' (20%) and 'consistently not causal'(32%). Eleven out of 12 (92) of the 'Inconsistent' papers were already inconsistent on submission. The inconsistencies found in both submitted and published versions was mainly due to mismatches between objectives and conclusions. One section might be carefully phrased in terms of association while the other presented causal language. When identifying only an association, some authors jumped to recommending acting on the findings as if motivated by the evidence presented.

Conclusion: Further guidance is necessary for authors on what constitutes a causal statement and how to justify or discuss assumptions involved. Based on screening these papers, we provide a list of expressions beyond the obvious 'cause' word which may inspire a useful more comprehensive compendium on causal language.

Strengths and limitations of this study

- We present examples of ambiguous causal statements in published observational studies in a high impact journal
- We focused on the abstract where clear messages are especially important, as many readers just read the abstract of a study
- The focus on the causal language may miss further discussion on the validity of underlying assumptions justifying causal inference in the setting studied.
- The prevalence and nature of the problems found is a call for better instruction on and consideration of causal language throughout the editorial process in clinical and epidemiological research.
- We provide a list of words and study elements that could point in the direction of causality or otherwise, which may inspire a more comprehensive compendium.

For peer review only

Introduction

Many researchers remain tempted to draw causal conclusions from observational data despite acknowledging that mere association is not causation because causal inference is the ultimate goal of most clinical and public health research (1, 2). Gold-standard answers are typically sought through randomized controlled trials (RCTs). The unique ability of RCTs to avoid confounding bias (3) has led to demands that empirical research must be drawn from randomized studies to justify causal statements (4-6). RCTs are mainly used to assess the effect of a treatment or intervention but are not easily adapted to evaluate prognostic or risk factors rather than interventions.

There are however good reasons to look beyond RCTs for evidence on treatment effects. In many settings, RCTs are not feasible, ethical or timely and thus observational data are all that is available for some time, as in the recent COVID-19 crisis. Furthermore, observational studies typically involve broader real-world contexts than RCTs, where the costs and risks of experimentation suggest studying high risk patients without major comorbidities (7). This selection challenges generalization to the target population. Highly selected populations with a usually short follow-up, render RCTs inappropriate to evaluate (long-term) unintended side effects. Trials further suffer from treatment non-compliance which complicates analysis, as treatment-specific populations lose the benefit of randomization. Recent ICH E9 guidelines therefore emphasize the importance of causal estimands beyond intention-to-treat, such as per-protocol and as-treated analysis (8, 9).

Deliberately avoiding causal statements on a hoped-for causal answer brings ambiguity and contrived reporting (10, 11). Instead, authors should openly discuss the likely distance in meaning and magnitude between the data based measure they are able to estimate and the desired targeted causal effect. Arguments would consider study design with additional assumptions in context (12). Owing to decades of progress in statistical science (involving potential outcomes, directed acyclic graphs, propensity scores and more) (13), this allows for results, often unreachable by randomized trials, with a justified causal interpretation (14).

In 2010, Cofield et al (5) assessed the use of causal language in observational studies in nutrition but deemed causal language inappropriate for all observational studies. In 2017, Adams et al. (15) also considered that only RCTs allowed for causal inference, in their study assessing how people understand causal expressions in the news. From a different angle, Haber et al (16) examined whether the tone and strength of causal claims made in a given paper matched the language describing the findings in social media. Not surprisingly they found stronger causal statements in the media in half of the cases, emphasizing the importance of clear scientific messages.

To promote this, Lederer et al (17) recently published a guide for authors and editors on how to report causal studies in Respiratory, Sleep and Critical Care Journals. Rather than circumventing the problem by asking to avoid causal language, they provide key elements that ensure valid causal claims (18). Besides briefly explaining causal inference, they provide a definition of a confounder, outline how to identify confounding through so-called directed acyclic graphs and discuss how p-values are often misinterpreted and how their value does not reflect the magnitude, direction or clinical importance of a given association. All these elements empower their target audience to critically assess observational studies.

To find out whether and how statements in study reports present confusing use of causal language (or lack thereof), we examined research papers concerned with exposures and outcomes published in The

For peer review only

For peer review only - http://bmjopen.bmj.com/site/about/guidelines.xhtml

BMJ in 2018. Our focus was on the causal message The BMJ readers receive from these papers, particularly from the abstract. We evaluate the consistency of causal statements in the published abstracts of observational studies, whether this consistency was a reflection of the full text and if any a priori changes had been made as a result of the peer review process.

Methods

Sampling and inclusion criteria

COP identified all original research articles published in The BMJ in 2018 described as either cohort or longitudinal studies in the study design section of the abstract. The eligible studies were identified by statements in this section of the abstract such as “cohort”, “longitudinal” or “registry-based”. Those identified as “observational” were included if they suggested a period of follow-up rather than being cross-sectional. Articles described as case-cohorts were excluded as their interpretation and analysis differs from other studies with follow-up assessing the exposure-outcome relationship.

Assessment of published abstracts

Two reviewers (COP, LB) independently screened the published abstracts of the eligible papers. For the text included under each of the subheadings in the abstract (objective, design, setting, participants, outcome, results, conclusion), the reviewers assessed whether there was an (implicit) causal (cl)aim using a yes/no/unclear response. After assessing each separate subheading, each reviewer then gave an overall assessment of the main claims in the paper’s abstract as either ‘consistently causal’, ‘inconsistent’ or ‘consistently not causal’. After the independent assessments, the overall rating of the abstract was compared between both reviewers; where there was disagreement, a third reviewer (EG) was consulted and a consensus reached.

Assessment of published full text

We further evaluated the full published text of all eligible papers to identify the statistical methods applied and any further causal claims. In particular, we looked for statements that would support or undermine a causal aim, including confounding adjustment, discussing residual confounding, exchangeability and issues of transportability. We randomly divided the papers between the two reviewers (COP, LB) for this assessment. For each paper, we extracted statements where authors described the statistical method and method for confounding adjustment, if any. We then extracted the sentences summarizing the results and conclusions to highlight any causal claims.

Assessment of initially submitted abstract version

As the focus of this paper to highlight ambiguous use of causal language, we further assessed those articles judged as ‘inconsistent’ to see if there were changes introduced to the manuscript between submission and publication, leading to this inconsistent use of causal language. For this subset, we obtained the submitted version of the manuscripts and the associated peer reviewers’ comments from The BMJ’s manuscript tracking system. We then compared the published version with the first submitted version of the abstract to identify whether the same wording related to causal claims appeared in the submitted version and whether changes occurred as a result of comments from peer reviewers and editors, as indicated in the corresponding peer review reports.

The same reviewers (COP, LB) independently evaluated the submitted versions of the abstracts. The reviewers assessed whether the content under each subheading of the submitted abstract differed

For peer review only

from the published version. Where there were discrepancies between versions, each reviewer indicated the presence of a causal claim as yes/no/unclear for each abstract subheading (title, objective, design, setting, participants, outcome, results, conclusion) and made an overall assessment of the submitted abstract as either 'consistently causal', 'inconsistent' or 'consistently not causal'. As before, the assessments were compared and, in cases of disagreement, a third reviewer (EG) was consulted and consensus reached.

Ethics and consent

We did not seek ethical approval or consent from authors and reviewers for this descriptive quality improvement study. However, when authors and reviewers submit manuscripts and reviews to The BMJ, they are notified that their paper or review may be entered into research projects for quality improvement purposes. COP was given access to The BMJ's data under a confidentiality agreement. We do not report any identifying information. The statements presented in this study belong to published papers available in the public domain.

Patient and public involvement

Patients were not involved in the design, analysis or interpretation of the study. Patients were not participants in this study; it was a methodological study (research on research). Patients' opinions of causal statements and the use of ambiguous language in research papers is important and further work in this area partnered by patients is important.

Results

Assessment of published abstracts

In 2018, 151 research papers were published in The BMJ, of which 60 (40%) were eligible for inclusion in our study. We identified 29 studies (48%) reporting studies using causal language consistently. A further twelve (20%) studies were considered inconsistent mainly because the objective stated the evaluation of an association while the conclusion presented a causal finding (9/12) or the opposite (3/12). Finally, there were papers that described studies aiming for prediction or reporting associations without (implicitly) suggesting that they had a causal nature that were considered consistently not causal (n=19, 32%).

Table 1 shows sample excerpts from the published abstracts that were evaluated. Each row corresponds to statements from the same study. The first column indicates the assigned category, based on the type of association it describes. The last column explains why a given abstract was considered to belong to the assigned category. As the assessment pertains to causal claims in general, the words referring to the particular topic of the corresponding study were removed from the statements. The examples shown are not an exhaustive list, but were chosen to illustrate the different phrasing of statements belonging to the different categories. It is worth noting that the statements presented correspond to the objective and conclusion subheadings of the abstract. When assessing the abstracts, we identified that these were the subheadings under which the information to classify the abstract was mainly found. Other subheadings like Design, Setting and Participants were not as relevant for this purpose, but were also assessed.

To further illustrate how statements in these two sections can be misleading, we tabulated a few examples in a 2 by 2 table showing mismatches between what was reported in the objectives and

For peer review only - http://bmjopen.bmj.com/site/about/guidelines.xhtml

conclusion resulting in the paper being categorised as either 'consistently (not) causal' or 'inconsistent' (Table 2).

Assessment of published full text

Table A in supplementary material presents statements found in both the published abstract and published full text of each of these papers (n=60) regarding the statistical method used and considerations suggesting a causal aim or otherwise. Each row corresponds to a different study. The papers are grouped according to the category to which the corresponding abstract was assigned. The particular causal or non-causal wording is highlighted in bold. A brief description on the consistency of causal language is provided in the last column of table, labelled 'Comment'.

We found that all papers classified as 'consistently causal' based on the abstract, also used causal language and contained causal statements in the full text. This was additionally the case with more than half (11/19) of the abstracts classified as 'consistently not causal' , where even though the abstract was carefully phrased in terms of association, the authors applied causal methods, discussed residual confounding, biological plausibility or a dose-response relationship suggesting a causal aim.

In the previous section, we referred to 3 abstracts that had a clear causal objective but a non-causal conclusion. In the full text of these papers, the authors discussed concerns of residual confounding which explains why they decided to play down the conclusion.

Looking at the methods section in the full text of the abstracts classified as 'inconsistent', we found that 11 of the 12 provided adjusted estimates. Most of the studies (8/12, 67%) used outcome regression models, mainly Cox proportional hazard models, or (propensity score) matching (3/12, 25%).

Assessment of submitted abstract version

Of the 12 published abstracts classified as 'inconsistent', we further classified 11/12 (92%) as also inconsistent on submission. There was only one study where the submitted version of the abstract described a different type of association. In this case, the conclusion of both the submitted and published versions was rather conservative by stating that the intervention was "independently associated" with the outcome. The submitted version expressed a causal objective, stating the aim of evaluating the "impact" of a particular intervention with corresponding methods: providing adjusted estimated effects and including sensitivity analysis using propensity score matching. However, in the published version the term "impact" was replaced by "association" making the abstract less clear about a causal aim because both the abstract's objectives and the conclusion described an association but the authors still provided adjusted hazard ratios and resorted to propensity score matching.

Discussion

Statement of principal findings

We found that the majority (80%) of the published research abstracts reporting on observational studies had a consistent use of causal language in the abstract. Still 20% of abstractscontained inconsistent messages on the causal nature of the key "effect". Inconsistencies showed up in two directions: an intentional quest for causality ending in uncriticized non-causal conclusions or carefully

For peer review only

phrased mere associations ending with recommendations to act and intervene based on the exposure outcome association.

Beyond the wording, readers can learn much about the sought, after interpretation from described statistical methods, and assumptions made explicit in the paper. On a case by case basis, one could then assess whether additional assumptions, e.g. involving 'no-unmeasured confounders', would justify the causal assessment derived from these approaches. Identifying key elements like the ones presented in the Supplementary Material would help to assess if causal inference is possible. If in doubt, a sensitivity analysis may be in order. It seems better to be transparent about the ultimate aim to draw a causal conclusion and to acknowledge to fall short of that, than to generate confusion.

When assessing the full text of the 'consistently causal' papers, we identified that authors often discussed these assumptions and resorted to conducting a sensitivity analysis. This was also the case for those papers that were classified as 'inconsistent' or 'consistently no causal'. In these papers, there was a concern for residual confounding because of the observational nature of the text or due to specific missed confounders. Therefore the abstract's objective avoided suggesting a causal aim instead of being explicit of such concern or limitations in the abstract.

Comparison with other studies

This is not the first study to evaluate the use of causal language in the medical literature. Cofield et al (5) assessed the use of causal language in observational studies in nutrition. However, they focus only on assessing whether authors included causal language or not, as it was deemed inappropriate due to the observational nature of the study. We have made the case that merely avoiding explicit causal terms is not a real solution. Even without them, a causal conclusion is implicit when the take home message encourages interventions based on the presented findings. Avoiding inconsistency is important but equally one should be able to trust that the use of consistent causal language is not in vain. This requires a more in depth look at methods and assumptions validating the causal claims.

Strengths and weaknesses of the study

Accurate abstracts are important. In just a few brief paragraphs, the author summarizes key elements of design, methods and results, and comes to a conclusion. Many readers only read the abstract. However, a powerful abstract opens the door to readers and sets the scene for any study. It serves the different roles of informing the audience about its main findings while motivating the reader to further explore the full text, all within the constraints of brevity. This demands authors to give special attention to ensure that every word in the abstract is required. All of the above makes the assessment of the abstract relevant but also challenging.

Further research is needed to explore how causal claims presented in the abstract and full text are supported by the design and methods applied, which entails assessing the methods used and evaluating whether the underlying assumptions were met (19). The optimal conclusion should not simply label a study as black or white in causal terms. In the present study we used a convenient limited number of classifications for short statements. In practice a continuous degree of confidence in a potential causal relationship is likely to emerge based on observed association.

We are aware that by limiting our assessment to the consistency of causal language, we may have missed the discussion of the extent to which the underlying assumptions that enable causal inference were met. This requires subject-matter knowledge in each particular case (13). Indeed, when there was a clear causal aim but the authors considered that these assumptions were not fulfilled, they may

For peer review only

have decided that a causal claim was inappropriate and phrased their conclusion in terms of association rather than causation. If this is the case, the apparent inconsistency would no longer hold. On the contrary, any undue causal claims can be viewed as a form of spin (20, 21).

Policy implications

As observational data resources abound, methods for causal inference from observational data have surged in tandem with the call for real world evidence. The new opportunities bring new challenges and the responsibility for clear and well supported statements on the evidence. In this spirit and motivated by novel guidelines as proposed by ICH E9 and FDA, Miguel Hernan and collaborators have embarked on a project entitled "Developing Guidelines for the Analysis of Randomized Controlled Trials in Real-World Settings"(22). The importance of such initiatives supports a shift towards being explicit and discussing assumptions underlying causal methods that allow for causal interpretations in context, with or without an RCT (13). In the meantime, uncritical ambiguous phrasing in observational studies remains prevalent (14). Those searching for the best possible evidence supporting future treatment decisions, are best served by transparent reports of observational studies.

Faced with uncertainty when concluding on the nature of the observed exposure outcome relationship, a justifiable balance between the type I and II error rate is a natural guide for action. The cost of errors must be weighed in context, for instance as in clinical trials emphasizing control of the type I error to avoid introducing new unhelpful drugs at a potentially large cost. Alternative weights are typical in screening programs where false positives will be caught in follow-up examinations, but false negatives are lost forever. In a crisis, such as the current COVID-19 pandemic, we must act before long term randomised trials have materialised. It becomes undeniably important to learn as much as we can from observational data, be aware of the types of risk when acting or not, as displayed in Box 1.

Box 1. Impact of the errors of causal effect assignment.

		True nature of the main exposure effect	
		Causal	Not causal
Reported nature of the studied exposure effect	Causal	A true causal effect has been discovered. Recommendation to act on this should be considered. Language in the context of a study intended for causal inference.	Type I error: there is no causal effect, but it is claimed. Causal language used or suggestion to take action made when the purpose/ability was to find associations
	Not causal	Type II error: hiding the true causal objective/result by avoiding use of causal language	No causal language when the objective is prediction or to explore associations

A prerequisite for good causal language practice includes awareness of which language implies a causal statement and which does not. To support correct phrasing and raise awareness, we have compiled a short list of words and expressions with dedicated (non) causal meaning (Box 2). The list draws on phrases found in our study and in the references cited, particularly Hernan et al (10) and Thapa et al (6). This list is a suggestion as a starting point and further study can test and validate it. We consider

For peer review only - http://bmjopen.bmj.com/site/about/guidelines.xhtml

that a definition of causal language that is generally recognized by the research community is needed (23, 24).

Words like “effect”, ”impact”, “determinant of”…, inevitably point in the causal direction and their use should come with the requirement of at least stating and ideally critically evaluating the necessary assumptions (6). Uncertainty on the causal nature of the conclusion should tone down any suggestion for intervening on the studied exposure. Specifying the corresponding level of evidence rather than hiding the ultimate causal aim of a study is what we recommend (19), while acknowledging a margin of error in any empirical study (20).

Box 2. Examples of words and study elements that could point to causality or otherwise.

Words expressing a causal relationship	• Affect<br>• Attributable<br>• Benefit<br>• Cause/Causal pathway<br>• Contribute<br>• Determinant<br>• Effect<br>• Efficacy<br>• Impact<br>• Improve<br>• Leads to<br>• Mediates<br>• Responsible for<br>• Results in<br>• Safety
Words that could suggest causality in a given context	• Independently associated<br>• Induce<br>• Higher (lower) probability<br>• Modify<br>• Risk (factor)<br>• Trajectory<br>• (Quantitatively) decompose
Specific expressions avoiding suggestions of causal effects	• Association<br>• Correlation<br>• Less (more) likely link<br>• Predict<br>• Pattern
Key aspects suggesting causal aim	• Adjusting for confounders<br>• Discussing biological plausibility, dose-response and/or temporal relationship<br>• Discussing ‘unmeasured confounders’ assumption<br>• Mediation analysis<br>• Propensity score adjustment

For peer review only

	• (Propensity score) matching<br>• Providing estimates of (population) attributable risks<br>• Suggesting/recommending intervention<br>• Target trial emulation design<br>• Using directed acyclic graphs (DAG) to identify confounders and mediators<br>• Using negative controls<br>• Using instrumental variables

Conclusion

In summary, we have found that causal messages are embedded in studies otherwise carefully phrased in terms of association. Further guidance for authors is needed on what constitutes a causal statement, similar to the one published by Lederer et al (13) for Respiratory, Sleep and Critical Care Journals. We look forward to similar guidance for other disease groups. From the screened BMJ abstracts, we provided a list of expressions with clear interpretation which may inspire a useful more comprehensive compendium that can be derived from a consensus meeting, for instance. We argue that such awareness and special attention amongst authors and reviewers would serve our communication on the best available evidence for conceived interventions.

For peer review only

Contributorship statement

- Conceptualization: COP, LB, AD, SS, EG
- Methodology: COP, EG
- Data curation: COP, LB
- Formal analysis: COP, LB, EG
- Writing original draft: COP
- Writing-review and editing: LB, SS, AD, EG
- Approved final version: COP, LB, AD, SS, EG

Competing interests declaration

SS is a full-time employee of the BMJ Publishing Group. No other competing risks to declare.

Funding

This project has received funding from the European Union’s Horizon 2020 research and innovation programme under the Marie Sklodowska-Curie grant agreement No 676207.

Data sharing statement

The reviews and published versions of the papers included in the study are publicly available at BMJ.com. No further data will be made available as it is confidential submission data.

References

1. Begg MD, March D. Cause and association: Missing the forest for the trees. American Journal of Public Health. 2018;108(5):620-.

2. Hernán MA. A definition of causal effect for epidemiological research. Journal of Epidemiology and Community Health. 2004;58(4):265-71.

3. Kunz R, Oxman aD. The unpredictability paradox: review of empirical comparisons of randomised and non-randomised clinical trials. Bmj. 1998;317(October):1185-90.

4. Ruich P. The use of cause-and-effect language in the JAMA Network journals. 2017.

5. Cofield SS, Corona RV, Allison DB. Use of causal language in observational studies of obesity and nutrition. Obesity Facts. 2010;3(6):353-6.

6. Thapa DK, Visentin DC, Hunt GE, Watson R, Cleary M. Being honest with causal language in writing for publication. Journal of Advanced Nursing. 2020;76(6):1285-8.

7. Pinsky PF, Miller A, Kramer BS, Church T, Reding D, Prorok P, et al. Evidence of a healthy volunteer effect in the prostate, lung, colorectal, and ovarian cancer screening trial. American journal of epidemiology. 2007;165:874-81.

8. Food Drug Administration (FDA), Permutt T, Scott J. E9(R1) Statistical Principles for Clinical Trials: Addendum: Estimands and Sensitivity Analysis in Clinical Trials. 2017;9.

9. European Medicine Agency (EMA). ICH E9 (R1) addendum on estimands and sensitivity analysis in clinical trials to the guideline on statistical principles for clinical trials 2020 17 February 2020

For peer review only

10. Hernan MA. The C-Word : Scientific Euphemisms Do Not Improve Causal Inference From Observational Data. 2018;108(5):616-9.

11. Glymour MM, Hamad R. Causal thinking as a critical tool for eliminating social inequalities in health. American Journal of Public Health. 2018;108(5):623-.

12. Green MJ. Calculating Versus Estimating Causal Effects. American Journal of Public Health. 2018;108(8):e4-e5.

13. Hernán M, Robins J. Causal Inference: What If: Boca Raton: Chapman & Hall/CRC; 2020.

14. Goetghebeur E, Cessie Sl, De Stavola B, Moodie E, Waernbaum I. Formulating causal questions and principled statistical answers. 2019.

15. Adams RC, Sumner P, Vivian-Griffiths S, Barrington A, Williams A, Boivin J, et al. How readers understand causal and correlational expressions used in news headlines. Journal of Experimental Psychology: Applied. 2017;23(1):1-14.

16. Haber N, Smith ER, Moscoe E, Andrews K, Audy R, Bell W, et al. Causal language and strength of inference in academic and media articles shared in social media (CLAIMS): A systematic review. PLoS ONE. 2018;13(5):1-21.

17. Lederer DJ, Bell SC, Branson RD, Chalmers JD, Marshall R, Maslove DM, et al. Control of Confounding and Reporting of Results in Causal Inference Studies. Guidance for Authors from Editors of Respiratory, Sleep, and Critical Care Journals. Annals of the American Thoracic Society. 2019;16(1):22-8.

18. Harhay MO, Au DH, Dell SD, Gould MK, Redline S, Ryerson CJ, et al. Methodologic Guidance and Expectations for the Development and Reporting of Prediction Models and Causal Inference Studies. Annals of the American Thoracic Society. 2020;17(6):679-82.

19. Hernán MA, Robins JM. Estimating causal effects from epidemiological data. Journal of Epidemiology and Community Health. 2006;60(7):578-86.

20. Boutron I. Reporting and Interpretation of Randomized Controlled Trials With Statistically Nonsignificant Results for Primary Outcomes. JAMA. 2010;303(20):2058-.

21. Ghannad M, Olsen M, Boutron I, Bossuyt PM. A systematic review finds that spin or interpretation bias is abundant in evaluations of ovarian cancer biomarkers. Journal of Clinical Epidemiology. 2019;116:9-17.

22. Developing Guidelines for the Analysis of Randomized Controlled Trials in Real-World Settings [Available from: https://www.pcori.org/research-results/2015/developing-guidelines-analysis-randomized-controlled-trials-real-world.

23. Doan S, Yang EW, Tilak SS, Li PW, Zisook DS, Torii M. Extracting health-related causality from twitter messages using natural language processing. BMC Medical Informatics and Decision Making. 2019;19(Suppl 3).

24. Chiolero A. Causality in public health: One word is not enough. American Journal of Public Health. 2019;109(10):1319-20.

For peer review only

Tables

Table 1. Examples of statements found in the objectives and conclusions sections of abstracts of observational studies published in The BMJ in 2018 and their corresponding assigned category.

Assigned category	Abstract Objectives	Abstract Conclusions	Comment
Consistently causal	"...assess the effectiveness of..."	"Little evidence was found of a direct impact of..."	When discussing associations, words like effect, contribution or role are similar to cause and then (direct) impact and effect will be their consequence
	"To determine the effect of ... in ..."	"...has led to risk..."	
	"To describe the contributions of..."	"... an important role in ..."	
	"To evaluate the impact of ..."	"...impacts are..."	
	"To investigate whether improving adherence to ....“	"...the beneficial effect of improved..."	Evaluates taking an action 'improving adherence' and concludes that the effect is beneficial
	"...benefit of ... in reducing ... risk"	"... is an overlooked risk factor for ..."	Evaluates how a given intervention can reduce the risk of an outcome and then labels it as an 'overlooked risk factor'
	"To determine outcomes and safety of..."	"... is at least as effective and safe as ..."	Evaluates and determines that a certain intervention is as safe as the comparator

For peer review only

	"to quantitatively decompose this joint association to ... only, to ... only, and to their interaction."	"...excess risk of...These findings suggest that most cases of ... could be prevented by ..."	Suggests interest in direct and indirect effect i.e. mediation analysis, and concludes consequently
Consistently not causal (Associations)	"...is associated with ...compared with..."	"...is associated with ...compared with..."	Describes associations without labelling them as causal or prediction
	"To describe trends in..."	"...rates were high during the study period of ... with the highest rates in ... versus ..."	Limits to describe frequency
	"To assess how often ..."	"One in ... adults ... were ..."	
	"To examine the association between..."	"...could increase ... confirmation of these findings are warranted, preferably in an intervention setting."	Suggests further research to determine the nature of the association
	"...compared with...is associated with..."	"Additional studies, with long term follow-up, are needed to investigate the effects of..."	
Consistently not causal (Prediction)	"To develop and validate a set of practical prediction tools that reliably estimate the outcome of..."	"...prediction models reliably estimate the outcome..."	Describes developing and validating prediction models

For peer review only

	"To prospectively validate the ... algorithm to ..."	"...accurately classified..."	
Inconsistent	"...evaluate safety of..."	"...associated with..."	Phrasing the objective as causal and limiting to describing an association in the conclusion
	"...analyse the effect of..."		
	"...critical determinant..."		
	"...association with..."	"...is safe..."	Phrasing the objective as just to explore an association and presenting a causal claim in the conclusion
		"... had no substantial effect on long term survival..."	
		"... was determined by... may be largely explained by..."	
		"... was found to be the safest drug, with reduced risks of..."	
		"These results emphasise the benefit of..."	
	"...association with..."	"...tackling all these risk factors might substantially... "	Phrasing the objective and conclusion as if just to assess an association but then suggesting to take

For peer review only

		"…Targeting … prevention strategies among these patients should be considered."	action given the findings
		"Systematically addressing … may be an important public health strategy to reduce the incidence of"	
		"…present findings encourage the downward revision of such guidelines …"	

For peer review only

Table 2. Examples of (mis)matching causal and non-casual statements found in the objectives and conclusions sections of abstracts of observational studies published in The BMJ in 2018.

		Abstract Conclusions	
		Causal	Not causal
Abstract Objectives	Causal	Consistent<br>"...assess the effectiveness of..."and "Little evidence was found of a direct impact of..."<br>"...benefit of ... in reducing ... risk" and "... is an overlooked risk factor for ..."	Inconsistent<br>"...evaluate safety of..." and "...associated with..."<br>"...analyse the effect of..." and "...associated with..."<br>"...critical determinant..." and "...associated with..."
	Not causal	Inconsistent<br>"...association with..." and "...is safe..."<br>"...association with..." and "... had no substantial effect on long term survival..."<br>"...association with..." and "...tackling all these risk factors might substantially... "<br>"...association with..." and "Systematically addressing ... may be an important public health strategy to reduce the incidence of"	Consistent<br>"To describe trends in..." and "...rates were high during the study period of ... with the highest rates in ... versus ..."<br>"To assess how often ..." and "One in ... adults ... were ..."<br>"To develop and validate a set of practical prediction tools that reliably estimate the outcome of..." and "...prediction models reliably estimate the outcome..."

For peer review only

Supplementary Material

Table A. Statements in each of the included observational studies published in the BMJ in 2018

Published Abstract		Published Full text				Comment
Objective	Conclusion	Method	Confounder adjustment	Estimates provided	Authors Considerations	
CONSISTENTLY CAUSAL						
"To evaluate the **impact** of […] on […] in […]"	"[…] is associated with negative **effects** on […]. Given the relatively low prevalence of […], population level impacts are currently modest. Nevertheless, as […] has	"To estimate the **adjusted** odds ratio for each […] outcome by […] group, we created logistic regression models with […] as the reference group. […] The **population attributable risk** was calculated using the standard formula"	"Other subgroup analyses were done to ensure that […] association was **not confounded by** […]"	"[…] had 14% higher odds of […] compared with […] (adjusted odds ratio 1.14, 99% confidence interval 1.13 to 1.15). […] 14.5% (13.6% to 15.4%) of […] (under the assumption of a **causal** relation)	"The pooling of all […] during this period minimizes the risk of **confounding** from yearly fluctuations in […] outcomes. Finally, despite attempts to adjust […] using regression analysis and	Both abstract and main text use causal language: they state that their aim is to assess the impact of the exposure on the outcome, adjust for confounders, provide

	doubled in the US over the past generation, further investigation is warranted of the **impact** on [...] and public health.			can be **attributed to** [...]"	stratification, some **residual confounding** effects from [...] could remain. [...] As more than 12% of [...] **might have been prevented** were [...], the importance of these data are most relevant to [...]. The cumulative risk over [...] is also likely to be **important in terms of both economic burden and overall public health**"	population attributable risks and discuss residual confounding.
"To examine the [...] **risks of [...] initiation** compared with	"[...] **poses a [...] risk** compared with non-use, [...] use, and use	"[W]e conducted a series of cohort studies, each mimicking the strict design criteria of a clinical trial (a	"We calculated the **propensity score** for all eligible	"[...] initiators had a 50% **increased rate of** [...] events compared with	"We performed the following sensitivity analyses, [...] to	The use of causal language appears in

For peer review only

initiation of other traditional [...] drugs, initiation of [...], and no initiation."	of other traditional [...] drugs."	so-called **emulated trial design**), to compare rates of [...] among [...] with rates among [...]. [...] We estimated an observational analogue of the **intention to treat** hazard ratio, as a measure of the incidence rate ratio, by fitting a Cox proportional hazards model, using time since start of follow-up as the time scale and a time independent covariate for treatment assignment. We pooled data from all trials into one model and included each trial as a stratum in the regression (using values from 1 to 252)."	individuals initiating [...] at enrolment by fitting a logistic regression model including covariates on sex, age, year, comorbidity, and drug treatment use. We then **matched** non-initiators to [...] initiators (1:1) by **propensity score** within a maximum **matching** range of 0.025 and without replacement."	[...] non-initiators (incidence rate ratio 1.5, 95% confidence interval 1.4 to 1.7)."	estimate how strongly a single **unmeasured** binary **confounder** would need to be associated with [...] to fully explain our findings. [...] Finally, an **unmeasured confounder** that was twice as frequent among [...] initiators versus among non-initiators would still need to increase the risk of [...] by a factor of nine or more to fully explain the results, if no increased risk actually existed (eFigure 3). [...] Still, the	both abstract and main text. They apply causal methods including target trial emulation and propensity score matching and discuss residual confounding.

					**emulated trial design** lacked baseline randomisation, and therefore, **unmeasured confounding** cannot be excluded."	
"To explore **associations** between […] **and later**[…], **overall and by** […] **subtype** and timing of onset."	"[…] was associated with an **increased risk** of […], particularly [subtype]. […] were unlikely to **mediate** the associations substantially, suggesting that […] and […] may share underlying mechanisms or susceptibility pathways.	"We used Cox regression with age as the underlying time to estimate hazard ratios for […] comparing women with and without a history of […] We used competing risk methods when analysing associations with […] subtypes. […] We evaluated potential **mediation** by […]"	"We considered […] as a priori **confounders**."	"Women with a history of […] had a 53% **increase in risk of** […] overall, compared with women with no history of […] (incidence rate for women with a history of […]: 11.6 per 100 000 person years; incidence rate for women with no history of […]: 8.33 per 100 000 person years;	"we did sensitivity analyses […] using the array approach for testing the effect of an **unmeasured or incompletely measured confounder.** […] Sensitivity analyses suggested that **confounding** by […] was **unlikely to explain** the	Causal language is present in abstract and main text. They apply mediation analysis, adjust for confounders, discuss the unmeasured confounding assumption and residual confounding.

For peer review only

	Asking about a history of [...] could help physicians to **identify women who might benefit from** screening for early signs of disease, allowing for early clinical intervention.”			hazard ratio 1.53, 95% confidence interval 1.26 to 1.85).”	observed associations for [...]; in contrast, [...] **could** conceivably **explain** a considerable part of the association between [...] and [...]. [...] we also cannot rule out the possibility of **residual confounding** by other unmeasured covariates”	
“To investigate whether adults with [...] **are at an increased risk of** [...] and **whether the risk varies by** [...] severity and [...] condition activity over time.”	“Severe and predominantly active [...] are associated with an increased risk of [...] outcomes. **Targeting [...] prevention**	“We used Cox regression stratified by **matched** set [...] with current age as the underlying timescale to generate hazard ratios for the association between [..] and each [...] outcome (the unadjusted model). Subsequent multivariable	“For each patient with [...], we **randomly matched** up to five patients by age (within 15 years), sex, general practice, and calendar	“Table 3 shows that in the primary analysis, there was evidence of **associations** between [...] and all [...] outcomes, except for [...].	“Limitations of the study, inherent to most large observational studies, include the possibility for **confounding**, bias, and missing	The causal aim is evident in the abstract because they intend to assess how the risk of the outcome varies when

For peer review only

	**strategies** among these patients should be considered."	analyses adjusted for [...](the **adjusted** model). The adjusted model was further adjusted for variables which may have been on the **causal** pathway (ie, **mediators**) between [...] and [...] outcomes [...] (the **mediation** model). [...] The **population attributable risk** of each [...] outcome was estimated by using the estimated hazard ratio and assuming the prevalence of [...] to be 10%."	time at cohort entry. These unexposed patients were required to have at least one year of follow-up in CPRD and no history of [...] when matched. [...] We used a **directed acyclic graph** to inform the identification of covariates and **mediators** and to avoid **collider bias**"	**Associations were strongest with** [...] (hazard ratio 1.25, 99% confidence interval 1.11 to 1.41 in the adjusted model) and [...] (1.19, 1.10 to 1.30), with **partial attenuation in the mediation model**. [...] The greatest **population attributable risks** were estimated for [...] (2.4%, 1.1% to 3.9%) and [...] (1.9%, 1.0% to 2.9%)."	data. [...] We **have shown a clinically relevant increase in the risk of** [...] outcomes in patients with [...].This increased risk is largely confined to patients with severe or more active [...] and persists despite adjusting for potential mediators, including conventional risk factors for [...] outcomes. Consideration should be given to **developing prevention strategies to reduce the risk of** [...] among patients with	the exposure is modified and the conclusion is to take action given the findings. The main text uses causal language, discusses DAGs, mediators, collider bias and provides population attributable risks.

For peer review only

					severe or predominantly […]”	
“To **determine the effect of** […] outcome reporting in […] on risk averse clinical practice, “gaming” of clinical data, and 90 day postoperative mortality.”	“This study **did not find evidence that** the introduction of […] in […] **has led to risk** averse clinical practice behaviour or “gaming” of data. However, **its introduction coincided with** a significant reduction in 90 day mortality.”	“We used a **change point analysis** to study the change over time in **adjusted** 90 day mortality after […] and after […]. We used a multivariable logistic regression model for 90 day mortality, with a slope for calendar time and an interaction between time pre-introduction versus post-introduction of […], in addition to all of the **risk adjustment variables**. This modelled a change in the slope of mortality at the point that […] was introduced but no immediate change in mortality.”	“The **risk factors** included in this logistic regression model are […]. An adjusted outcome was then produced by indirect **standardisation**”	“The 90 day mortality in patients undergoing an […] fell during the study period from 952/33 638 (2.8%) before the introduction of […] to 552/25 905 (2.1%) after (fig 4). Therefore, we carried out change point analysis which showed a steeper decline in 90 day mortality after the introduction of […] (P=0.03). The **change point analysis also found a**	“If we assume that the decrease in mortality can be **causally linked** to […], the process of […] This team response could have been **mediated** through […]”	Causal language is present in abstract and main text as they state that their aim is to determine the effect of the exposure on the outcome and conclude that the exposure has not led to the outcome. The main text explainis that confounder adjustment was made through standardisatio

				**significant effect of** […] when it was modelled as an immediate shift in 90 day mortality (P=0.01) and when it was modelled as both an immediate shift and a change in slope (P=0.04)."		n and discuss possible mediators for this relationship.
"To assess the **effectiveness of** the […] policy initiatives **in improving** […] **and reducing** […] in survival in England."	"Little evidence was found of a **direct impact** of […] on one year survival, and no evidence for a reduction in […] in cancer survival. These findings emphasise that […] in survival remain a major	"We estimated one year **net survival** for each […] by sex, year of diagnosis (1996 to 2013), and deprivation category. Patients with a diagnosis between 1996 and 2013 had the potential to be followed up for at least one year, so we used the classic cohort approach. […] We estimated net survival using the	"Survival estimates for all ages combined were age **standardised** with the International Cancer Survival Standard weights. […] We used multivariable linear regression	"One year survival **improved** for 20 of the 21 […] examined in women and 16 of the 20 […] examined in men. […] For these […], the average annual absolute increase in one year age	"The lack of **consistent** results between men and women, as well as the lack of general patterns across […] types, provide **little evidence for any strong impact** of the […] policies on short term […] survival. The	Causal language is present in both abstract and main text. The aim is to evaluate the effectiveness of a policy on a given outcome, they provide standardised

	public health problem for a healthcare system founded on equity.”	consistent nonparametric estimator defined by Pohar-Perme.”	to investigate the survival patterns for each […] and by sex”	**standardised net survival** was often greater than 1% over the whole study period”	evidence is even weaker for their **impact** on the […] in […] survival. […] **These findings should be taken into consideration by** […] policy makers and inform future initiatives.”	net survival and suggest to take action given the findings.
To investigate whether **improving** adherence to […] **interacts with** the genetic predisposition to […] in relation to long term changes in […] and […].”	“These data indicate that improving adherence to […] could **attenuate** the genetic association with […]. Moreover, the **beneficial effect** of improved […] on […] was particularly	“We used multivariable generalized linear models with repeated measures analyses to assess the main **associations of** the […] **and changes in** the […] **with change in** […]”	“We used multivariable models to **adjust for** […]”	“In general, the […] was **associated with increases in** […] every four years: in the two cohorts combined, each additional […] was associated with 0.02 (SE 0.01) increase in […] and 0.05 (SE 0.03) kg increase in […]”	“[…] **unmeasured or unknown confounders** may also exist. Secondly, because adherence to […] was not randomized, the association between […] and […] may not imply a **causal relation.** Thirdly, the results could be	Assessing if improving adherence has an effect translates to an intervention that is being assessed. They conclude that there is a beneficial effect and suggest to take action. The

	pronounced in people at high genetic risk for […].				underestimated by potential **reverse causality**. […] Our study provides reproducible evidence from two prospective cohorts of US men and women that **improving […] could attenuate** the […] association with […]”	main text discusses unmeasured confounding assumption and reverse causality. All of the above is consistent with a causal aim.
“To assess the **independent** and joint **associations** of [….] and […] with […] risk and to explore the **benefit of […] in reducing** the […]	“[…] is an overlooked **risk factor** for […], as important as five major lifestyle factors combined. In this study, […] **contributed** to more than one	“We calculated the time to event from the date of enrollment to the date of […] incident or […] death, death due to causes other than […], or the end of cohort follow-up (31 December 2008), whichever came first. We used Cox proportional	“[…] **adjusting for** […].”	“A statistically significantly increased risk of incident […] was observed for the eight diseases and markers. Specifically, […] was inversely associated with	“More evidence was needed to clarify whether the inverse association was **causal** or related to […]. […] the **dose-response relation**, the exclusion of […]	Causal language is present in abstract and main text. They identify that the exposure contributes to the outcome,

For peer review only

risk associated with [...] and [...].	fifth of the risk for incident [...] and more than one third of the risk for [...] death. [...] is associated with a nearly 40% **reduction in the [...] risk** associated with [...]."	hazards model to estimate hazard ratios and 95% confidence intervals"		risk of incident [...] in a **dose-response manner**"	during recent follow-up, and further adjustment for [...] minimize the likelihood of **reverse causation** and lend support for **causality**. [...] Our study uncovered a **substantial impact** of [...] jointly on [...] risk, which were equally as important as five lifestyle factors combined."	after adjusting for covariates. They discuss dose-response relationship and reverse causality .
"To examine the **association** between [...] and [...] **in later life**, and **determine** whether the maintenance of	"These results show that [...] is **not associated with the trajectory of** [...] in late life, but is	"The raw scores from the [...] tests were **standardised to** a mean of 100 and a standard deviation of 15 to produce an [...] scale. Age at testing was the number of years	"Because our sample were all born in the same year and tested at a similar age, a **confounder** for age at entry was	" The typical intellectual engagement models for each domain are shown in table 2 and indicated **an**	"In our statistical models, we introduced **possible confounders** available from early life and life	The causal aim is suggested when the goal is to establish temporal relationship between

For peer review only

[…] will offset age related […]”	associated with the acquisition of ability during the life course. Overall, findings suggest that high performing adults engage and **those that engage more being protected from** relative decline.”	after participants’ 60th birthdays. We modelled age in this form so that the intercept occurred at age 60 years rather than zero years, such that the calculation for the intercept would represent a realistic adult value rather than one extrapolated 64 years earlier. […] For each model, a probability value of P<0.05 was considered significant.”	not used. We modelled cognitive performance with a linear mixed model, as a combination of […].”	**expected significant decline** in […] with age, ranging from −1.09 to −1.31 standard points per year for the […] test and −0.77 to −1.69 for the […] test. […] None of the age×TIE interaction terms were significant, indicating that […] **did not influence the trajectories of** age decline.”	course, including […]. We also **controlled for** […] associated with repeated testing. […] significant **associations remained after adjustment for** age, sex, and test practice effects. […] is an **independent contributor to** late life […] and has a unique effect over and above the effect of other life course variables. […] It is, however, impossible for a **causal effect** to be inferred […]”	exposure and outcome, describing the exposure as a trajectory, providing standardised and adjusted estimates and considering whether it is possible to infer a causal link.
“To evaluate the **associations** of a	“ In this cohort study, […] were	“To test the **association** of […] and […] with […] we	“Cox proportional hazards models	“In Cox proportional	“The present study provides	The abstract describes that

[…] and […] with **incident** […].”	**independently associated with incident** […]. These results **emphasise the benefit of** entire populations adhering to […], independent of […] risk.”	used Cox proportional hazards models. The duration of follow-up was calculated as time between the baseline assessment and the first event of either […] or 1 March 2016, which was the end of follow-up for the current data release. Participants who had a […] before a […] occurred were censored at the time of the respective event.”	included **adjustment for** age and sex for the lifestyle score models. For the models including the genetic score we additionally **adjusted for** the first 10 principal components of ancestry and genotyping batch.”	hazards analysis, the **risk of** […] was higher for those with […] (hazard ratio 1.20, 95% confidence interval 1.08 to 1.34) and […] (1.35, 1.21 to 1.50) **compared with** those with a low genetic risk score”	further support that common […] are **implicated in the development of** […]. […] The […] was also associated with […], which suggest that the **effect of** the […] on risk of incident […] might at least in part be **mediated by** […]. The effects of […] might differ according to the cause of […], although some […] factors are shared between two or more **causal** factors”	the goal is to establish a temporal relationship between the exposure and the outcome and suggest to take action given the findings. The main text describes adjusting for covariates and discusses mediation.
“To determine the **longitudinal association**	“In older adults, a **higher cumulative**	“We used a Cox proportional hazards model to evaluate the	“We selected covariates and potential	“Figure 1 shows that **after multivariable**	“[…] we excluded participants […] who reported	The abstract describes that the goal is to

For peer review only

between […] and […].”	**level of** […] was associated with a **higher likelihood** of […]. These findings **support guidelines for** increased dietary consumption of […] in older adults.”	**association between time-varying** […], **adjusting for time-varying covariates** (updated at […] measurement), and the likelihood […]. Time at risk was from the first […] measurement until the first […] event or censoring […] or the latest date of adjudicated follow-up in June 2015.”	**mediators** based on biological interest, current or previously observed associations with […], and meaningful changes in the exposure risk estimate (±5%). Minimal **adjustments included** age and sex. **Multivariable adjustments** additionally included […]. We used the potential mediators to explore what additional associations could exist to these potential **pathways**.”	**adjustment for** demographic, lifestyle, cardiovascular risks, dietary habits, and other […], higher […] levels were associated with a lower likelihood of unhealthy ageing. Overall, participants in the highest group of […] had an 18% (95% confidence interval 3% to 30%; P=0.001) lower risk of […].Findings were not appreciably altered after adjustment for potential **mediators** (not shown).”	baseline […] (to avoid **reverse causality**; n=195). […] The community based design improves **generalizability**, and regular physical examinations ensured that demographics and other risk factors were well measured, which may help to minimize **confounding**. […]The possibility of **residual confounding** by imprecisely measured or unknown factors also cannot be excluded for an observational study. […] *Any unmeasured*	establish a temporal relationship between the exposure and the outcome and suggest to take action given the findings. The main text describes adjusting for covariates and discusses mediation, residual confounding and reverse causality. The statement in italic is a clear causal statement.

For peer review only

					confounders would have to be strongly associated with both the exposure and the outcome, conditional on all the variables already in the model. Thus, it seems unlikely that either poorly measured or unmeasured confounders could fully account for our findings."	
"To **prospectively** evaluate the joint **association** of […] and […] with risk of type 2 diabetes risk, and to **quantitatively decompose** this	"Among female nurses, both […] were associated with a higher risk of […]. **The excess risk of** […] was higher than the	"Participants contributed person time from the return of the baseline questionnaire [..] until the date of diagnosis of […], death, loss to follow-up, or the end of the follow-up period (30 June 2012 for	"Information on **potential confounders** was assessed and updated every other year via the questionnaires throughout	"We observed a positive association between duration of […] and risk of […] in both cohorts. Compared with	"From a public health standpoint, because 71% of the joint effect could be attributed to an […], our findings	Their aim is to establish a temporal relationship exposure-outcome, they discuss adjusting for

For peer review only

joint association to […]”	addition of risk associated with each individual factor. These findings suggest that most cases of […] **could be prevented by** […], and the **benefits** could be greater in […].”	the NHS and 30 June 2013 for NHS II), whichever came first. We used **multivariable time dependent** Cox proportional hazards models to estimate hazard ratios and 95% confidence intervals for the associations between […] alone and in combination with […].”	follow-up. This information included […] In multivariable analysis, we **adjusted for** several **confounding** factors including […] We also examined the **decomposition of the joint effect**: the proportion **attributable** to […]”	women without rotating night shift work, the pooled **multivariable adjusted** hazard ratios for women with 1-5, 5-9, and 10 or more years of […] were 1.11 (95% confidence interval 1.00 to 1.22), 1.28 (1.10 to 1.49), and 1.46 (1.33 to 1.62) (P for trend <0.001) […](table 3). […] The **attributable** proportions of the joint **effect** were 17.1% (95% confidence interval 14.0% to 20.8%) for […] alone, 71.2% (66.9% to 75.8%) for […]alone, and 11.3% (7.3% to	underscore the importance of maintaining […].Our findings suggest that most cases of […] could be **prevented by** […], and the benefits would be larger in […] “	confounders, provide excess risk and suggest to take action given the findings to prevent the outcome.

				17.3%) for their interaction.”		
“To assess the **association** between […] and seven serious **adverse events** of current concern.”	“In this analysis of nationwide registers from two countries, use of […], as compared with […], was associated with an **increased risk of** […], but not with other serious **adverse events** of current concern.”	“Patients were followed from cohort entry to treatment cessation, crossover to the other study drug […], the outcome event, death, emigration, or the end of the study period (31 December 2016). We used Cox proportional hazards regression to calculate hazard ratios, analysing each outcome independently. The **absolute risk difference** was calculated as hazard ratio−1 multiplied by the rate in the comparator group.”	“We used an active comparator new-user study design and controlled for a wide range of potential **confounders** (patient characteristics that might be associated with both the outcome and the decision to initiate a drug) through a non-parsimonious **propensity score model** to minimise the risk of bias, including **confounding by indication** […].	“Use of […], as compared with […], was associated with an increased risk of […] (hazard ratio 2.32, 95% confidence interval 1.37 to 3.91) and […] (2.14, 1.01 to 4.52) but not with […] (1.11, 0.93 to 1.33), […] (0.69, 0.45 to 1.05), […] (0.89, 0.67 to 1.19), […] (0.99, 0.71to 1.38), or […] (1.16, 0.64 to 2.12).”	“The findings should be interpreted in the context of limitations of observational studies and the uncertainty of the **effect estimates**. […] Therefore, the studies could suffer from compromised **confounding** control, as indicated by the imbalance in […] at baseline between users of […] versus comparators, even after propensity score matching […]	The goal is to evaluate the association with the exposure to adverse events which translates into assessing safety. They discuss adjusting for confounding, resort to propensity score matching and consider residual confounding.

For peer review only

			We estimated **propensity scores** by using logistic regression for the probability of [...] conditional on the status of 66 covariates, defined and selected a priori, including sociodemographic characteristics, comorbidities, comedications, and healthcare utilisation [...] We matched [..] and [..]t users (1:1 ratio, by country) according to propensity score, by using the nearest neighbour matching algorithm (caliper width 0.2 of the		Finally, **residual and unmeasured confounding** affecting the findings in our study cannot be ruled out."	

For peer review only

			standard deviation of the logit score). Analyses were performed in a pooled dataset of the two countries.”			
“To determine whether patients […] have **fewer** […] and **higher rates** of […] than patients […].	***In results*: “Per 100 000 patients, there were 2999 **fewer** follow-up appointments within 14 days, 26 **excess** deaths, […] **attributable to** […].”<br><br>“Patients […] are **less likely to** have […] and are at higher risk of […].”	“For all outcome comparisons we report unadjusted and **adjusted** odds ratios (with 95% confidence intervals). **Adjusted** odds ratios were obtained with logistic regression models estimated using generalised estimating equations methods and including all measured patient and hospital characteristics.”	“We examined hospital type and several characteristics of patients and admissions: year of Charlson comorbidity index score, socioeconomic status (measured using median neighbourhood income), length of hospital stay, arrival by ambulance, diagnosis,	“Patients […] were **less likely** to have follow-up with a physician within seven days (36.3% v 47.8%, adjusted odds ratio 0.61, 95% confidence interval 0.60 to 0.62) and 14 days (59.5% v 68.7%, 0.65, 0.64 to 0.66). […] Patients discharged during the holiday period were at	“A confirmatory time-to-event analysis in a **propensity score matched cohort** (see supplementary appendix table 5) showed consistent results (death or readmission hazard ratio 1.08, 95% confidence interval 1.07 to 1.09). […] The differences in outcomes **could**	The abstract and main text use causal language: they discuss risk attributed to the exposure, provide adjusted estimates using propensity score matching and discuss residual confounding.

			discharged with home support or against medical advice, and previous healthcare usage (emergency department visits, hospital stays, outpatient visits, home care visits). hospital discharge, age, sex, rural residence, ”	**increased risk of** death or readmission within 30 days (25.9% v 24.7%, 1.09, 1.07 to 1.10). This was **explained by** an increased risk of return to the emergency department (24.3% v 23.0%, 1.09, 1.07 to 1.10), rehospitalisation (11.8% v 11.4%, 1.06, 1.04 to 1.08), and death (1.5% v 1.5%, 1.06, 1.02 to 1.10) within 30 days”	**not be explained by** observed hospital or patient characteristics, including admission diagnosis. […] the possibility of **confounding** due to unmeasured differences remains”	
“To investigate the **association** between […] and	“Use of […] is **associated** with a reduction in	“We calculated age **standardised** incidence rates of […] per 100 000	“**Adjusted** incidence rate ratios (referred to	“The age **adjusted** incidence of […]	“The data linkage study design also enabled us to	When assessing the exposure-

For peer review only

overall and specific types of […].”	[…] risk in women of reproductive age—an **effect related to duration of use**, which diminishes after stopping use. These data suggest no **protective effect** from […].”	person years, using the age distribution of the cohort as standard. Risk of […] among users of the different product groups was analysed by a Poisson regression model in SAS version 9.3 (SAS Institute). […] We calculated the population prevented fraction (population prevented fraction = $\text{prevalence}_{\text{exposure}}(1-\text{relative risk})$) associated with ever use of […] by using the relative risk of never use versus ever use of […]. The population prevented fraction is the proportion (expressed as a percentage) of the […] in the cohort that has been prevented by ever use of […].”	here as relative risks) and their surrounding 95% confidence intervals were calculated for each model, with never users as the reference group. The **adjusted** models included the following time varying covariates […]”	was highest in women who were never users of […] (7.5 per 100 000 person years; table 2). Among ever users of […], **reduction** in the age standardised absolute rate of […] was 3.2 per 100 000 person years. Overall, ever users of […] had a reduced risk of […] compared with never users (relative risk 0.66 (95% confidence interval 0.58 to 0.76)). […] **use of […] prevented** 21% of […] in the study population”	**adjust** for several important **confounding** variables. We were not able to adjust for some factors, such as […] Our findings, therefore, could be subject to **residual confounding**.”	outcome relationship, they provide standardised estimates, discuss residual confounding, dose-response and provide attributable fraction.

"To assess whether […] is **associated with a reduction** in […] and mortality in old and very old adults with and without […]."	"In participants older than 74 years without […], […] was **not associated with a reduction in** […] or in all cause mortality, even when the incidence of […] was **statistically significantly higher** than the risk thresholds proposed for […]. In the presence of […], […] was statistically significantly associated with reductions in the incidence of […] and in all cause mortality. This **effect** decreased after age 85 years	"To prevent survivor bias and covariate measurement bias, we selected a "new users design" over "all […] users. […] Using Cox proportional hazard regression models **adjusted by propensity score**, we calculated the hazard ratios of statin use for the outcome events. Participants were censored at the date of transfer from SIDIAPQ or at the end of the study period. "	"We explored the variables associated with […] to determine candidate variables for the propensity score of […]. From SIDIAPQ we obtained data on age, sex, […]Because of non-random treatment allocation, we used a logistic model based on **potential confounding** covariates to calculate the **propensity score** of […]. We calculated the propensity score separately for participants with and without […]	"In participants without […], the hazard ratios for […] were 0.94 (95% confidence interval 0.86 to 1.04) for […] and 0.98 (0.91 to 1.05) for all cause mortality in 75-84 year olds. […] The one year **number needed to treat** was 164 for […] and 306 for all cause mortality."	"To prevent **residual confounding** we performed additional regression adjustments after adjustment of **propensity score**. Variables that remained imbalanced after propensity score adjustment were also included in the models. […] Despite these efforts, we acknowledge that some **residual confounding** might exist."	They use causal language and causal methods. To estimate the effect of the exposure on the outcome. they use propensity score methods to adjust for confounding, provided a NNT and consider residual confounding.

	and disappeared in nonagenarians. ”		and also within each age group, and standardised differences before and after adjustment for **propensity score**. Variables with standardised differences <0.10 were considered to be **well balanced**.”			
“To examine the **association** between […] and risk of […].”	“The risk of […] was increased in people who […]. In several countries, guidelines define thresholds for […]. The present findings **encourage the downward**	“Cox regression was used in all analyses, with age as the timescale to model the associations with hazard of incident […]. Participants were censored at date of record of […], death, or 31 March 2017, whichever came first.”	“Models were first **adjusted** for sociodemographic factors, then additionally for health behaviours, and finally for health status.”	“[…] was associated with a **higher risk** of […] when the reference was […]; in these analyses […] was associated with an increased risk of […] in a linear fashion (among those […], P for non-	“[…] multistate models showed that part of the excess risk of […] in […] was **attributable to** the greater risk of […] in this group. […] We accounted for several sociodemographic and health	The abstract and main text use causal language, they provide adjusted, discuss residual confounding and suggest to take action given the findings.

For peer review only

	**revision of such guidelines** to promote [...] at older ages."			linearity=0.97 using spline regressions). In a model **adjusted for** sociodemographic factors [...] was associated with a **greater risk** of [...] (hazard ratio 1.47, 1.15 to 1.89) compared with [...]"	related characteristics in the analysis, but **residual confounding** cannot be excluded as an explanation for the higher risk of [...] among [...]. Our multistate models lent partial support for a **mediating** role of [...] in the association between [...] and increased [...]"	
"To assess whether **adding or switching to** [...] is associated with an increased risk of [...], **compared with remaining** on [...]	"[...] as second line drugs are associated with an increased risk of [...] compared with remaining on [...]. Continuing	"The study cohort was formed by identifying all subjects from the base cohort of [...] initiators who subsequently added or switched to a [...] as second line treatment. Patients who added or switched to	"[...] exposed and reference subjects were **matched on high-dimensional propensity score**. The high-dimensional	"Compared with the use of [...], adding or switching to [...] was associated with an increased risk of [...] (7.8 v 6.2 per 1000	"Based on a post-hoc analysis, the findings of the primary analysis on [...] unlikely to be the result of an **unmeasured confounder**	The aim is to assess the effect of the intervention strategy of adding or switching to a particular

For peer review only

in patients with […].”	[…] when introducing […] appears to be **safer** than switching.”	other […] were censored. For each patient adding or switching to a […], we identified a matched reference patient who also was a […] initiator but remained on metformin, using a prevalent new-user design. […] we constructed a Cox proportional hazards regression model for each outcome that estimated the hazard ratio and the 95% confidence intervals for […] versus […].”	**propensity score method** empirically selects covariates based on their prevalence and potential for **confounding**. For each member of each matched set, we identified all available information from seven data dimensions (five dimensions from the CPRD: drug prescriptions, procedures, diagnoses, disease history, and administrative information; two dimensions form the HES: diagnoses and procedures) in the one year	person years, hazard ratio 1.26, 95% confidence interval 1.01 to 1.56), all cause mortality (27.3 v 21.5, 1.28, 1.15 to 1.44), and […] (5.5 v 0.7, 7.60, 4.64 to 12.44). There was also a trend towards increased risks of […] (6.7 v 5.5, 1.24, 0.99 to 1.56) and […] (9.4 v 8.1, 1.18, 0.98 to 1.43).”	under most plausible exposure-confounder and confounder-outcome associations. […] For our study, we used the recently developed prevalent new-user design. To **emulate** the randomised controlled trial, this design identifies (at the doctor visit that led to the patient on […] adding or being switched to […]) a comparable patient with the same history of […] use and of other characteristics, but who on that	drug, they emulate a target trial, use propensity score matching. discuss residual confounding and conclude that the strategy is safe.

			period before the date of the **matched** set. We then applied conditional logistic regression to estimate the **propensity** of receiving a […] drug, thereby considering the 500 most likely **confounders**.”		visit continued on […]. […]owing to its observational nature there is the potential for **residual confounding**.”	
“To investigate the **associations** between […] and **risks of** […] compared […].”	“Overall, […] was found to be the **safest** drug, with reduced risks […] compared with […]. […] and low dose […] were, however, associated with increased risks of all cause mortality	“We used a new-user design to capture all events occurring after starting treatment and to reduce the impact of **confounding**. […] Incidence rates for each outcome were calculated based on the numbers with the outcome and the person years of follow-up, and were age and sex standardised for each	“**Confounding** factors. It is possible that patients at higher risk of […] may preferentially be prescribed […] rather than […], so all analyses were **adjusted for** demographic and clinical variables, either because	“In patients with […], […] was associated with a lower risk of […] than […] (adjusted hazard ratio 0.66, 95% confidence interval 0.54 to 0.79). […]Table 5 shows the **number needed to treat** or	“Although many adjustments have been done using the data available on the existing databases, there is a possibility of **unmeasured confounding** or **confounding** by indication. […] Although we used a proportional	The aim is to assess the safety of a drug and they use different strategies to adjust for confounding, including propensity scores, provide NNT and discuss the

For peer review only

	compared with […].”	drug. To estimate the risks associated with each […], an outcome specific Cox model containing all **confounding** factors was used, with […] as a primary reference.”	they may have been used as indicators for prescribing a specific […] or because they have possible associations with increased risk of […]. We similarly adjusted for comorbidities, previous events, and drugs also used as indicators or associated with increased risks.”	**number needed to harm** to measure the relative benefits or risks of […] in comparison with […].”	hazard model adjusting for all available **confounding** factors, we also undertook a sensitivity analysis using the **propensity score method** and obtained very similar results.”	unmeasured confounding assumption.
“To examine the **association** between […] and the **risk of** developing […].”	“Our study indicates that adherence to a […] is **associated with a substantially reduced risk of** […]. These	“To evaluate the **association** between […] and […], we calculated relative risks and 95% confidence intervals using multivariable log-binomial regression models with generalized estimating	“We first evaluated associations with […] by categories of each low risk factor, **adjusting** for […].”	“In multivariable analyses (model 2), […] had a **relative risk** of 3.10 (95% confidence interval 2.69 to 3.57) of […],	“Several factors could contribute to the weak **mediation effect** of […] in the association between […] and […] risk. […]	They provide adjusted estimates, discuss mediation and residual confounding and suggest to

For peer review only

	findings highlight the **potential benefits of implementing** […] interventions to curb the risk of […].”	equations and specified an exchangeable correlation structure.”		compared with […].”	Another limitation, as in any observational study, is that we cannot exclude the possibility of **uncontrolled confounding** by […] or **residual confounding**. […] Our findings highlight the potentially critical role of […] in the **etiology** of […] and lend support to […] based **intervention strategies** for reducing […].”	take action given the findings.
“To determine **rates of** […] and all cause mortality in patients with […] compared to	“Patients with […] remain at higher risk of […] than patients without […].	“We carried out two retrospective cohort studies to determine incidence rates of […] (primary outcome) and all cause mortality (secondary	“Poisson regression was used to calculate **adjusted** incidence rate ratios, adjusting	“The crude incidence rate ratio was 0.73 (95% confidence interval 0.65 to 0.81, P<0.001).	“In light of the evidence produced by this study, it is recommended that clinical	Even though the use of causal language is not explicit, they compare rates

For peer review only

patients with […] and without […].”	The **risk** is increased even in those in whom […] is not documented. **Guidelines** should be updated to advocate continued use of […] in patients with […]”	outcome)in patients with […] versus randomly selected **matched controls** with […]. We calculated crude and **adjusted** incidence rate ratios comparing the incidence of […]”	for the baseline covariates […]”	Adjusting for potential confounders […] made little difference to the incidence rate ratio: 0.76 (95% confidence interval 0.67 to 0.85, P<0.001)”	guidelines and schemes designed to incentivise appropriate management […] are updated”	of the condition in the different groups that have been matched, provide adjusted estimates and suggest to update guidelines to reflect the findings.
“To examine the **association** between […] at […] and […].”	“[…] during the period […] iis **safe** with respect to the risk of […].”	“We estimated odds ratios of […] and […] and associated Wald type two sided 95% confidence intervals by logistic regression. For […] and […], we calculated hazard ratios and associated Wald type two sided 95% confidence intervals from Cox regression models, which allow for detailed	“[…] to adjust for potential **confounding** due to temporal trends, we included […]. […] are well established risk factors for […]. […] All estimates were calculated by models with	“In analyses without covariate adjustment (model 1), […] was **associated** with an increased risk of […] (hazard ratio 1.69 (95% confidence interval 1.18 to 2.41)) and […] (2.14 (1.39 to	“[…] is **not causally related** to increased risks (…). Instead, our results suggest other factors underlying and confounding the associations between […]. Furthermore, although our	The abstract suggests a causal aim when describing the intention to establish a temporal relationship between the exposure and the outcome

		adjustment for censoring affecting the length of follow-up of each child. Days since birth was used as the underlying time scale. Each child was followed from birth until a diagnosis of the outcome, death, or end of follow-up at 31 December 2014, whichever event occurred first. ”	increasing complexity, beginning with models without adjustment for covariates (model 1), followed by models adjusting for all included potentially **confounding** covariates (model 2).”	3.30); fig 2). After covariate **adjustment** (model 2), [...] was only associated with an increased risk of [...] (adjusted hazard ratio 1.66 (1.06 to 2.59); fig 2).”	results suggest that [...] are not causally associated [...] could be a causal factor for other outcomes. [...] although the present study did not find a causal link [...], replication of the results is imperative.”	and concluding that the exposure is safe. The main text uses causal language explicitly when describing the strategies to control for confounding and concluding that a causal relationship was discarded.
“To assess the association of [...] and risk factors for [...] with [...] at [...].”	“The **independent association** between [...] and [...] in [...] is comparable in strength and consistency with those for [...]. The results	“We used a generalised additive mixed model (GAMM) to estimate [...], with [...] as fixed effect predictors and [...] as random effect at the intercept and [...] slope. [...] We computed 95% confidence intervals from the uncertainty of the	“We considered [...] as potential confounders.”		“As our analyses relied on cross sectional data, these findings should be interpreted cautiously and should not be considered as causal estimates	The abstract suggests a causal aim when describing the intention to establish a temporal relationship between the

	of this study suggest that **tackling** all these risk factors might substantially increase life years spent in good physical functioning.”	estimated smoothing function. We computed the **number of years of functioning lost** from the mixed model predictions”			of the impact of [...] on [...]. [...] Given that the present study is based on observational data, our study informs about associations but cannot provide evidence of causality.”	exposure and the outcome and concluding that the link is comparable to those for other established risk factors. It is important to note that In the abstract the design of the study is described as “Multi-cohort population based study”. However the method and discussion refer to a “cross sectional” design that limits the possibility to establish causal links.

For peer review only

"To determine outcomes and **safety** of [...] for [...], due to [...], in routine clinical practice."	"In routine clinical practice, [...] for patients with [...] is at least as **effective** and **safe** as in the setting of a randomised controlled trial."	"We used regression models to compare baseline characteristics and outcomes in patients [..] with those in the [...] intervention and control arms. The effect of [...] on [...] at 90 days in patients [...] compared with [...]l was expressed as an **adjusted** common odds ratio, derived from multivariable ordinal logistic regression (shift analysis)."	"We **adjusted** for [...]"	"After **adjustment** for [...], the shift towards [...] was significant for patients [...] compared with those [...] intervention arm (adjusted common odds ratio 1.30, 95% confidence interval 1.02 to 1.67; P=0.03) and control arm (1.85, 1.64 to 2.34; P<0.01; fig 1). "	"The results of our study might have important implications for the future of [...] for [...]. [...] is at least as **effective** and **safe** as in the setting of a randomised controlled trial."	The abstract and main text point to a causal aim as the intention is to assess the safety of an exposure in relation to an outcome and the conclusion is that not only is safe but also effective.
"To investigate whether [...] is **associated** with an **increased risk** of [...]."	"In a **propensity score matched** cohort, [...] use was associated with an increased risk of [...]. This	"Cox proportional hazards regression, with days since start of treatment as the time scale, was used to estimate the hazard ratio for [...], comparing	"We used two major strategies to control for **confounding**. To account for potential **confounding** by	"There was an increased risk of [...] associated with [...] (hazard ratio 1.66; 95% confidence interval 1.12 to	"An important concern in any observational study is the possibility of confounding. We used an active	Both the abstract and main text use causal language and causal methods

	association appeared to be largely **driven by** [...].”.	episodes of [...] and [...] use.”	indication [...], we used an **active comparator design**, [...] To control for potential **confounding** from differences in baseline health status, we used a **propensity score matched** design, taking into account demographic characteristics, medical history, [..].”	2.46). This increase corresponded to an absolute difference of 82 (95% confidence interval 15 to 181) cases of [...] per 1 million treatment episodes in the 60 day risk period.”	comparator to limit confounding by factors associated with [...], including confounding by indication, and propensity score matching derived from a range of covariates. Despite this, the possibility of residual confounding (for example, due to [...]) cannot completely be ruled out.”	including propensity score matching. They discuss the possibility of residual confounding mainly because of the observational nature of the study but also suggest possible confounders missed.
“To examine the **risks** of [...] in patients with [...] and in a general population comparison cohort.”	“[...] was associated with increased risks of [...]. [...] may be an important risk factor for [...].”	“We did a population based **matched cohort** study based on routinely and prospectively collected data. [...] We calculated the 0-1 year, >1-5 years, and >5-19 years	“Using the full hospital history (inpatient and outpatient diagnoses) recorded in the DNPR before the	“After adjustment for the covariables, [...] was associated with [...] (adjusted hazard ratio 1.49,	“Although we and adjusted the analyses for a wide range of potential **confounders** identified a priori	Both the abstract and main text describe the exposure-outcome relation in a

For peer review only

		cumulative incidence per 1000 people for each outcome, accounting for the competing risk of death. Correspondingly, we used matching factors stratified (conditional) Cox proportional hazards regression to estimate hazard ratios, adjusting for the categorical comorbidities listed above as covariables."	index date, we obtained information on the following [...] risk factors: [...]"	95% confidence interval 1.36 to 1.64), [...] (2.26, 2.11 to 2.41), and [...] (1.94, 1.68 to 2.23), as well as [...] (1.59, 1.45 to 1.74) and [...] (1.25, 1.16 to 1.36) (fig 2). We found no association with [...] (adjusted hazard ratio 1.12, 0.96 to 1.30) or [...] (1.04, 0.93 to 1.16).	on the basis of excisting literature, we cannot exclude influence of unknown or **residual confounding**, for example, by [...]"<br><br>** Typos copied as in the published version	matched cohort. They discuss the possibility of residual confounding and suggest possible confounders missed. All of these elements point to a causal aim.
"To determine if [...] is a **critical determinant** of [...] and [...]."	"[...] does not have a **clinically important association** with [...] or [...]."	"We assessed the effect of [...] compared with [...], using multivariable regression. Modified Park's tests were used to determine the appropriate regression models (gamma, Poisson, and logistic) for discrete [...]	"In all of our primary analyses we adjusted for the following key **confounders**: [...]"	"Table 2 shows that there was no strong evidence of a **clinically important association** of [...] and [...] with [...] or [...]."	"We recognise that we assessed multiple associations and the isolated positive association of [...] and [...] may reflect a chance	Both abstract and main text use causal language and explain that the aim is to identify whether the exposure is a

For peer review only

		outcomes. We also assessed the effect of [...] on an individual's repeat [...] outcomes scores. [...] Linear mixed effects models were fitted with time as a fixed effect and a random effect of subject."			finding, particularly as there was no consistent association with [...] at any other [...]. [...] We would suggest the overall impact would be potentially small as there was no **clinically important impact** on [...] at any age. We acknowledge that [...] may have attenuated to the null any potential detrimental effect of [...] on [...] outcomes, but this would further support that [...] **does not have** permanent consequences for [...]."	cause of the outcome and after adjusting for potential confounders conclude that it is not, given that they only identify one positive association when multiple were assessed and consider it to be by chance.

For peer review only

"To determine if [...] is **associated** with an increased risk of [...] in the general population."	"[...] is **associated** with a **greater risk** of [...] compared with [...], but not a greater risk of death. The relative risk increase is similar across population groups, but the **higher baseline risk** among those [...] **translates into higher absolute risks of** [...] in these groups."	"We calculated odds ratios for each outcome [...] within 14 days of [...] comparing each [...] adjusting for potential **confounders** using logistic regression."	"Based on a priori knowledge, we considered the following variables as potential **confounders** of the relation between [...] and [...]: [...]. All covariates other than sex and ethnicity were updated over time. [...] We initially adjusted for sex and age only, and then fitted an adjusted model using [...]."	"In the 14 days after [...], [...] is associated with the highest odds of [...] (adjusted odds ratio 1.72, 95% confidence interval 1.31 to 2.24) and [...] (2.27, 1.49 to 3.45) of all the [...] investigated. [...] The odds of death within 14 days of [...] were similar to [...] for [...] (0.90, 0.76 to 1.07) and the other [...]."	"We saw minimal differences in the odds ratios for [...]. [...] analyses using multivariable regression and inverse probability treatment weighting approaches [...] were consistent. [...] our study also had greater ability to adjust for detailed characteristics, such as [...], which are likely to have reduced **residual confounding**. "	The elements that point to a causal aim: confounder adjustment by regression models, sensitivity analysis using inverse probability of treatment weighting and discussing residual confounding.
"To evaluate the [...] **safety** of [...],	"In this large cohort study,	"For each comparison and for all outcomes, we	"We considered the following	"Table 3 shows that after	"Randomized controlled trials	Both abstract and main text

For peer review only

in direct comparisons with [...], as used in routine practice."	[...] was associated with a **lower risk** of [...]l and with a **similar risk** of [...] in direct comparisons with [...] as used in routine care."	calculated unadjusted and **propensity score matched** number of events, incidence rates, and hazard ratios with 95% confidence intervals."	covariates as potential **confounders**: [...] To control for imbalances in patient characteristics between cohorts, we calculated exposure **propensity scores** as the predicted probability of receiving the treatment of interest (ie, [...] v each comparator) conditional upon the subjects' baseline covariates using three separate multivariable logistic regression models. All variables were included and no	**propensity score matching**, for [...] primary outcome, the number of events for [...] and the [...] comparator were 91 and 124 respectively (8.9 v 12.8 per 1000 person years; hazard ratio 0.70, 95% confidence interval 0.54 to 0.92) in cohort 1; 94 and 148 (7.5 v 12.4; 0.61, 0.47 to 0.78) in cohort 2; and 77 and 154 (7.3 v 14.4; 0.51, 0.38 to 0.67) in cohort 3."	are the best way to assess drug efficacy [...] On the other hand, strict inclusion and exclusion criteria and rigorous safety monitoring limit the **generalizability** of randomized controlled trial results. Our study [...] allowing better generalizability to routine care [...] provides data from direct comparisons. [...] while we used **propensity score matching** to balance more than 100 baseline characteristics between the groups, **residual**	use causal language and they are explicit to state that the aim is to evaluate safety of the exposure and use causal methods (propensity score matching). They discuss why the design was observational and consider that due to this nature, residual confounding cannot be excluded that may have led to downtown of conclusion which is phrased more

			further selection was conducted. We 1:1 **matched** cohorts on their **propensity score** using a caliper width equal to 0.2 of the standard deviation of the logit of the propensity score.”		**confounding** by some unmeasured characteristic(s) cannot be ruled out. ”	in terms of association.
INCONSISTENT						
“To evaluate the relation between […] and development of […]”	“ […] was associated with an increased risk of […] that was mediated by […]. Systematically addressing […] may be an important public health strategy to	“We calculated the hazard ratios for the relation of […] to the risk of MRSA using Cox proportional hazard models. […] We also calculated the absolute risk difference. […] We performed mediation analyses to examine the extent to which the effect of […] on the risk of […] was through	“We performed a matched cohort study […]matched on age (one year either way), sex, and study entry time (within one year either way). Such comparators were chosen to	“The matched and multivariable adjusted hazard ratios for patients with […] were 1.69 (1.51 to 1.90) for […] and 1.26 (1.12 to 1.40) for […].”	“Our GP practice based dataset could have missed the detection of some inpatient cases of […]; however, these potential non-differential misclassifications would have	

For peer review only

	reduce the incidence of […] among patients wih […].”	[…]. Using marginal structural models we then estimated the natural direct effect […] and the natural indirect effect […] while adjusting for the same confounding variables”	further ensure the comparability […] In the multivariable Cox model we adjusted for […].”		biased our results towards the null, rendering our findings conservative”	
“To quantify the effects of varying […]”	“[…] is associated with a large increase in […] among […] patients. The data from this study suggest that […] rather than […] is more strongly associated with […]”	“For adjusted analysis of time until […] we used Cox proportional hazards models.”	“Adjusted models included […]”	“Each additional […] increased the rate of […] by 70.7% (95% confidence interval 54.6% to 88.4%) before adjustment and increased the hazard of […] by 44.0% (40.8% to 47.2%, P<0.001) after adjusting for covariates.”	“To determine the extent to which strong unobserved confounding might explain the observed association, we included this synthetic confounder in a Cox model. […] As part of a sensitivity analysis, we constructed models that removed potential	

For peer review only

					confounders. […]”	
“To evaluate the long term association between […]”	“Widespread utilisation of […] may be contributing to long term increased risk of […]. The potential for […] should be considered when[…].”	“We conducted Poisson regression analyses using person years as observations.”	“We included several variables as known confounders or effect modifiers in the relation between. […]The final fully adjusted model adjusted for […]”	“After adjustment for covariates, the rate ratio was […] indicating that during the entire period of follow-up the risk of […] was 21% higher during […] than at other times.”	“The registered active […] population is generally representative of the UK population in terms of age, sex, and regional distribution”	
“To investigate the association of […]”	“The shape of the association between […] and […] was determined by […].This finding suggests that the […] may be largely explained by […]”	“We used Cox proportional hazards models to estimate hazard ratios and 95% confidence intervals. We stratified the analysis by age in months and calendar year of the questionnaire cycle.”	“For the main analysis, we used […] measured at baseline to minimize the effect of underlying diseases on mortality […] In multivariable models, we adjusted for potential	“A multivariable adjusted model showed a positive association between […] and all cause mortality, whereas […] showed a U shaped association with all cause mortality. In a	“Our findings remained robust in several sensitivity analyses […] we cannot entirely rule out the possibility of unmeasured or unknown confounding factors that may account for the	

For peer review only

			confounders including […]”	mutually adjusted model including both […] and […] we consistently observed a strong positive association between […] and all cause mortality.”	associations observed in this study.”	
“ To examine the associations of […]”	“This association could be explained by the finding that […] These results emphasise the importance of revisiting […] or establishing specific guidelines for management among […]”	“We performed Cox models with penalised splines”	“In final Cox models with penalised splines, we made adjustments for: […]”	“[A]fter adjustment for confounding factors, the U shaped association with […]”		

For peer review only

" To estimate long term survival, health, and educational/social functioning in patients with […]"	"[…] had no substantial effect on […]"	"We calculated mortality rate ratios and incidence rate ratios as measures of relative risk."	"For each […] patient, we used the Danish Civil Registration System and the DNPR to identify all Danish residents with the same sex and date of birth as the patient who had not tested positive […] and who met the study's inclusion and exclusion criteria […]. From this population, we extracted 10 people at random for each patient. People in the population comparison cohort were assigned the same date of study inclusion as	Patients and members of the comparison cohort were well matched with respect to […] Mortality was not higher among patients in the […] cohort"		

For peer review only

			[…] patients to whom they were matched.”			
“To compare the risk of […]”	“ Although residual confounding cannot be excluded, this finding deserves consideration when […] is used for […]”	“We estimated the crude hazard ratio of […] using Cox proportional hazard regression, and the adjusted hazard ratio was obtained using propensity score matching”	“ We identified potential confounders that were plausibly associated with both […]based on clinical knowledge […] In the context of this study, the propensity score is the probability of receiving […] as opposed to […], given the baseline characteristics. Patients who received […] were matched to patients who […] using a 1:1 nearest neighbor matching algorithm with a	“The crude hazard ratio of death in the unmatched cohort was 1.51 (95% confidence interval 1.22 to 1.85) and the adjusted hazard ratio in the matched cohort was 1.50 (1.14 to 1.96)”	“Comparison of the baseline characteristics in the unmatched cohort provided little evidence of confounding […] it is unlikely that a few additional unmeasured variables can explain a 50% increase in the risk independent of all other confounder and proxies of confounders that are adjusted for in our study.”	

For peer review only

			caliper of 0.2 of the standard deviation of the propensity score on the logit scale. Covariate balance between the two groups was assessed after matching, and we considered an absolute standardized difference less than 0.1 as evidence of balance”			
“To determine whether […] is<br><br>associated with […]”	“[…] was independently associated<br><br>With […]”	“We fitted both a mixed effect logistic regression model (in which the outcome was defined as dichotomous […] and the Prentice, Williams, and Peterson (PWP) model”	“We examined the relation between […] and […] adjusted for …”	“The rate of distinct criteria met per year increased by 24% if a patient had been admitted to hospital (hazard ratio 1.24, 95% confidence interval 1.20 to 1.28) when	“We did a sensitivity analysis using propensity score matching to assess whether the association between […] and […] could be due to unmeasured confounders […]	

For peer review only

				controlled for the other covariates”	Although we adjusted for a range of characteristics of patients, as with any observational study potential exists for unmeasured confounding, which may partly or fully explain the relation between […]”	
“To investigate associations between […]and to analyse the effect of changes […]”	“Risks of […] are inversely associated with […]”	“We used multivariable Cox regression analysis to compare the rates of […] and […].“	“Confounders included in the final models were based on the literature or statistical significance (P<0.10). The full model included […]”	“Compared with […], […] had increased hazard ratios of […]”	“We believe that our findings are widely applicable and provide justification for […] and continuing […]”	

For peer review only

"To estimate the rates of […]"	"In cases of […], approximately […] will become […], of which a third will have […]."	" We present denominators where data for the secondary outcome are missing. We defined the population attributable fraction as (Re–Run)/Re=(RR–1)/RR, calculated using Stata. To test the robustness of our findings, we did a sensitivity analysis."	"We compared the demographic and clinical variables of […]. We used the binomial Wilson score to calculate confidence intervals of single proportions and the Pearson exact method to calculate confidence intervals of risk ratios and medians."	"[…] had a higher risk of […]The population attributable fraction of […] was 47% for […] and 61% for […]"	" Considering these results when counselling potentially exposed […] seems reasonable"	
To perform an expedited assessment of […] risk associated with exposure to […]".	"The results do not imply a markedly increased short term overall risk of […] in […]."	"We used Cox regression to estimate the hazard ratio with 95% confidence intervals for […] associated with […], both for ever use and for the predefined categories of cumulative use"	"Analyses were, however, performed as crude comparisons adjusted only for […] as well as adjusted for […] and the potential	"Overall, exposure to […] showed no association with […] compared with exposure to […] (adjusted hazard ratio 1.09, 95% confidence interval 0.85 to	"This ensured that the estimates were not affected by immortal time bias [..] As all comparisons were performed within users of […], the exposure	

For peer review only

			confounding factors.”	1.41) and no evidence of a dose-response relation”	to […] can reasonably be expected to be a random event, and confounding is thus expected to be limited.”	
“To investigate the risks of […] in […]”	“No increased risk of […] was detected in […], but increased risks of […] were found in this study. Our results suggest that […] risks could be due to […], rather than […].”	“To calculate expected […], we multiplied the person years at risk by corresponding national incidence rates (by 5 year age band and individual calendar year) for the general female population of England and Wales. Standardised incidence ratios were calculated by the comparison of observed values with expected values.”	“We obtained data relating to potential confounding factors such as […]”	“There was no overall increased risk of […] (2578 observed v 2641.2 expected […]; standardised incidence ratio 0.98 (95% confidence interval 0.94 to 1.01); absolute excess risk −2.8 cases per 100 000 person years (95% confidence interval −7.1 to 1.8); table 2)”.	“Given previous inconsistent results, small study size, and lack of information on potential confounders, we undertook a population based linkage study in […]”	
CONSISTENTLY NOT CAUSAL						

For peer review only

"To determine whether […], compared […], is **associated** with an increased risk of […]"	"In this population based cohort study, […] was **associated** with an increased risk of […]. The association was particularly elevated among people using […] for more than five years. **Additional studies**, with long term follow-up, are needed to investigate the **effects** of […] on […]."	"We calculated crude incidence rates of […] and 95% confidence intervals, based on the Poisson distribution, for each exposure group. We used time dependent Cox proportional hazards models to estimate hazard ratios and 95% confidence intervals of […] associated with […] compared with […], using multiple imputation for variables with missing values."	"Potential **confounders**. All models were **adjusted** for the following variables measured at cohort entry: […]. […] as an alternate means of controlling for **confounding**, we repeated the analysis by stratifying the model on tenths of disease risk score. Finally, we repeated the analysis using a **marginal structural** Cox proportional hazards model using **inverse probability of treatment** and censoring	"Compared with […], […] were **associated** with an overall 14% **greater risk of** [..] (1.6 v 1.2 per 1000 person years; hazard ratio 1.14, 95% confidence interval 1.01 to 1.29)."	"We introduced a one year exposure lag period to account for a minimum latency time window and to minimize **reverse causality**. […]The association between […] and […] is **biologically plausible**. […] although we were able to adjust for several important **confounders**, this study lacked information on other potential **confounders** such as […] In this large, population based study, […] was associated with an elevated risk of […] overall, along with	In the abstract they only describe associations but in the full text their interest points to a causal aim given the different methods applied to adjust for confounding and reverse causality. They also consider elements as biologically plausibility and duration response relation. Residual confounding seems to be a concern because they

			weighting—a method designed to adjust for time dependent **confounding** associated with time varying exposures”		evidence of a **duration-response relation.**”	lacked information on relevant cofounders which could lead to down tone of the conclusions.
“To determine whether […] and […] are **associated** with an **increased risk of** […] in adults with […].”	“Compared with […], […], and […], **might be associated** with an increased risk of […] in adults with […].”	“For each exposure category we calculated crude incidence rates of […] with 95% confidence intervals, based on the Poisson distribution. Time dependent Cox proportional hazards models were used to estimate hazard ratios and 95% confidence intervals of […] associated with […] and […], separately, when compared with […].”	“The models were **adjusted** for the potential **confounders** measured at cohort entry: […] To minimise potential **confounding** by indication, we compared […]. […] we fit a **marginal structural model** to investigate the impact of potential time dependent	“Compared with […], […] was **associated** with a 77% increase in the hazards of […] (hazard ratio 1.77, 95% confidence interval 1.04 to 3.01).”	“Finally, we excluded those with less than one year of follow-up after cohort entry, to allow for a sufficient latency period and to minimise **reverse causality**. […] To assess possible **duration-response relations**, we investigated the association between	The abstract only refers to association but the full text mentions adjusting for confounders and ways to minimise reverse causality. They also describe its biological plausibility. There is a concern for residual confounding

For peer review only

			**confounding** using **inverse probability of treatment** and censoring weighting."		cumulative duration of [...] on the risk of [...]. An association between [...] and incidence of [...] is **biologically plausible.** [...] as with all observational studies, **residual confounding** is possible. We conducted several sensitivity and ancillary analyses specifically designed to assess the potential impact of **residual** confounding."	due to the observational nature rather than missing information on particular relevant confounders.
"To examine the **association**	"[...] **could increase the risk of** [...].	"[...] was categorised by percentiles (<10, 10-20, 20-50, 50-80, 80-90, ≥90).	"Characteristics that might influence the risk	"[...] was significantly associated with	"[...] the mechanism that might be	The abstract only considers associations

For peer review only

between […] and […] risk of […].”	However, confirmation of these findings are warranted, preferably in an intervention setting”	[…] With these same categories of exposure, the association between […] and […] was examined by Cox regression. We used […] age from birth up to May 2016 as the underlying timescale censoring if death or emigration from Denmark occurred (1217 events).”	of [….] were identified a priori and included as potential **confounders** in our **adjusted** analysis. In model 1, we adjusted for: […].In model 2, additional adjustments were made for […]”	**increased risk of** […] in both unadjusted and covariate **adjusted** analyses (table 3). Compared with […], offspring of those with […] had double the risk of […] during follow-up (hazard ratio 2.00 (95% confidence interval 1.02 to 4.00)). Risk of […] was positively **associated** with […]: the **association** was significant (Ptrend=0.016) and increased monotonically. Only minor differences were observed between the unadjusted and	responsible for this **effect** is not known, but could include […]. […] the role of unmeasured or unidentified confounders can never be fully excluded in observational studies.”	but the full text mentions confounder adjustments and discusses potential mechanism (biological plausibility). Concern of residual confounding is due to the observational nature of the study.

				covariate **adjusted** analyses."		
"To ascertain compliance **rates** with [...]; to identify features **associated** with non-compliance; to **rank** [...] by compliance; and to build a tool for live ongoing audit of compliance."	"Compliance with [...] has been poor, with half of all [...] non-compliant. [...] commonly contain inconsistencies that might prevent even [...] assessing compliance. Accessible and timely information on the compliance status of [...] and [...] may help to improve reporting **rates**."	"We constructed a logistic regression model with all these **explanatory** variables, as they were selected prospectively on the basis of clinical and methodological interest."	"**Explanatory** variables. We created variables for a range of features of each [...], selected prospectively on the basis of clinical and methodological interest."	"In the **adjusted** multivariable analysis, [...] with a [...] were **significantly more likely to** [...] (adjusted odds ratio 23.3, 95% confidence interval 19.2 to 28.2); as were [...] (18.4, 15.3 to 22.1)."	--	Although adjusted estimates are present, both abstract and full text limit to describes associations, rates and ranks. No causal language is used.

For peer review only

"To assess **how often** older adults […] were […], and to identify markers of […]."	"**One in seven** older adults […] were […]. **More than half of** […] occurred in patients with […]. More attention is needed to reduce potentially harmful […] as older adults […]."	"We did multivariable mixed effect logistic regression analyses to determine **associations** between the outcome of […] and primary **predictors** of […]."	"Our primary **predictor** variables were […]. **Adjusted** analyses included the covariates noted above, a random effect term to account for clustering by hospital, and an interaction term to account for the relation between […] and […]."	"A total of 2074 (14%) patients were […]; 1293 (9%) were […] and 300 (2%) were […]. Additionally, 628 (4%) patients were […]. […] Patients with […] had a 25% (95% confidence interval 23% to 78%) probability of […]."	--	The abstract indicates that the aim is to assess the frequency of a condition and that is reflected in the main text. No causal language used.
"To describe **trends** in the **rate** and daily dose of […] used among […] from 2007 to 2016."	"[…] **rates** were high during the study period of 2007-16, with the **highest rates** in […] versus […] and […]. […] and average daily dose have not	"Endpoints were defined at the person quarter level. We used logistic regression to model the **proportion** of the population […] each quarter. The average […] per person day by quarter was modelled by a generalized linear model with negative binomial	"All analyses were stratified by beneficiary category including commercially insured, aged Medicare, and disabled Medicare	"Averaged across the entire study period, 51.5% of disabled Medicare beneficiaries […] per year (n=1 128 088), compared with 14.3% (n=18 721 915) of	--	The abstract indicates that the aim is to describe the frequency of a condition which is reflected in the main text. No

For peer review only

	**substantially declined** from their peaks, despite increased attention to […] and awareness of their **risks**.”	family and log link. The dependent variable was the total […] per person in the quarter, with an exposure variable representing the number of days of insurance coverage for each person included to **standardize** daily […].”	(beneficiaries with Medicare coverage who were under age 65 years)”	commercial beneficiaries and 25.7% (n=3 847 676) of aged Medicare beneficiaries.”		causal language used.
“To **describe** […] related mortality in the United States during 1999-2016 by age group, sex, race, cause of […], and geographic region.”	“Mortality due to […] has been **increasing** in the US since 2009. Driven by deaths due to […], people aged 25-34 have experienced the **greatest relative increase** in mortality. White Americans,	“Our primary aim was to describe temporal trends in death rates attributable to […] and […] as the primary or underlying cause of death for adults in the USA.[…] We then evaluated trends in death rates using the National Cancer Institute’s Joinpoint program. This enabled us to identify if there were years in the study period where the rate of change in mortality was statistically	“We adjusted rates for age—that is, age specific mortality was weighted according to the age distribution in a standard year (2000). We also sought to describe how these trends differed based on demographic subgroups; age, sex, race (Asian or	“During the study period, a total of 460 760 deaths were attributed to […] (20 661 in 1999 and 34 174 in 2016) and 136 442 to […] (5112 in 1999 and 11 073 in 2016) (table 1). Men had a higher **burden** of age **adjusted** mortality due to […] compared	“[…] though we have detected worsening mortality since 2009, the precise reasons for this trend and the geographic heterogeneity in our analysis require further study. For example, we identify the states most at risk, but granular data are	The abstract indicates that the aim is to describe the frequency of a condition which is reflected in the main text. No causal language used.

	Native Americans, and Hispanic Americans experienced the **greatest increase** in deaths from [...]. Mortality due to [...] is improving in Maryland but worst in Kentucky, New Mexico, and Arkansas. The rapid increase in death rates among young people due to [...] highlight new challenges for optimal care of patients with [...]."	significantly different. The program uses a piecewise linear regression approach to determine whether rates over time are best described by a straight line (0 joinpoints) or by multiple linear segments (≥1 joinpoints)"	Pacific Islander, Native American (designated as "American Indian" in the census database) or Alaska Native, black or African American, and white American), Hispanic ethnicity, and geographic area of residence."	with women by a 2:1 ratio and a higher **burden** of mortality due to [...] by a nearly 4:1 ratio."	needed to determine the root causes"	

For peer review

"To examine whether […] are **associated** with an increased risk of […] after […]."	"Women with […], especially […], may be at **higher risk of** […]. If these findings are replicated elsewhere, a massive amount of data exists that could aid in identifying women at **higher risk of** […] and that could be conveyed to them or their healthcare providers."	"The main model assessed the primary […] composite outcome, as well as the individual outcomes of […], in relation to each […] for the screened cohort, with censoring at a woman's death or arrival at the end of the study period of 31 March 2016, allowing for a maximum follow-up of 22 years. We did time to event analyses using multivariable Cox regression models,to derive a hazard ratio and 95% confidence interval for each study outcome."	"Hazard ratios were **adjusted** for variables chosen a priori, based on the existing literature, including: […]"	"A total of 6209 women developed the primary […] composite outcome, which was typically about 1.2 to 1.3 times **more likely** to occur in a […], even after **adjustment** for other covariates"	"Potential **confounders** between […] and the risk of […], including […] were each accounted for in the models. Nevertheless, about 10% of […] lacked information on […], and […] and […] were entirely unknown."	The abstract indicates that the aim is to identify the association between an exposure and an outcome. They consider residual confounding due to lack of information on relevant confounders.
"To examine the **association** between […] and the risk of […]	"Among […], increasingly worse […] was **associated** with	"Using generalised linear models with a robust sandwich estimator, we estimated risk ratios for	"Analyses were **adjusted** for […]"	"In analyses based on […] levels, the **adjusted** risk	"It is also possible that the previously demonstrated	The abstract indicates that the aim is to identify the

according to levels of [...].”	a **progressively increased** risk of [...]. Even with [...] within target levels recommended by guidelines [...], the risk of [...] was increased more than twofold. The risk of [...] was not statistically significantly increased at any of the [...] levels examined; the study had limited statistical power for this outcome and was based on [...] only.”	[...], comparing [...] according to levels of [...] with [...]. To take into account possible dependence from repeated [...], we constructed models with [...] as a cluster variable. [...] were assumed to follow a poisson distribution, and we estimated risk ratios using a log link function”		ratios for [...] were 2.17 (95% confidence interval 1.37 to 3.42) for [...], 3.17 (2.45 to 4.11) for [...], 2.79 (1.90 to 4.12) for [...], and 6.23 (4.32 to 9.00) for [...] versus [...]. The corresponding **adjusted** risk differences were 17 (95% confidence interval 5 to 36), 32 (21 to 46), 26 (13 to 46), and 77 (49 to 118) cases per 1000 [...].”	**association** [...] has resulted in increased clinical surveillance for defects among [...]. [...] health registers do not record data on [...] and hence we could not account for these factors.”	association between an exposure and an outcome. They consider residual confounding due to lack of information on relevant confounders.

For peer review only

"To examine the **association** between risk factor burdens—categorized as optimal, borderline, or elevated—and the lifetime risk of [...]."	"Regardless of index ages at 55, 65, or 75 years, an optimal **risk factor** profile was associated with a lifetime risk of [...] of about one in five; this risk rose to more than one in three in individuals with at least one elevated risk factor."	"We calculated the lifetime risks for the first incident [...] from index ages 55, 65, and 75 yearsup to age 95 years. [...] we used a modified Kaplan-Meier estimator with age as the time scale, accounting for the competing risk of death to compute the lifetime cumulative risk of [...] and associated 95% confidence intervals"	"We computed lifetime **risk in subgroups** of participants according to their risk profile at a specified index age (optimal, borderline, and elevated), for each **risk factor** separately and for the combination of **risk factors**. [...] we fitted a multivariable Fine and Gray model, **adjusted** for competing risk of death to **predict** the lifetime risk of [...]."	"The associated lifetime risk of [...] was lowest if the risk factor profile was optimal. The lifetime risk of [...] increased gradually as the risk factor profile changed from optimal to borderline and elevated at each index age."	"Our observational study design limits the ability to establish **causal pathways**, and only **associations** between **risk factor** profiles and lifetime risk of [...] can be concluded from our study."	The abstract and main text indicate that the study mainly aims at identifying associations. There is a concern for residual confounding due to the observational nature rather than missing information on particular relevant confounders.

For peer review only

“To compare **rates** of […] for patients […], with patients […].”	“[…] was **associated** with lower […] **rates** compared with […].”	“We used a Cox proportional hazards regression model, adjusting for (…), to estimate hazard ratios and 95% confidence intervals for […] comparing […] with […]. To summarize switchback estimates across […], we conducted inverse variance weighted random effects meta-analyses”	“[…] **adjusting** for basic demographics (age, sex, and calendar year)”	“Figure 5 shows that in the adjusted analysis, the […] rates remained consistently lower among […] than […]. The magnitude of this effect was largest for […] (hazard ratio 0.52, 95% confidence interval 0.43 to 0.63) and smallest for […] (0.86, 0.77 to 0.97). The pooled hazard ratio across […] suggested that […] was associated with a 28% lower rate of […] compared with b[…] (0.72, 0.64 to 0.81).”	“[…] our results indicate that […] may in part be **driven by** […].”	Even though causal language is not used and both the abstract and main text mainly describe associations, their conclusion at the end of the text suggests a causal relationship.

For peer review only

"To assess whether [...] is **associated** with the incidence of [...] in patients with [...]."	"In this first population based study, [...] was **associated** with an increased risk of [...]. Although these findings need to be replicated, physicians should be aware of this possible association."	"We calculated crude incidence rates of [...] with 95% confidence intervals based on the Poisson distribution for the entire cohort and for each exposure group. For all analyses, we used time dependent Cox proportional hazards models to estimate hazard ratios and 95% confidence intervals for [...] associated with [...] compared with [...]. We also calculated the **number needed to harm** for patients followed over a two year and four year period by using methods accounting for varying patient follow-up times."	"The models were **adjusted for** the following potential **confounders** measured at cohort entry: [...]"	"Compared with [...], [...] was **associated** with a 75% **increase in risk** of [...] (53.4 v 34.5 per 100 000 per year; hazard ratio 1.75, 95% confidence interval 1.22 to 2.49). The **number needed to harm** corresponded to 2291 patients followed over a two year period and 1177 over a four year period."	"[...] as with all observational studies, **residual confounding** from unknown or unmeasured variables remains possible. However, on the basis of the rule out method, a hypothetical confounder would need to be strongly associated with both the exposure (odds ratio >4.7) and the outcome (relative risk >5.0) to move the point estimate towards the null."	The abstract mainly describes associations but in the main text they describe how their estimates were adjusted for potential confounders and they estimate numbers needed to harm. Also they consider residual confounding and suggest that only a strong unmeasured confounder will remove the association observed. All of these, point

						to a causal analysis.
“To assess the prospective **associations** between [...] and risk of [...].	“In this large prospective study, a 10% increase in the proportion of [...] associated with a **significant increase** of greater than10% in risks of [...]. Further studies are needed to better understand the relative effect of the various dimensions of [...] in these associations.”	“We used Cox proportional hazards models with age as the primary timescale to evaluate the association between [...] and incidence of [...]. In these models. We estimated hazard ratios and 95% confidence intervals with the lowest quarter as the reference category.”	“Models were **adjusted** for [...] we made additional adjustments [...]. In addition we did **mediation** analyses [...]”	“In model 1, [..]. was associated with increased risks of overall cancer (hazard ratio for a 10 point increment in the proportion of [...] 1.12 (95% confidence interval 1.06 to 1.18), P<0.001) and [...] (1.11 (1.02 to 1.22), P=0.02). .”	“Lastly, although we included a large range of **confounding factors** in the analyses, the hypothesis of **residual confounding** resulting from unmeasured factors (...) cannot be entirely excluded owing to the observational design of this study”	The abstract mainly describes associations but in the main text they describe how their estimates were adjusted for potential confounders and they use mediation analysis which suggest a causal aim. They also consider residual confounding due to the observational nature of the study.

"To assess the **association** between [...] and all cause mortality in [...] with [...]."	"Giving [...] to [...] with [...] was **associated** with an increased rate of [...] but a paradoxical lowered rate of all cause mortality. Careful consideration should be given before [...]. There remains an urgent need for adequately powered randomised trials in this population to explore these findings and to provide clarity on correct clinical management."	"We calculated the incidence of [...] and all cause mortality per 100 person years of follow-up. We generated Kaplan-Meier survival curves for the outcomes of interest grouped by [...] status. Cox proportion regression were reported as **adjusted** hazard ratios with 95% confidence intervals. Because all baseline characteristics were balanced in the **propensity matched** model, [...] was the only independent variable in our Cox regression model."	"We used propensity score matching with demographic and clinical variables to adjust for potential confounding from imbalances in clinical characteristics between patients receiving and not receiving [...]. Demographic variables were [...]. Clinical variables were [...]. Patients who received anticoagulation were matched in a 1:1 ratio"	"The crude rates for [...] and [...] were 4.6 and 1.2 after [...], and 1.5 and 0.4 in patients who [....] per 100 person years, respectively. In the Cox proportion regression models, the hazard ratios for [...], [...] and all cause mortality for patients [...] were 2.60 (95% confidence interval 2.00 to 3.38), 2.42 (1.44 to 4.05), and 0.82 (0.74 to 0.91) compared with those who [...]."	"The study population was derived from real world evidence with the inherent limitations of diagnostic coding and case ascertainment Despite well **matched** groups after **propensity score matching**, we cannot exclude that the reported associations were **confounded** by indication"	The abstract limits to describe associations and rates of the condition but the main text suggest a causal aim as they use propensity score matching to control for confounding.

"To assess the **temporal change** in [...] and to identify [...] with the greatest increase in use."	"Total [...] use has increased markedly over time, in both sexes, and across all age groups [...]. Of the patients who [...], the **proportion** who had [...] increased significantly over time."	"For comparison across years, we **standardised** crude rates to the mid-2015 UK population. Rates were calculated for [...]. Joinpoint regression was used to model the temporal changes in age and sex **standardised** rates"	"For each year we calculated the total number [...], **stratified** by age and sex. We calculated total person years of observation in each age and sex stratum for each year."	"The age and sex **adjusted** rate of [...] increased from 14 869 tests per 10 000 person years in 2000/1 to 49 267 in 2015/16 (table 1, fig 1), an annual increase of 8.5% (95% confidence interval 7.6% to 9.3%). [...] The slope of the trend line changed significantly at two points: 2004/5 (P<0.001) and 2008/9 (P=0.004) (fig 1)."	"We analysed the temporal change in [...] from UK primary care. The total use of tests increased markedly over time, even after adjustment for population growth"	The abstract and full text state that they aim to quantify changes of a condition over time. It is a descriptive study that does not use causal language accordingly.
To investigate the **association** of [...] with disease specific incidence and mortality and	"[...] was **associated** with a range of health outcomes and	"[...] we investigated the associations of [...] with cause specific incidence and mortality over follow-up with Cox proportional	"We treated [...] as potential **confounders**. For Cox proportional hazard analyses,	"As shown in figure 1, in both men and women, [...] was **associated** with a	"To minimise the potential contribution of **reverse causality** to the findings,	The abstract mentions associations and suggests that the aim is

whether [...] enhances the **prediction** ability of an established office based **risk score**.""	improved **prediction** of an office based **risk score**. Further work on the use of [...] in risk scores or risk screening is needed to establish its potential clinical utility."	hazard models. We reported the results as hazard ratios together with 95% confidence intervals."	we ran four models that included an increasing number of covariates: model 0 (minimally adjusted) included [...]"	higher hazard for all cause mortality and incidence of and mortality from [...] in model 0. The **associations** were similar after **adjustment** for [...] in model 1; after further adjustment, the magnitude of associations were slightly attenuated in models 2, 3, and 4"	we did a landmark analysis excluding events occurring within the two years after recruitment in model 4 (landmark analysis). This landmark analysis was adjusted as in model 3. [...] may, therefore, be a useful method of **identifying** people with [...] who are at high risk of a wide range of diseases. [...] **Reverse causality** is possible in any observational study. [...] Similarly, **residual confounding** is always possible and the	prediction. In the main text, the authors conclude that the exposure of interest enhances prediction and identification of patients with risk of certain diseases. However, there is use of causal language, including confounder adjustment and discussing reverse causality and residual confounding. They note that their goal is to do prediction and that

For peer review only

						associations observed may not imply **causality**. However, given that we are largely interested in **prediction** and identification of people at increased risk, and not seeking to make strong causal inferences, **reverse causality** is not a major limitation. ”	reverse e causality is not a major limitation but still adjust for it.
To **externally validate** four commonly used rules in […] for […].“”	“Application of the […] rules can lead to a wide variation in […] among patients with […], **resulting** in many unnecessary […] findings.	“The sensitivity, specificity, and proportion of patients […] (with 95% confidence intervals) were assessed for each of the four decision rules. […] The Cochran’s Q test was used to directly compare the sensitivities and specificities between the	--	“The sensitivity for identifying patients with […] ranged from 72.5% for the […] criteria to 98.8% for the […] rule (table 4; appendix 3). […] The […] criteria would	--	Both the abstract and the full text state that they aim to validate four decision rules for a particular condition. They

For peer review only

	Until an existing decision rule has been updated, any of the four rules can be used for patients presenting [...] at the emergency department. Use of the [...] rule is recommended because it **leads to** a substantial reduction in [..] while missing few potential [...].”	four decision rules [...]. Net proportional benefit has been proposed to incorporate such weighting in calculation of clinical usefulness of decision rules. For each rule, we expressed the net proportional benefit using theformula: (true positives/total number) − weight × (false positives/total number). ”		have missed 11 of 74 patients with [...] (appendix 4). The CHIP criteria would have missed two patients with [...], who both had [...]. The specificity for identifying [...] was lowest for the [...] rule (4.4%) and highest for the [...] criteria (60.9%). [...] The sensitivity and specificity differed significantly between all the rules (Cochran’s Q P<0.001). ”		compared the tests in terms of sensibility or specificity and concluded that the tests are similar and recommended the use of a particular one given that it can help avoid false negatives. The wording ’resulting’ and ‘leads to’ are in fact to discuss the potential for false positives/ false negatives rather than a casual claim.
“To develop and **validate** a set of	“The **prediction** models reliably	“The **association** between predictor variables and [...]	“In a published systematic	“Bootstrap resampling	--	The abstract and main text

For peer review only - http://bmjopen.bmj.com/site/about/guidelines.xhtml

practical **prediction tools** that reliably estimate the outcome of [...].”	estimate the outcome of patients who were managed in various settings for [...]. The **predictor** items are readily derived at hospital admission. The web based [...] **prognostic** calculator [...] and the related app could be adjunctive tools to support management of patients.”	was analysed by fitting proportional odds logistic regression models **adjusting** for the fixed effect of study. Prognostic strength was quantified as odds ratios with 95% confidence intervals. The relative importance of each **predictor** in the models was estimated with partial R2 statistic, which estimates the independent **contribution** of the **predictor** to the variance of the outcome”	review, we identified relevant **predictors** of outcome in patients [...]. Based on the results of this published review, we selected the following **predictor** variables that are assessable early at hospital admission and are consistently associated with outcomes for inclusion in the **prediction** models: [...]”	showed negligible model optimism. The models had **internally validated AUCs** between 0.77 and 0.83. There was no significant lack of fit (goodness of fit P≥0.2 in all models). Cross validated performance was variable across studies [...]. The partial R2 values ranged between 4% and 46%, and the pooled AUC values were between 0.74 and 0.77”		state that the goal of the study is to validate a tool. Consistent with the prediction aim, no causal language is used.
“To prospectively **validate** [...] to triage patients	“In a population of patients referred for	“To examine model performance, we constructed a logistic	--	“The triaging strategy [...] predicted true	--	The abstract and main text describe that

For peer review only

with [...] in routine clinical practice.”	[...], this new triaging approach accurately **classified** [...] for most, with half the utilisation of ABPM compared with usual care. This triaging strategy can therefore be recommended for diagnosis or management of [...] in patients where [...] is being considered, particularly in settings with limited resources.”	regression model with true [...] as the dependant outcome variable and classification using [...] as the independent predictor variable. From this model we estimated the **area under the receiver operating characteristic (AUROC) curve** statistic.”		[...] (true positives 66%, 95% confidence interval 63% to 69%; true negatives 24%, 22% to 27%) with a low error rate (false positives 8%, 6% to 10%; false negatives 2%, 1% to 3%) (table 2). The triaging strategy resulted in 49% (46% to 52%) being referred for [...] and the remainder managed on the basis of their clinic measurements.”		the aim is to validate a triage tool and assessed its performance compared to the standard of reference. As the aim is prediction, no causal language is used accordingly.

For peer review only

For peer review only

BMJ Open

Consistency of causal claims in observational studies: a review of papers published in a top general medical journal

Journal:	*BMJ Open*
Manuscript ID	bmjopen-2020-043339.R2
Article Type:	Original research
Date Submitted by the Author:	13-Apr-2021
Complete List of Authors:	Olarte Parra, Camila; Ghent University, Applied Mathematics, Computer Science and Statistics; University of Amsterdam, Clinical Epidemiology, Biostatistics and Bioinformatics<br>Bertizzolo, Lorenzo; INSERM, U 1153, Equipe Methods; University of Amsterdam, Clinical Epidemiology, Biostatistics and Bioinformatics<br>Schroter, Sara; BMJ, Editorial<br>Dechartres, Agnes; Hopitaux Universitaires Pitie Salpetriere-Charles Foix, Departement biostatistique sante publique et information medicale<br>Goetghebeur, Els; Ghent University, Applied Mathematics, Computer Science and Statistics
**Primary Subject Heading**:	Research methods
Secondary Subject Heading:	Epidemiology
Keywords:	STATISTICS & RESEARCH METHODS, EPIDEMIOLOGY, EDUCATION & TRAINING (see Medical Education & Training)

SCHOLARONE™
Manuscripts

I, the Submitting Author has the right to grant and does grant on behalf of all authors of the Work (as defined in the below author licence), an exclusive licence and/or a non-exclusive licence for contributions from authors who are: i) UK Crown employees; ii) where BMJ has agreed a CC-BY licence shall apply, and/or iii) in accordance with the terms applicable for US Federal Government officers or employees acting as part of their official duties; on a worldwide, perpetual, irrevocable, royalty-free basis to BMJ Publishing Group Ltd ("BMJ") its licensees and where the relevant Journal is co-owned by BMJ to the co-owners of the Journal, to publish the Work in this journal and any other BMJ products and to exploit all rights, as set out in our licence.

The Submitting Author accepts and understands that any supply made under these terms is made by BMJ to the Submitting Author unless you are acting as an employee on behalf of your employer or a postgraduate student of an affiliated institution which is paying any applicable article publishing charge ("APC") for Open Access articles. Where the Submitting Author wishes to make the Work available on an Open Access basis (and intends to pay the relevant APC), the terms of reuse of such Open Access shall be governed by a Creative Commons licence – details of these licences and which Creative Commons licence will apply to this Work are set out in our licence referred to above.

Other than as permitted in any relevant BMJ Author's Self Archiving Policies, I confirm this Work has not been accepted for publication elsewhere, is not being considered for publication elsewhere and does not duplicate material already published. I confirm all authors consent to publication of this Work and authorise the granting of this licence.

For peer review only

Consistency of causal claims in observational studies: a review of papers published in a top general medical journal

Camila Olarte Parra[1,2], Lorenzo Bertizzolo[2,3], Sara Schroter[4], Agnès Dechartres[5], Els Goetghebeur[1]

1. Department of Applied Mathematics, Computer Science and Statistics, Ghent University, Ghent, Belgium

2. Department of Clinical Epidemiology, Biostatistics and Bioinformatics, Academisch Medisch Centrum, Amsterdam, The Netherlands

3. INSERM, U1153 Epidemiology and Biostatistics Sorbonne Paris Cité Research Center (CRESS), Methods of therapeutic evaluation of chronic diseases Team (METHODS), Paris, F-75004 France; Paris Descartes University, Sorbonne Paris Cité, France.

4. Editorial, The BMJ, London, United Kingdom

5. Sorbonne Université, Institut National de la Santé et de la Recherche Médicale (INSERM), Institut Pierre Louis d'Epidémiologie et de Santé Publique, AP-HP. Sorbonne Université, Hôpital Pitié Salpêtrière, Département de Santé Publique, F75013, Paris, France

Correspondence to: Camila Olarte Parra
Department of Applied Mathematics, Computer Science and Statistics
Ghent University, Ghent, Belgium
Campus Sterre, S9, Krijgslaan 281, 9000 Ghent, Belgium
Email: camila.olarteparra@ugent.be, colartep@gmail.com

For peer review only

Abstract

Objective: To evaluate the consistency of causal statements in observational studies published in The BMJ.

Design: Review of observational studies published in a top general medical journal.

Data source: Cohort and other longitudinal studies describing an exposure-outcome relationship published in The BMJ in 2018. We also had access to the submitted papers and reviewer reports.

Main outcome measures: Proportion of published research papers with 'inconsistent' use of causal language. Papers where language was consistently causal or non-causal were classified as 'consistently causal' or 'consistently not causal', respectively. For the 'inconsistent' papers, we then compared the published and submitted version.

Results: Of 151 published research papers, 60 described eligible studies. Of these 60, we classified the causal language used as 'consistently causal' (48%), 'inconsistent' (20%) and 'consistently not causal'(32%). Eleven out of 12 (92%) of the 'Inconsistent' papers were already inconsistent on submission. The inconsistencies found in both submitted and published versions was mainly due to mismatches between objectives and conclusions. One section might be carefully phrased in terms of association while the other presented causal language. When identifying only an association, some authors jumped to recommending acting on the findings as if motivated by the evidence presented.

Conclusion: Further guidance is necessary for authors on what constitutes a causal statement and how to justify or discuss assumptions involved. Based on screening these papers, we provide a list of expressions beyond the obvious 'cause' word which may inspire a useful more comprehensive compendium on causal language.

Strengths and limitations of this study

- By evaluating published observational studies in a top general medical journal, we provided relevant examples of (ambiguous) causal statements.
- We focused on the abstract where clear messages are especially important, as many readers start by screening the abstract of the study.
- Comparing the submitted and published versions of the abstract allowed us to identify whether any causal claims were made or not as a result of the peer review process.
- The focus on the use of causal language rather than the specific methods avoided discussion on the validity of underlying assumptions justifying causal inference in the setting studied.
- Assessing observational studies from a single journal allowed us to flag the inconsistent use of causal claims in this context but not to estimate its prevalence more generally.

For peer review only

Introduction

Many researchers remain tempted to draw causal conclusions from observational data despite acknowledging that mere association is not causation because causal inference is the ultimate goal of most clinical and public health research (1, 2). Gold-standard answers are typically sought through randomized controlled trials (RCTs). The unique ability of RCTs to avoid confounding bias (3) has led to demands that empirical research must be drawn from randomized studies to justify causal statements (4-6). RCTs are mainly used to assess the effect of a treatment or intervention but are not easily adapted to evaluate prognostic or risk factors rather than interventions.

There are however good reasons to look beyond RCTs for evidence on treatment effects. In many settings, RCTs are not feasible, ethical or timely and thus observational data are all that is available for some time, as in the recent COVID-19 crisis. Furthermore, observational studies typically involve broader real-world contexts than RCTs, where the costs and risks of experimentation suggest studying high risk patients without major comorbidities (7). This selection challenges generalization to the target population. Highly selected populations with a usually short follow-up, render RCTs inappropriate to evaluate (long-term) unintended side effects. Trials further suffer from treatment non-compliance which complicates analysis, as treatment-specific populations lose the benefit of randomization. Recent ICH E9 guidelines therefore emphasize the importance of causal estimands beyond intention-to-treat, such as per-protocol and as-treated analysis (8, 9).

Deliberately avoiding causal statements on a hoped-for causal answer brings ambiguity and contrived reporting (10, 11). Instead, authors should openly discuss the likely distance in meaning and magnitude between the data based measure they are able to estimate and the desired targeted causal effect. Arguments would consider study design with additional assumptions in context (12). Owing to decades of progress in statistical science (involving potential outcomes, directed acyclic graphs, propensity scores and more) (13), this allows for results, often unreachable by randomized trials, with a justified causal interpretation (14).

In 2010, Cofield et al (5) assessed the use of causal language in observational studies in nutrition but deemed causal language inappropriate for all observational studies. In 2017, Adams et al. (15) also considered that only RCTs allowed for causal inference, in their study assessing how people understand causal expressions in the news. From a different angle, Haber et al (16) examined whether the tone and strength of causal claims made in a given paper matched the language describing the findings in social media. Not surprisingly they found stronger causal statements in the media in half of the cases, emphasizing the importance of clear scientific messages.

To promote this, Lederer et al (17) recently published a guide for authors and editors on how to report causal studies in Respiratory, Sleep and Critical Care Journals. Rather than circumventing the problem by asking to avoid causal language, they provide key elements that ensure valid causal claims (18). Besides briefly explaining causal inference, they provide a definition of a confounder, outline how to identify confounding through so-called directed acyclic graphs and discuss how p-values are often misinterpreted and how their value does not reflect the magnitude, direction or clinical importance of a given association. All these elements empower their target audience to critically assess observational studies.

To find out whether and how statements in study reports present confusing use of causal language (or lack thereof), we examined research papers concerned with exposures and outcomes published in The

For peer review only

BMJ in 2018. Our focus was on the causal message The BMJ readers receive from these papers, particularly from the abstract. We evaluate the consistency of causal statements in the published abstracts of observational studies, whether this consistency was a reflection of the full text and if any a priori changes had been made as a result of the peer review process.

Methods

Sampling and inclusion criteria

COP identified all original research articles published in The BMJ in 2018 described as either cohort or longitudinal studies in the study design section of the abstract. The eligible studies were identified by statements in this section of the abstract such as “cohort”, “longitudinal” or “registry-based”. Those identified as “observational” were included if they suggested a period of follow-up rather than being cross-sectional. Articles described as case-cohorts were excluded as their interpretation and analysis differs from other studies with follow-up assessing the exposure-outcome relationship.

Assessment of published abstracts

Two reviewers (COP, LB) independently screened the published abstracts of the eligible papers. For the text included under each of the subheadings in the abstract (objective, design, setting, participants, outcome, results, conclusion), the reviewers assessed whether there was an (implicit) causal (cl)aim using a yes/no/unclear response. After assessing each separate subheading, each reviewer then gave an overall assessment of the main claims in the paper’s abstract as either ‘consistently causal’, ‘inconsistent’ or ‘consistently not causal’. After the independent assessments, the overall rating of the abstract was compared between both reviewers; where there was disagreement, a third reviewer (EG) was consulted and a consensus reached.

Assessment of published full text

We further evaluated the full published text of all eligible papers to identify the statistical methods applied and any further causal claims. In particular, we looked for statements that would support or undermine a causal aim, including confounding adjustment, discussing residual confounding, exchangeability and issues of transportability. We randomly divided the papers between the two reviewers (COP, LB) for this assessment. For each paper, we extracted statements where authors described the statistical method and method for confounding adjustment, if any. We then extracted the sentences summarizing the results and conclusions to highlight any causal claims.

Assessment of initially submitted abstract version

As the focus of this paper to highlight ambiguous use of causal language, we further assessed those articles judged as ‘inconsistent’ to see if there were changes introduced to the manuscript between submission and publication, leading to this inconsistent use of causal language. For this subset, we obtained the submitted version of the manuscripts and the associated peer reviewers’ comments from The BMJ’s manuscript tracking system. We then compared the published version with the first submitted version of the abstract to identify whether the same wording related to causal claims appeared in the submitted version and whether changes occurred as a result of comments from peer reviewers and editors, as indicated in the corresponding peer review reports.

The same reviewers (COP, LB) independently evaluated the submitted versions of the abstracts. The reviewers assessed whether the content under each subheading of the submitted abstract differed

For peer review only

from the published version. Where there were discrepancies between versions, each reviewer indicated the presence of a causal claim as yes/no/unclear for each abstract subheading (title, objective, design, setting, participants, outcome, results, conclusion) and made an overall assessment of the submitted abstract as either 'consistently causal', 'inconsistent' or 'consistently not causal'. As before, the assessments were compared and, in cases of disagreement, a third reviewer (EG) was consulted and consensus reached.

Patient and public involvement

Patients were not involved in the design, analysis or interpretation of the study. Patients were not participants in this study; it was a methodological study (research on research). Patients' opinions of causal statements and the use of ambiguous language in research papers is important and further work in this area partnered by patients is important.

Results

Assessment of published abstracts

In 2018, 151 research papers were published in The BMJ, of which 60 (40%) were eligible for inclusion in our study. We identified 29 studies (48%) reporting studies using causal language consistently. A further twelve (20%) studies were considered inconsistent mainly because the objective stated the evaluation of an association while the conclusion presented a causal finding (9/12) or the opposite (3/12). Finally, there were papers that described studies aiming for prediction or reporting associations without (implicitly) suggesting that they had a causal nature that were considered consistently not causal (n=19, 32%).

Table 1 shows sample excerpts from the published abstracts that were evaluated. Each row corresponds to statements from the same study. The first column indicates the assigned category, based on the type of association it describes. The last column explains why a given abstract was considered to belong to the assigned category. As the assessment pertains to causal claims in general, the words referring to the particular topic of the corresponding study were removed from the statements. The examples shown are not an exhaustive list, but were chosen to illustrate the different phrasing of statements belonging to the different categories. It is worth noting that the statements presented correspond to the objective and conclusion subheadings of the abstract. When assessing the abstracts, we identified that these were the subheadings under which the information to classify the abstract was mainly found. Other subheadings like Design, Setting and Participants were not as relevant for this purpose, but were also assessed.

To further illustrate how statements in these two sections can be misleading, we tabulated a few examples in a 2 by 2 table showing mismatches between what was reported in the objectives and conclusion resulting in the paper being categorised as either 'consistently (not) causal' or 'inconsistent' (Table 2).

Assessment of published full text

Table A in supplementary material presents statements found in both the published abstract and published full text of each of these papers (n=60) regarding the statistical method used and considerations suggesting a causal aim or otherwise. Each row corresponds to a different study. The papers are grouped according to the category to which the corresponding abstract was assigned. The

For peer review only

particular causal or non-causal wording is highlighted in bold. A brief description on the consistency of causal language is provided in the last column of table, labelled 'Comment'.

We found that all papers classified as 'consistently causal' based on the abstract, also used causal language and contained causal statements in the full text. This was additionally the case with more than half (11/19) of the abstracts classified as 'consistently not causal' , where even though the abstract was carefully phrased in terms of association, the authors applied causal methods, discussed residual confounding, biological plausibility or a dose-response relationship suggesting a causal aim.

In the previous section, we referred to 3 abstracts that had a clear causal objective but a non-causal conclusion. In the full text of these papers, the authors discussed concerns of residual confounding which explains why they decided to play down the conclusion.

Looking at the methods section in the full text of the abstracts classified as 'inconsistent', we found that 11 of the 12 provided adjusted estimates. Most of the studies (8/12, 67%) used outcome regression models, mainly Cox proportional hazard models, or (propensity score) matching (3/12, 25%).

Assessment of submitted abstract version

Of the 12 published abstracts classified as 'inconsistent', we further classified 11/12 (92%) as also inconsistent on submission. There was only one study where the submitted version of the abstract described a different type of association. In this case, the conclusion of both the submitted and published versions was rather conservative by stating that the intervention was "independently associated" with the outcome. The submitted version expressed a causal objective, stating the aim of evaluating the "impact" of a particular intervention with corresponding methods: providing adjusted estimated effects and including sensitivity analysis using propensity score matching. However, in the published version the term "impact" was replaced by "association" making the abstract less clear about a causal aim because both the abstract's objectives and the conclusion described an association but the authors still provided adjusted hazard ratios and resorted to propensity score matching.

Discussion

Statement of principal findings

We found that the majority (80%) of the published research abstracts reporting on observational studies had a consistent use of causal language in the abstract. Still 20% of abstractscontained inconsistent messages on the causal nature of the key "effect". Inconsistencies showed up in two directions: an intentional quest for causality ending in uncriticized non-causal conclusions or carefully phrased mere associations ending with recommendations to act and intervene based on the exposure outcome association.

Beyond the wording, readers can learn much about the sought, after interpretation from described statistical methods, and assumptions made explicit in the paper. On a case by case basis, one could then assess whether additional assumptions, e.g. involving 'no-unmeasured confounders', would justify the causal assessment derived from these approaches. Identifying key elements like the ones presented in the Supplementary Material would help to assess if causal inference is possible. If in

For peer review only

194 doubt, a sensitivity analysis may be in order. It seems better to be transparent about the ultimate aim
195 to draw a causal conclusion and to acknowledge to fall short of that, than to generate confusion.

196 When assessing the full text of the 'consistently causal' papers, we identified that authors often
197 discussed these assumptions and resorted to conducting a sensitivity analysis. This was also the case
198 for those papers that were classified as 'inconsistent' or 'consistently no causal'. In these papers, there
199 was a concern for residual confounding because of the observational nature of the text or due to
200 specific missed confounders. Therefore the abstract's objective avoided suggesting a causal aim
201 instead of being explicit of such concern or limitations in the abstract.

202 *Comparison with other studies*

203 This is not the first study to evaluate the use of causal language in the medical literature. Cofield et al
204 (5) assessed the use of causal language in observational studies in nutrition. However, they focus only
205 on assessing whether authors included causal language or not, as it was deemed inappropriate due to
206 the observational nature of the study. We have made the case that merely avoiding explicit causal
207 terms is not a real solution. Even without them, a causal conclusion is implicit when the take home
208 message encourages interventions based on the presented findings. Avoiding inconsistency is
209 important but equally one should be able to trust that the use of consistent causal language is not in
210 vain. This requires a more in depth look at methods and assumptions validating the causal claims.

211 *Strengths and weaknesses of the study*

212 Accurate abstracts are important. In just a few brief paragraphs, the author summarizes key elements
213 of design, methods and results, and comes to a conclusion. Many readers only read the abstract.
214 However, a powerful abstract opens the door to readers and sets the scene for any study. It serves the
215 different roles of informing the audience about its main findings while motivating the reader to further
216 explore the full text, all within the constraints of brevity. This demands authors to give special attention
217 to ensure that every word in the abstract is required. All of the above makes the assessment of the
218 abstract relevant but also challenging.

219 Further research is needed to explore how causal claims presented in the abstract and full text are
220 supported by the design and methods applied, which entails assessing the methods used and
221 evaluating whether the underlying assumptions were met (19). The optimal conclusion should not
222 simply label a study as black or white in causal terms. In the present study we used a convenient limited
223 number of classifications for short statements. In practice a continuous degree of confidence in a
224 potential causal relationship is likely to emerge based on observed association.

225 We are aware that by limiting our assessment to the consistency of causal language, we may have
226 missed the discussion of the extent to which the underlying assumptions that enable causal inference
227 were met. This requires subject-matter knowledge in each particular case (13). Indeed, when there
228 was a clear causal aim but the authors considered that these assumptions were not fulfilled, they may
229 have decided that a causal claim was inappropriate and phrased their conclusion in terms of
230 association rather than causation. If this is the case, the apparent inconsistency would no longer hold.
231 On the contrary, any undue causal claims can be viewed as a form of spin (20, 21).

232 *Policy implications*

233 As observational data resources abound, methods for causal inference from observational data have
234 surged in tandem with the call for real world evidence. The new opportunities bring new challenges

For peer review only

and the responsibility for clear and well supported statements on the evidence. In this spirit and motivated by novel guidelines as proposed by ICH E9 and FDA, Miguel Hernan and collaborators have embarked on a project entitled “Developing Guidelines for the Analysis of Randomized Controlled Trials in Real-World Settings”(22). The importance of such initiatives supports a shift towards being explicit and discussing assumptions underlying causal methods that allow for causal interpretations in context, with or without an RCT (13). In the meantime, uncritical ambiguous phrasing in observational studies remains prevalent (14). Those searching for the best possible evidence supporting future treatment decisions, are best served by transparent reports of observational studies.

Faced with uncertainty when concluding on the nature of the observed exposure outcome relationship, a justifiable balance between the type I and II error rate is a natural guide for action. The cost of errors must be weighed in context, for instance as in clinical trials emphasizing control of the type I error to avoid introducing new unhelpful drugs at a potentially large cost. Alternative weights are typical in screening programs where false positives will be caught in follow-up examinations, but false negatives are lost forever. In a crisis, such as the current COVID-19 pandemic, we must act before long term randomised trials have materialised. It becomes undeniably important to learn as much as we can from observational data, be aware of the types of risk when acting or not, as displayed in Box 1.

Box 1. Impact of the errors of causal effect assignment.

		True nature of the main exposure effect	
		Causal	Not causal
Reported nature of the studied exposure effect	Causal	A true causal effect has been discovered. Recommendation to act on this should be considered. Language in the context of a study intended for causal inference.	Type I error: there is no causal effect, but it is claimed. Causal language used or suggestion to take action made when the purpose/ability was to find associations
	Not causal	Type II error: hiding the true causal objective/result by avoiding use of causal language	No causal language when the objective is prediction or to explore associations

A prerequisite for good causal language practice includes awareness of which language implies a causal statement and which does not. To support correct phrasing and raise awareness, we have compiled a short list of words and expressions with dedicated (non) causal meaning (Box 2). The list draws on phrases found in our study and in the references cited, particularly Hernan et al (10) and Thapa et al (6). This list is a suggestion as a starting point and further study can test and validate it. We consider that a definition of causal language that is generally recognized by the research community is needed (23, 24).

Words like “effect”, ”impact”, “determinant of”…, inevitably point in the causal direction and their use should come with the requirement of at least stating and ideally critically evaluating the necessary assumptions (6). Uncertainty on the causal nature of the conclusion should tone down any suggestion for intervening on the studied exposure. Specifying the corresponding level of evidence rather than

For peer review only

For peer review only - http://bmjopen.bmj.com/site/about/guidelines.xhtml

hiding the ultimate causal aim of a study is what we recommend (19), while acknowledging a margin of error in any empirical study (20).

Box 2. Examples of words and study elements that could point to causality or otherwise.

Words expressing a causal relationship	• Affect<br>• Attributable<br>• Benefit<br>• Cause/Causal pathway<br>• Contribute<br>• Determinant<br>• Effect<br>• Efficacy<br>• Impact<br>• Improve<br>• Leads to<br>• Mediates<br>• Responsible for<br>• Results in<br>• Safety
Words that could suggest causality in a given context	• Independently associated<br>• Induce<br>• Higher (lower) probability<br>• Modify<br>• Risk (factor)<br>• Trajectory<br>• (Quantitatively) decompose
Specific expressions avoiding suggestions of causal effects	• Association<br>• Correlation<br>• Less (more) likely link<br>• Predict<br>• Pattern
Key aspects suggesting causal aim	• Adjusting for confounders<br>• Discussing biological plausibility, dose-response and/or temporal relationship<br>• Discussing ‘unmeasured confounders’ assumption<br>• Mediation analysis<br>• Propensity score adjustment<br>• (Propensity score) matching<br>• Providing estimates of (population) attributable risks<br>• Suggesting/recommending intervention<br>• Target trial emulation design<br>• Using directed acyclic graphs (DAG) to identify confounders and mediators<br>• Using negative controls

For peer review only

	• Using instrumental variables

268 *Conclusion*

269 In summary, we have found that causal messages are embedded in studies otherwise carefully phrased
270 in terms of association. Further guidance for authors is needed on what constitutes a causal statement,
271 similar to the one published by Lederer et al (13) for Respiratory, Sleep and Critical Care Journals. We
272 look forward to similar guidance for other disease groups. From the screened BMJ abstracts, we
273 provided a list of expressions with clear interpretation which may inspire a useful more comprehensive
274 compendium that can be derived from a consensus meeting, for instance. We argue that such
275 awareness and special attention amongst authors and reviewers would serve our communication on
276 the best available evidence for conceived interventions.

For peer review only

Contributorship statement

- Conceptualization: COP, LB, AD, SS, EG
- Methodology: COP, EG
- Data curation: COP, LB
- Formal analysis: COP, LB, EG
- Writing original draft: COP
- Writing-review and editing: LB, SS, AD, EG
- Approved final version: COP, LB, AD, SS, EG

Competing interests declaration

SS is a full-time employee of the BMJ Publishing Group. No other competing risks to declare.

Funding statement

This project has received funding from the European Union's Horizon 2020 research and innovation programme under the Marie Sklodowska-Curie grant agreement No 676207.

Data sharing statement

The reviews and published versions of the papers included in the study are publicly available at BMJ.com. No further data will be made available as it is confidential submission data.

Ethics statement

We did not seek ethical approval or consent from authors and reviewers for this descriptive quality improvement study. However, when authors and reviewers submit manuscripts and reviews to The BMJ, they are notified that their paper or review may be entered into research projects for quality improvement purposes. COP was given access to The BMJ's data under a confidentiality agreement. We do not report any identifying information. The statements presented in this study belong to published papers available in the public domain.

References

1. Begg MD, March D. Cause and association: Missing the forest for the trees. American Journal of Public Health. 2018;108(5):620-.

2. Hernán MA. A definition of causal effect for epidemiological research. Journal of Epidemiology and Community Health. 2004;58(4):265-71.

3. Kunz R, Oxman aD. The unpredictability paradox: review of empirical comparisons of randomised and non-randomised clinical trials. Bmj. 1998;317(October):1185-90.

4. Ruich P. The use of cause-and-effect language in the JAMA Network journals. 2017.

5. Cofield SS, Corona RV, Allison DB. Use of causal language in observational studies of obesity and nutrition. Obesity Facts. 2010;3(6):353-6.

6. Thapa DK, Visentin DC, Hunt GE, Watson R, Cleary M. Being honest with causal language in writing for publication. Journal of Advanced Nursing. 2020;76(6):1285-8.

For peer review only

7. Pinsky PF, Miller A, Kramer BS, Church T, Reding D, Prorok P, et al. Evidence of a healthy volunteer effect in the prostate, lung, colorectal, and ovarian cancer screening trial. American journal of epidemiology. 2007;165:874-81.

8. Food Drug Administration (FDA), Permutt T, Scott J. E9(R1) Statistical Principles for Clinical Trials: Addendum: Estimands and Sensitivity Analysis in Clinical Trials. 2017;9.

9. European Medicine Agency (EMA). ICH E9 (R1) addendum on estimands and sensitivity analysis in clinical trials to the guideline on statistical principles for clinical trials 2020 17 February 2020

10. Hernan MA. The C-Word : Scientific Euphemisms Do Not Improve Causal Inference From Observational Data. 2018;108(5):616-9.

11. Glymour MM, Hamad R. Causal thinking as a critical tool for eliminating social inequalities in health. American Journal of Public Health. 2018;108(5):623-.

12. Green MJ. Calculating Versus Estimating Causal Effects. American Journal of Public Health. 2018;108(8):e4-e5.

13. Hernán M, Robins J. Causal Inference: What If: Boca Raton: Chapman & Hall/CRC; 2020.

14. Goetghebeur E, Cessie Sl, De Stavola B, Moodie E, Waernbaum I. Formulating causal questions and principled statistical answers. 2019.

15. Adams RC, Sumner P, Vivian-Griffiths S, Barrington A, Williams A, Boivin J, et al. How readers understand causal and correlational expressions used in news headlines. Journal of Experimental Psychology: Applied. 2017;23(1):1-14.

16. Haber N, Smith ER, Moscoe E, Andrews K, Audy R, Bell W, et al. Causal language and strength of inference in academic and media articles shared in social media (CLAIMS): A systematic review. PLoS ONE. 2018;13(5):1-21.

17. Lederer DJ, Bell SC, Branson RD, Chalmers JD, Marshall R, Maslove DM, et al. Control of Confounding and Reporting of Results in Causal Inference Studies. Guidance for Authors from Editors of Respiratory, Sleep, and Critical Care Journals. Annals of the American Thoracic Society. 2019;16(1):22-8.

18. Harhay MO, Au DH, Dell SD, Gould MK, Redline S, Ryerson CJ, et al. Methodologic Guidance and Expectations for the Development and Reporting of Prediction Models and Causal Inference Studies. Annals of the American Thoracic Society. 2020;17(6):679-82.

19. Hernán MA, Robins JM. Estimating causal effects from epidemiological data. Journal of Epidemiology and Community Health. 2006;60(7):578-86.

20. Boutron I. Reporting and Interpretation of Randomized Controlled Trials With Statistically Nonsignificant Results for Primary Outcomes. JAMA. 2010;303(20):2058-.

21. Ghannad M, Olsen M, Boutron I, Bossuyt PM. A systematic review finds that spin or interpretation bias is abundant in evaluations of ovarian cancer biomarkers. Journal of Clinical Epidemiology. 2019;116:9-17.

For peer review only

22. Developing Guidelines for the Analysis of Randomized Controlled Trials in Real-World Settings [Available from: https://www.pcori.org/research-results/2015/developing-guidelines-analysis-randomized-controlled-trials-real-world.

23. Doan S, Yang EW, Tilak SS, Li PW, Zisook DS, Torii M. Extracting health-related causality from twitter messages using natural language processing. BMC Medical Informatics and Decision Making. 2019;19(Suppl 3).

24. Chiolero A. Causality in public health: One word is not enough. American Journal of Public Health. 2019;109(10):1319-20.

For peer review only

Tables

Table 1. Examples of statements found in the objectives and conclusions sections of abstracts of observational studies published in The BMJ in 2018 and their corresponding assigned category.

Assigned category	Abstract Objectives	Abstract Conclusions	Comment
Consistently causal	"...assess the effectiveness of..."	"Little evidence was found of a direct impact of..."	When discussing associations, words like effect, contribution or role are similar to cause and then (direct) impact and effect will be their consequence
	"To determine the effect of ... in ..."	"...has led to risk..."	
	"To describe the contributions of..."	"... an important role in ..."	
	"To evaluate the impact of ..."	"...impacts are..."	
	"To investigate whether improving adherence to ...."	"...the beneficial effect of improved..."	Evaluates taking an action 'improving adherence' and concludes that the effect is beneficial
	"...benefit of ... in reducing ... risk"	"... is an overlooked risk factor for ..."	Evaluates how a given intervention can reduce the risk of an outcome and then labels it as an 'overlooked risk factor'
	"To determine outcomes and safety of..."	"... is at least as effective and safe as ..."	Evaluates and determines that a certain intervention is as safe as the comparator

For peer review only

	"to quantitatively decompose this joint association to ... only, to ... only, and to their interaction."	"...excess risk of...These findings suggest that most cases of ... could be prevented by ..."	Suggests interest in direct and indirect effect i.e. mediation analysis, and concludes consequently
Consistently not causal (Associations)	"...is associated with ...compared with..."	"...is associated with ...compared with..."	Describes associations without labelling them as causal or prediction
	"To describe trends in..."	"...rates were high during the study period of ... with the highest rates in ... versus ..."	Limits to describe frequency
	"To assess how often ..."	"One in ... adults ... were ..."	
	"To examine the association between..."	"...could increase ... confirmation of these findings are warranted, preferably in an intervention setting."	Suggests further research to determine the nature of the association
	"...compared with...is associated with..."	"Additional studies, with long term follow-up, are needed to investigate the effects of..."	
Consistently not causal (Prediction)	"To develop and validate a set of practical prediction tools that reliably estimate the outcome of..."	"...prediction models reliably estimate the outcome..."	Describes developing and validating prediction models

For peer review only

	"To prospectively validate the … algorithm to …"	"…accurately classified…"	
Inconsistent	"…evaluate safety of…"	"…associated with…"	Phrasing the objective as causal and limiting to describing an association in the conclusion
	"…analyse the effect of…"		
	"…critical determinant…"		
	"…association with…"	"…is safe…"	Phrasing the objective as just to explore an association and presenting a causal claim in the conclusion
		"… had no substantial effect on long term survival…"	
		"… was determined by… may be largely explained by…"	
		"… was found to be the safest drug, with reduced risks of…"	
		"These results emphasise the benefit of…"	
	"…association with…"	"…tackling all these risk factors might substantially… "	Phrasing the objective and conclusion as if just to assess an association but then suggesting to take

For peer review only

		"...Targeting ... prevention strategies among these patients should be considered."	action given the findings
		"Systematically addressing ... may be an important public health strategy to reduce the incidence of"	
		"...present findings encourage the downward revision of such guidelines ..."	

For peer review only

Table 2. Examples of (mis)matching causal and non-casual statements found in the objectives and conclusions sections of abstracts of observational studies published in The BMJ in 2018.

		Abstract Conclusions	
		Causal	Not causal
Abstract Objectives	Causal	Consistent<br>"...assess the effectiveness of..."and "Little evidence was found of a direct impact of..."<br>"...benefit of ... in reducing ... risk" and "... is an overlooked risk factor for ..."	Inconsistent<br>"...evaluate safety of..." and "...associated with..."<br>"...analyse the effect of..." and "...associated with..."<br>"...critical determinant..." and "...associated with..."
	Not causal	Inconsistent<br>"...association with..." and "...is safe..."<br>"...association with..." and "... had no substantial effect on long term survival..."<br>"...association with..." and "...tackling all these risk factors might substantially... "<br>"...association with..." and "Systematically addressing ... may be an important public health strategy to reduce the incidence of"	Consistent<br>"To describe trends in..." and "...rates were high during the study period of ... with the highest rates in ... versus ..."<br>"To assess how often ..." and "One in ... adults ... were ..."<br>"To develop and validate a set of practical prediction tools that reliably estimate the outcome of..." and "...prediction models reliably estimate the outcome..."

For peer review only

Supplementary Material

Table A. Statements in each of the included observational studies published in the BMJ in 2018

Published Abstract		Published Full text				Comment
Objective	Conclusion	Method	Confounder adjustment	Estimates provided	Authors Considerations	
CONSISTENTLY CAUSAL						
"To evaluate the **impact** of […] on […] in […]"	"[…] is associated with negative **effects** on […]. Given the relatively low prevalence of […], population level impacts are currently modest. Nevertheless, as […] has	"To estimate the **adjusted** odds ratio for each […] outcome by […] group, we created logistic regression models with […] as the reference group. […] The **population attributable risk** was calculated using the standard formula"	"Other subgroup analyses were done to ensure that […] association was **not confounded by** […]"	"[…] had 14% higher odds of […] compared with […] (adjusted odds ratio 1.14, 99% confidence interval 1.13 to 1.15). […] 14.5% (13.6% to 15.4%) of […] (under the assumption of a **causal** relation)	"The pooling of all […] during this period minimizes the risk of **confounding** from yearly fluctuations in […] outcomes. Finally, despite attempts to adjust […] using regression analysis and	Both abstract and main text use causal language: they state that their aim is to assess the impact of the exposure on the outcome, adjust for confounders, provide

For peer review only

	doubled in the US over the past generation, further investigation is warranted of the **impact** on […] and public health.			can be **attributed to** […]"	stratification, some **residual confounding** effects from […] could remain. […] As more than 12% of […] **might have been prevented** were […], the importance of these data are most relevant to […]. The cumulative risk over […] is also likely to be **important in terms of both economic burden and overall public health**"	population attributable risks and discuss residual confounding.
"To examine the […] **risks of […] initiation** compared with	"[…] **poses a […] risk** compared with non-use, […] use, and use	"[W]e conducted a series of cohort studies, each mimicking the strict design criteria of a clinical trial (a	"We calculated the **propensity score** for all eligible	"[…] initiators had a 50% **increased rate of** […] events compared with	"We performed the following sensitivity analyses, […] to	The use of causal language appears in

For peer review only

initiation of other traditional […] drugs, initiation of […], and no initiation."	of other traditional […] drugs."	so-called **emulated trial design**), to compare rates of […] among […] with rates among […]. […] We estimated an observational analogue of the **intention to treat** hazard ratio, as a measure of the incidence rate ratio, by fitting a Cox proportional hazards model, using time since start of follow-up as the time scale and a time independent covariate for treatment assignment. We pooled data from all trials into one model and included each trial as a stratum in the regression (using values from 1 to 252)."	individuals initiating […] at enrolment by fitting a logistic regression model including covariates on sex, age, year, comorbidity, and drug treatment use. We then **matched** non-initiators to […] initiators (1:1) by **propensity score** within a maximum **matching** range of 0.025 and without replacement."	[…] non-initiators (incidence rate ratio 1.5, 95% confidence interval 1.4 to 1.7)."	estimate how strongly a single **unmeasured** binary **confounder** would need to be associated with […] to fully explain our findings. […] Finally, an **unmeasured confounder** that was twice as frequent among […] initiators versus among non-initiators would still need to increase the risk of […] by a factor of nine or more to fully explain the results, if no increased risk actually existed (eFigure 3). […] Still, the	both abstract and main text. They apply causal methods including target trial emulation and propensity score matching and discuss residual confounding.

For peer review only

					**emulated trial design** lacked baseline randomisation, and therefore, **unmeasured confounding** cannot be excluded.”	
“To explore **associations** between […] **and later**[…], **overall and by** […] **subtype** and timing of onset.”	“[…] was associated with an **increased risk** of […], particularly [subtype]. […] were unlikely to **mediate** the associations substantially, suggesting that […] and […] may share underlying mechanisms or susceptibility pathways.	“We used Cox regression with age as the underlying time to estimate hazard ratios for […] comparing women with and without a history of […] We used competing risk methods when analysing associations with […] subtypes. […] We evaluated potential **mediation** by […]”	“We considered […] as a priori **confounders**.”	“Women with a history of […] had a 53% **increase in risk of** […] overall, compared with women with no history of […] (incidence rate for women with a history of […]: 11.6 per 100 000 person years; incidence rate for women with no history of […]: 8.33 per 100 000 person years;	“we did sensitivity analyses […] using the array approach for testing the effect of an **unmeasured or incompletely measured confounder.** […] Sensitivity analyses suggested that **confounding** by […] was **unlikely to explain** the	Causal language is present in abstract and main text. They apply mediation analysis, adjust for confounders, discuss the unmeasured confounding assumption and residual confounding.

For peer review only

	Asking about a history of […] could help physicians to **identify women who might benefit from** screening for early signs of disease, allowing for early clinical intervention.”			hazard ratio 1.53, 95% confidence interval 1.26 to 1.85).”	observed associations for […]; in contrast, […] **could** conceivably **explain** a considerable part of the association between […] and […]. […] we also cannot rule out the possibility of **residual confounding** by other unmeasured covariates”	
“To investigate whether adults with […] **are at an increased risk of** […] and **whether the risk varies by** […] severity and condition activity over time.”	“Severe and predominantly active […] are associated with an increased risk of […] outcomes. **Targeting […] prevention**	“We used Cox regression stratified by **matched** set […] with current age as the underlying timescale to generate hazard ratios for the association between [..] and each […] outcome (the unadjusted model). Subsequent multivariable	“For each patient with […], we **randomly matched** up to five patients by age (within 15 years), sex, general practice, and calendar	“Table 3 shows that in the primary analysis, there was evidence of **associations** between […] and all […] outcomes, except for […].	“Limitations of the study, inherent to most large observational studies, include the possibility for **confounding**, bias, and missing	The causal aim is evident in the abstract because they intend to assess how the risk of the outcome varies when

For peer review only

	**strategies** among these patients should be considered."	analyses adjusted for [...](the **adjusted** model). The adjusted model was further adjusted for variables which may have been on the **causal** pathway (ie, **mediators**) between [...] and [...] outcomes [...] (the **mediation** model). [...] The **population attributable risk** of each [...] outcome was estimated by using the estimated hazard ratio and assuming the prevalence of [...] to be 10%."	time at cohort entry. These unexposed patients were required to have at least one year of follow-up in CPRD and no history of [...] when matched. [...] We used a **directed acyclic graph** to inform the identification of covariates and **mediators** and to avoid **collider bias**"	**Associations were strongest with** [...] (hazard ratio 1.25, 99% confidence interval 1.11 to 1.41 in the adjusted model) and [...] (1.19, 1.10 to 1.30), with **partial attenuation in the mediation model**. [...] The greatest **population attributable risks** were estimated for [...] (2.4%, 1.1% to 3.9%) and [...] (1.9%, 1.0% to 2.9%)."	data. [...] We **have shown a clinically relevant increase in the risk of** [...] outcomes in patients with [...].This increased risk is largely confined to patients with severe or more active [...] and persists despite adjusting for potential mediators, including conventional risk factors for [...] outcomes. Consideration should be given to **developing prevention strategies to reduce the risk of** [...] among patients with	the exposure is modified and the conclusion is to take action given the findings. The main text uses causal language, discusses DAGs, mediators, collider bias and provides population attributable risks.

For peer review only

					severe or predominantly […]”	
“To **determine the effect of** […] outcome reporting in […] on risk averse clinical practice, “gaming” of clinical data, and 90 day postoperative mortality.”	“This study **did not find evidence that** the introduction of […] in […] **has led to risk** averse clinical practice behaviour or “gaming” of data. However, **its introduction coincided with** a significant reduction in 90 day mortality.”	“We used a **change point analysis** to study the change over time in **adjusted** 90 day mortality after […] and after […]. We used a multivariable logistic regression model for 90 day mortality, with a slope for calendar time and an interaction between time pre-introduction versus post-introduction of […], in addition to all of the **risk adjustment variables**. This modelled a change in the slope of mortality at the point that […] was introduced but no immediate change in mortality.”	“The **risk factors** included in this logistic regression model are […]. An adjusted outcome was then produced by indirect **standardisation**”	“The 90 day mortality in patients undergoing an […] fell during the study period from 952/33 638 (2.8%) before the introduction of […] to 552/25 905 (2.1%) after (fig 4). Therefore, we carried out change point analysis which showed a steeper decline in 90 day mortality after the introduction of […] (P=0.03). The **change point analysis also found a**	“If we assume that the decrease in mortality can be **causally linked** to […], the process of […] This team response could have been **mediated** through […]”	Causal language is present in abstract and main text as they state that their aim is to determine the effect of the exposure on the outcome and conclude that the exposure has not led to the outcome. The main text explainis that confounder adjustment was made through standardisatio

				**significant effect of** […] when it was modelled as an immediate shift in 90 day mortality (P=0.01) and when it was modelled as both an immediate shift and a change in slope (P=0.04)."		n and discuss possible mediators for this relationship.
"To assess the **effectiveness of** the […] policy initiatives **in improving** […] **and reducing** […] in survival in England."	"Little evidence was found of a **direct impact** of […] on one year survival, and no evidence for a reduction in […] in cancer survival. These findings emphasise that […] in survival remain a major	"We estimated one year **net survival** for each […] by sex, year of diagnosis (1996 to 2013), and deprivation category. Patients with a diagnosis between 1996 and 2013 had the potential to be followed up for at least one year, so we used the classic cohort approach. […] We estimated net survival using the	"Survival estimates for all ages combined were age **standardised** with the International Cancer Survival Standard weights. […] We used multivariable linear regression	"One year survival **improved** for 20 of the 21 […] examined in women and 16 of the 20 […] examined in men. […] For these […], the average annual absolute increase in one year age	"The lack of **consistent** results between men and women, as well as the lack of general patterns across […] types, provide **little evidence for any strong impact** of the […] policies on short term […] survival. The	Causal language is present in both abstract and main text. The aim is to evaluate the effectiveness of a policy on a given outcome, they provide standardised

	public health problem for a healthcare system founded on equity.”	consistent nonparametric estimator defined by Pohar-Perme.”	to investigate the survival patterns for each […] and by sex”	**standardised net survival** was often greater than 1% over the whole study period”	evidence is even weaker for their **impact** on the […] in […] survival. […] **These findings should be taken into consideration by** […] policy makers and inform future initiatives.”	net survival and suggest to take action given the findings.
To investigate whether **improving** adherence to […] **interacts with** the genetic predisposition to […] in relation to long term changes in […] and […].”	“These data indicate that improving adherence to […] could **attenuate** the genetic association with […]. Moreover, the **beneficial effect** of improved […] on […] was particularly	“We used multivariable generalized linear models with repeated measures analyses to assess the main **associations of** the […] **and changes in** the […] **with change in** […]”	“We used multivariable models to **adjust for** […]”	“In general, the […] was **associated with increases in** […] every four years: in the two cohorts combined, each additional […] was associated with 0.02 (SE 0.01) increase in […] and 0.05 (SE 0.03) kg increase in […]”	“[…] **unmeasured or unknown confounders** may also exist. Secondly, because adherence to […] was not randomized, the association between […] and […] may not imply a **causal relation.** Thirdly, the results could be	Assessing if improving adherence has an effect translates to an intervention that is being assessed. They conclude that there is a beneficial effect and suggest to take action. The

For peer review only

	pronounced in people at high genetic risk for […].				underestimated by potential **reverse causality**. […] Our study provides reproducible evidence from two prospective cohorts of US men and women that **improving […] could attenuate** the […] association with […]"	main text discusses unmeasured confounding assumption and reverse causality. All of the above is consistent with a causal aim.
"To assess the **independent** and joint **associations** of [….] and […] with […] risk and to explore the **benefit of […] in reducing** the […]	"[…] is an overlooked **risk factor** for […], as important as five major lifestyle factors combined. In this study, […] **contributed** to more than one	"We calculated the time to event from the date of enrollment to the date of […] incident or […] death, death due to causes other than […], or the end of cohort follow-up (31 December 2008), whichever came first. We used Cox proportional	"[…] **adjusting for** […]."	"A statistically significantly increased risk of incident […] was observed for the eight diseases and markers. Specifically, […] was inversely associated with	"More evidence was needed to clarify whether the inverse association was **causal** or related to […]. […] the **dose-response relation**, the exclusion of […]	Causal language is present in abstract and main text. They identify that the exposure contributes to the outcome,

For peer review only

risk associated with […] and […].	fifth of the risk for incident […] and more than one third of the risk for […] death. […] is associated with a nearly 40% **reduction in the […] risk** associated with […].”	hazards model to estimate hazard ratios and 95% confidence intervals”		risk of incident […] in a **dose-response manner**”	during recent follow-up, and further adjustment for […] minimize the likelihood of **reverse causation** and lend support for **causality**. […] Our study uncovered a **substantial impact** of […] jointly on […] risk, which were equally as important as five lifestyle factors combined.”	after adjusting for covariates. They discuss dose-response relationship and reverse causality .
“To examine the **association** between […] and […] **in later life**, and **determine** whether the maintenance of	“These results show that […] is **not associated with the trajectory of** […] in late life, but is	“The raw scores from the […] tests were **standardised to** a mean of 100 and a standard deviation of 15 to produce an […] scale. Age at testing was the number of years	“Because our sample were all born in the same year and tested at a similar age, a **confounder** for age at entry was	“ The typical intellectual engagement models for each domain are shown in table 2 and indicated **an**	“In our statistical models, we introduced **possible confounders** available from early life and life	The causal aim is suggested when the goal is to establish temporal relationship between

For peer review only

[...] will offset age related [...]”	associated with the acquisition of ability during the life course. Overall, findings suggest that high performing adults engage and **those that engage more being protected from** relative decline.”	after participants’ 60th birthdays. We modelled age in this form so that the intercept occurred at age 60 years rather than zero years, such that the calculation for the intercept would represent a realistic adult value rather than one extrapolated 64 years earlier. [...] For each model, a probability value of P<0.05 was considered significant.”	not used. We modelled cognitive performance with a linear mixed model, as a combination of [...].”	**expected significant decline** in [...] with age, ranging from −1.09 to −1.31 standard points per year for the [...] test and −0.77 to −1.69 for the [...] test. [...] None of the age×TIE interaction terms were significant, indicating that [...] **did not influence the trajectories of** age decline.”	course, including [...]. We also **controlled for** [...] associated with repeated testing. [...] significant **associations remained after adjustment for** age, sex, and test practice effects. [...] is an **independent contributor to** late life [...] and has a unique effect over and above the effect of other life course variables. [...] It is, however, impossible for a **causal effect** to be inferred [...]”	exposure and outcome, describing the exposure as a trajectory, providing standardised and adjusted estimates and considering whether it is possible to infer a causal link.
“To evaluate the **associations** of a	“ In this cohort study, [...] were	“To test the **association** of [...] and [...] with [...] we	“Cox proportional hazards models	“In Cox proportional	“The present study provides	The abstract describes that

[…] and […] with **incident** […]."	**independently associated with incident** […]. These results **emphasise the benefit of** entire populations adhering to […], independent of […] risk."	used Cox proportional hazards models. The duration of follow-up was calculated as time between the baseline assessment and the first event of either […] or 1 March 2016, which was the end of follow-up for the current data release. Participants who had a […] before a […] occurred were censored at the time of the respective event."	included **adjustment for** age and sex for the lifestyle score models. For the models including the genetic score we additionally **adjusted for** the first 10 principal components of ancestry and genotyping batch."	hazards analysis, the **risk of** […] was higher for those with […] (hazard ratio 1.20, 95% confidence interval 1.08 to 1.34) and […] (1.35, 1.21 to 1.50) **compared with** those with a low genetic risk score"	further support that common […] are **implicated in the development of** […]. […] The […] was also associated with […], which suggest that the **effect of** the […] on risk of incident […] might at least in part be **mediated by** […]. The effects of […] might differ according to the cause of […], although some […] factors are shared between two or more **causal** factors"	the goal is to establish a temporal relationship between the exposure and the outcome and suggest to take action given the findings. The main text describes adjusting for covariates and discusses mediation.
"To determine the **longitudinal association**	"In older adults, a **higher cumulative**	"We used a Cox proportional hazards model to evaluate the	"We selected covariates and potential	"Figure 1 shows that **after multivariable**	"[…] we excluded participants […] who reported	The abstract describes that the goal is to

between […] and […].”	**level of** […] was associated with a **higher likelihood** of […]. These findings **support guidelines for** increased dietary consumption of […] in older adults.”	**association between time-varying** […], **adjusting for time-varying covariates** (updated at […] measurement), and the likelihood […]. Time at risk was from the first […] measurement until the first […] event or censoring […] or the latest date of adjudicated follow-up in June 2015.”	**mediators** based on biological interest, current or previously observed associations with […], and meaningful changes in the exposure risk estimate (±5%). Minimal **adjustments included** age and sex. **Multivariable adjustments** additionally included […]. We used the potential mediators to explore what additional associations could exist to these potential **pathways**.”	**adjustment for** demographic, lifestyle, cardiovascular risks, dietary habits, and other […], higher […] levels were associated with a lower likelihood of unhealthy ageing. Overall, participants in the highest group of […] had an 18% (95% confidence interval 3% to 30%; P=0.001) lower risk of […].Findings were not appreciably altered after adjustment for potential **mediators** (not shown).”	baseline […] (to avoid **reverse causality**; n=195). […] The community based design improves **generalizability**, and regular physical examinations ensured that demographics and other risk factors were well measured, which may help to minimize **confounding**. […]The possibility of **residual confounding** by imprecisely measured or unknown factors also cannot be excluded for an observational study. […] *Any unmeasured*	establish a temporal relationship between the exposure and the outcome and suggest to take action given the findings. The main text describes adjusting for covariates and discusses mediation, residual confounding and reverse causality. The statement in italic is a clear causal statement.

					confounders would have to be strongly associated with both the exposure and the outcome, conditional on all the variables already in the model. Thus, it seems unlikely that either poorly measured or unmeasured confounders could fully account for our findings."	
"To **prospectively** evaluate the joint **association** of […] and […] with risk of type 2 diabetes risk, and to **quantitatively decompose** this	"Among female nurses, both […] were associated with a higher risk of […]. **The excess risk of** […] was higher than the	"Participants contributed person time from the return of the baseline questionnaire [..] until the date of diagnosis of […], death, loss to follow-up, or the end of the follow-up period (30 June 2012 for	"Information on **potential confounders** was assessed and updated every other year via the questionnaires throughout	"We observed a positive association between duration of […] and risk of […] in both cohorts. Compared with	"From a public health standpoint, because 71% of the joint effect could be attributed to an […], our findings	Their aim is to establish a temporal relationship exposure-outcome, they discuss adjusting for

For peer review only

joint association to […]"	addition of risk associated with each individual factor. These findings suggest that most cases of […] **could be prevented by** […], and the **benefits** could be greater in […]."	the NHS and 30 June 2013 for NHS II), whichever came first. We used **multivariable time dependent** Cox proportional hazards models to estimate hazard ratios and 95% confidence intervals for the associations between […] alone and in combination with […]."	follow-up. This information included […] In multivariable analysis, we **adjusted for** several **confounding** factors including […] We also examined the **decomposition of the joint effect**: the proportion **attributable** to […]"	women without rotating night shift work, the pooled **multivariable adjusted** hazard ratios for women with 1-5, 5-9, and 10 or more years of […] were 1.11 (95% confidence interval 1.00 to 1.22), 1.28 (1.10 to 1.49), and 1.46 (1.33 to 1.62) (P for trend <0.001) […](table 3). […] The **attributable** proportions of the joint **effect** were 17.1% (95% confidence interval 14.0% to 20.8%) for […] alone, 71.2% (66.9% to 75.8%) for […]alone, and 11.3% (7.3% to	underscore the importance of maintaining […].Our findings suggest that most cases of […] could be **prevented by** […], and the benefits would be larger in […] "	confounders, provide excess risk and suggest to take action given the findings to prevent the outcome.

For peer review only

				17.3%) for their interaction.”		
“To assess the **association** between […] and seven serious **adverse events** of current concern.”	“In this analysis of nationwide registers from two countries, use of […], as compared with […], was associated with an **increased risk of** […], but not with other serious **adverse events** of current concern.”	“Patients were followed from cohort entry to treatment cessation, crossover to the other study drug […], the outcome event, death, emigration, or the end of the study period (31 December 2016). We used Cox proportional hazards regression to calculate hazard ratios, analysing each outcome independently. The **absolute risk difference** was calculated as hazard ratio−1 multiplied by the rate in the comparator group.”	“We used an active comparator new-user study design and controlled for a wide range of potential **confounders** (patient characteristics that might be associated with both the outcome and the decision to initiate a drug) through a non-parsimonious **propensity score model** to minimise the risk of bias, including **confounding by indication** […].	“Use of […], as compared with […], was associated with an increased risk of […] (hazard ratio 2.32, 95% confidence interval 1.37 to 3.91) and […] (2.14, 1.01 to 4.52) but not with […] (1.11, 0.93 to 1.33), […] (0.69, 0.45 to 1.05), […] (0.89, 0.67 to 1.19), […] (0.99, 0.71to 1.38), or […] (1.16, 0.64 to 2.12).”	“The findings should be interpreted in the context of limitations of observational studies and the uncertainty of the **effect estimates**. […] Therefore, the studies could suffer from compromised **confounding** control, as indicated by the imbalance in […] at baseline between users of […] versus comparators, even after propensity score matching […]	The goal is to evaluate the association with the exposure to adverse events which translates into assessing safety. They discuss adjusting for confounding, resort to propensity score matching and consider residual confounding.

			We estimated **propensity scores** by using logistic regression for the probability of […] conditional on the status of 66 covariates, defined and selected a priori, including sociodemographic characteristics, comorbidities, comedications, and healthcare utilisation […] We matched [..] and [..]t users (1:1 ratio, by country) according to propensity score, by using the nearest neighbour matching algorithm (caliper width 0.2 of the		Finally, **residual and unmeasured confounding** affecting the findings in our study cannot be ruled out.”	

For peer review only

			standard deviation of the logit score). Analyses were performed in a pooled dataset of the two countries."			
"To determine whether patients [...] have **fewer** [...] and **higher rates** of [...] than patients [...].	***In results*: "Per 100 000 patients, there were 2999 **fewer** follow-up appointments within 14 days, 26 **excess** deaths, [...] **attributable to** [...]."<br><br>"Patients [...] are **less likely to** have [...] and are at higher risk of [...]."	"For all outcome comparisons we report unadjusted and **adjusted** odds ratios (with 95% confidence intervals). **Adjusted** odds ratios were obtained with logistic regression models estimated using generalised estimating equations methods and including all measured patient and hospital characteristics."	"We examined hospital type and several characteristics of patients and admissions: year of Charlson comorbidity index score, socioeconomic status (measured using median neighbourhood income), length of hospital stay, arrival by ambulance, diagnosis,	"Patients [...] were **less likely** to have follow-up with a physician within seven days (36.3% v 47.8%, adjusted odds ratio 0.61, 95% confidence interval 0.60 to 0.62) and 14 days (59.5% v 68.7%, 0.65, 0.64 to 0.66). [...] Patients discharged during the holiday period were at	"A confirmatory time-to-event analysis in a **propensity score matched cohort** (see supplementary appendix table 5) showed consistent results (death or readmission hazard ratio 1.08, 95% confidence interval 1.07 to 1.09). [...] The differences in outcomes **could**	The abstract and main text use causal language: they discuss risk attributed to the exposure, provide adjusted estimates using propensity score matching and discuss residual confounding.

For peer review only

			discharged with home support or against medical advice, and previous healthcare usage (emergency department visits, hospital stays, outpatient visits, home care visits). hospital discharge, age, sex, rural residence, ”	**increased risk of** death or readmission within 30 days (25.9% v 24.7%, 1.09, 1.07 to 1.10). This was **explained by** an increased risk of return to the emergency department (24.3% v 23.0%, 1.09, 1.07 to 1.10), rehospitalisation (11.8% v 11.4%, 1.06, 1.04 to 1.08), and death (1.5% v 1.5%, 1.06, 1.02 to 1.10) within 30 days”	**not be explained by** observed hospital or patient characteristics, including admission diagnosis. […] the possibility of **confounding** due to unmeasured differences remains”	
“To investigate the **association** between […] and	“Use of […] is **associated** with a reduction in	“We calculated age **standardised** incidence rates of […] per 100 000	“**Adjusted** incidence rate ratios (referred to	“The age **adjusted** incidence of […]	“The data linkage study design also enabled us to	When assessing the exposure-

For peer review only

overall and specific types of […].”	[…] risk in women of reproductive age—an **effect related to duration of use**, which diminishes after stopping use. These data suggest no **protective effect** from […].”	person years, using the age distribution of the cohort as standard. Risk of […] among users of the different product groups was analysed by a Poisson regression model in SAS version 9.3 (SAS Institute). […] We calculated the population prevented fraction (population prevented fraction = prevalence$_{exposure}$(1−relative risk)) associated with ever use of […] by using the relative risk of never use versus ever use of […]. The population prevented fraction is the proportion (expressed as a percentage) of the […] in the cohort that has been prevented by ever use of […].”	here as relative risks) and their surrounding 95% confidence intervals were calculated for each model, with never users as the reference group. The **adjusted** models included the following time varying covariates […]”	was highest in women who were never users of […] (7.5 per 100 000 person years; table 2). Among ever users of […], **reduction** in the age standardised absolute rate of […] was 3.2 per 100 000 person years. Overall, ever users of […] had a reduced risk of […] compared with never users (relative risk 0.66 (95% confidence interval 0.58 to 0.76)). […] **use of […] prevented** 21% of […] in the study population”	**adjust** for several important **confounding** variables. We were not able to adjust for some factors, such as […] Our findings, therefore, could be subject to **residual confounding**.”	outcome relationship, they provide standardised estimates, discuss residual confounding, dose-response and provide attributable fraction.

For peer review only

"To assess whether [...] is **associated with a reduction** in [...] and mortality in old and very old adults with and without [...]."	"In participants older than 74 years without [...], [...] was **not associated with a reduction in** [...] or in all cause mortality, even when the incidence of [...] was **statistically significantly higher** than the risk thresholds proposed for [...]. In the presence of [...], [...] was statistically significantly associated with reductions in the incidence of [...] and in all cause mortality. This **effect** decreased after age 85 years	"To prevent survivor bias and covariate measurement bias, we selected a "new users design" over "all [...] users. [...] Using Cox proportional hazard regression models **adjusted by propensity score**, we calculated the hazard ratios of statin use for the outcome events. Participants were censored at the date of transfer from SIDIAPQ or at the end of the study period."	"We explored the variables associated with [...] to determine candidate variables for the propensity score of [...]. From SIDIAPQ we obtained data on age, sex, [...]Because of non-random treatment allocation, we used a logistic model based on **potential confounding** covariates to calculate the **propensity score** of [...]. We calculated the propensity score separately for participants with and without [...]	"In participants without [...], the hazard ratios for [...] were 0.94 (95% confidence interval 0.86 to 1.04) for [...] and 0.98 (0.91 to 1.05) for all cause mortality in 75-84 year olds. [...] The one year **number needed to treat** was 164 for [...] and 306 for all cause mortality."	"To prevent **residual confounding** we performed additional regression adjustments after adjustment of **propensity score**. Variables that remained imbalanced after propensity score adjustment were also included in the models. [...] Despite these efforts, we acknowledge that some **residual confounding** might exist."	They use causal language and causal methods. To estimate the effect of the exposure on the outcome. they use propensity score methods to adjust for confounding, provided a NNT and consider residual confounding.

	and disappeared in nonagenarians. ”		and also within each age group, and standardised differences before and after adjustment for **propensity score**. Variables with standardised differences <0.10 were considered to be **well balanced**.”			
“To examine the **association** between [...] and risk of [...].”	“The risk of [...] was increased in people who [...]. In several countries, guidelines define thresholds for [...]. The present findings **encourage the downward**	“Cox regression was used in all analyses, with age as the timescale to model the associations with hazard of incident [...]. Participants were censored at date of record of [...], death, or 31 March 2017, whichever came first.”	“Models were first **adjusted** for sociodemographic factors, then additionally for health behaviours, and finally for health status.”	“[...] was associated with a **higher risk** of [...] when the reference was [...]; in these analyses [...] was associated with an increased risk of [...] in a linear fashion (among those [...], P for non-	“[...] multistate models showed that part of the excess risk of [...] in [...] was **attributable to** the greater risk of [...] in this group. [...] We accounted for several sociodemographic and health	The abstract and main text use causal language, they provide adjusted, discuss residual confounding and suggest to take action given the findings.

For peer review only

	**revision of such guidelines** to promote […] at older ages.”			linearity=0.97 using spline regressions). In a model **adjusted for** sociodemographic factors […] was associated with a **greater risk** of […] (hazard ratio 1.47, 1.15 to 1.89) compared with […]”	related characteristics in the analysis, but **residual confounding** cannot be excluded as an explanation for the higher risk of […] among […]. Our multistate models lent partial support for a **mediating** role of […] in the association between […] and increased […]”	
“To assess whether **adding or switching to** […] is associated with an increased risk of […], **compared with remaining** on […]	“[…] as second line drugs are associated with an increased risk of […] compared with remaining on […]. Continuing	“The study cohort was formed by identifying all subjects from the base cohort of […] initiators who subsequently added or switched to a […] as second line treatment. Patients who added or switched to	“[…] exposed and reference subjects were **matched on high-dimensional propensity score**. The high-dimensional	“Compared with the use of […], adding or switching to […] was associated with an increased risk of […] (7.8 v 6.2 per 1000	“Based on a post-hoc analysis, the findings of the primary analysis on […] unlikely to be the result of an **unmeasured confounder**	The aim is to assess the effect of the intervention strategy of adding or switching to a particular

For peer review only

in patients with […].”	[…] when introducing […] appears to be **safer** than switching.”	other […] were censored. For each patient adding or switching to a […], we identified a matched reference patient who also was a […] initiator but remained on metformin, using a prevalent new-user design. […] we constructed a Cox proportional hazards regression model for each outcome that estimated the hazard ratio and the 95% confidence intervals for […] versus […].”	**propensity score method** empirically selects covariates based on their prevalence and potential for **confounding**. For each member of each matched set, we identified all available information from seven data dimensions (five dimensions from the CPRD: drug prescriptions, procedures, diagnoses, disease history, and administrative information; two dimensions form the HES: diagnoses and procedures) in the one year	person years, hazard ratio 1.26, 95% confidence interval 1.01 to 1.56), all cause mortality (27.3 v 21.5, 1.28, 1.15 to 1.44), and […] (5.5 v 0.7, 7.60, 4.64 to 12.44). There was also a trend towards increased risks of […] (6.7 v 5.5, 1.24, 0.99 to 1.56) and […] (9.4 v 8.1, 1.18, 0.98 to 1.43).”	under most plausible exposure-confounder and confounder-outcome associations. […] For our study, we used the recently developed prevalent new-user design. To **emulate** the randomised controlled trial, this design identifies (at the doctor visit that led to the patient on […] adding or being switched to […]) a comparable patient with the same history of […] use and of other characteristics, but who on that	drug, they emulate a target trial, use propensity score matching. discuss residual confounding and conclude that the strategy is safe.

For peer review only

			period before the date of the **matched** set. We then applied conditional logistic regression to estimate the **propensity** of receiving a […] drug, thereby considering the 500 most likely **confounders**."		visit continued on […]. […]owing to its observational nature there is the potential for **residual confounding**."	
"To investigate the **associations** between […] and **risks of** […] compared […]."	"Overall, […] was found to be the **safest** drug, with reduced risks […] compared with […]. […] and low dose […] were, however, associated with increased risks of all cause mortality	"We used a new-user design to capture all events occurring after starting treatment and to reduce the impact of **confounding**. […] Incidence rates for each outcome were calculated based on the numbers with the outcome and the person years of follow-up, and were age and sex standardised for each	"**Confounding** factors. It is possible that patients at higher risk of […] may preferentially be prescribed […] rather than […], so all analyses were **adjusted for** demographic and clinical variables, either because	"In patients with […], […] was associated with a lower risk of […] than […] (adjusted hazard ratio 0.66, 95% confidence interval 0.54 to 0.79). […]Table 5 shows the **number needed to treat** or	"Although many adjustments have been done using the data available on the existing databases, there is a possibility of **unmeasured confounding** or **confounding** by indication. […] Although we used a proportional	The aim is to assess the safety of a drug and they use different strategies to adjust for confounding, including propensity scores, provide NNT and discuss the

For peer review only

	compared with […].”	drug. To estimate the risks associated with each […], an outcome specific Cox model containing all **confounding** factors was used, with […] as a primary reference.”	they may have been used as indicators for prescribing a specific […] or because they have possible associations with increased risk of […]. We similarly adjusted for comorbidities, previous events, and drugs also used as indicators or associated with increased risks.”	**number needed to harm** to measure the relative benefits or risks of […] in comparison with […].”	hazard model adjusting for all available **confounding** factors, we also undertook a sensitivity analysis using the **propensity score method** and obtained very similar results.”	unmeasured confounding assumption.
“To examine the **association** between […] and the **risk of** developing […].”	“Our study indicates that adherence to a […] is **associated with a substantially reduced risk of** […]. These	“To evaluate the **association** between […] and […], we calculated relative risks and 95% confidence intervals using multivariable log-binomial regression models with generalized estimating	“We first evaluated associations with […] by categories of each low risk factor, **adjusting** for […].”	“In multivariable analyses (model 2), […] had a **relative risk** of 3.10 (95% confidence interval 2.69 to 3.57) of […],	“Several factors could contribute to the weak **mediation effect** of […] in the association between […] and […] risk. […]	They provide adjusted estimates, discuss mediation and residual confounding and suggest to

For peer review only

	findings highlight the **potential benefits of implementing** […] interventions to curb the risk of […].”	equations and specified an exchangeable correlation structure.”		compared with […].”	Another limitation, as in any observational study, is that we cannot exclude the possibility of **uncontrolled confounding** by […] or **residual confounding**. […] Our findings highlight the potentially critical role of […] in the **etiology** of […] and lend support to […] based **intervention strategies** for reducing […].”	take action given the findings.
“To determine **rates of** […] and all cause mortality in patients with […] compared to	“Patients with […] remain at higher risk of […] than patients without […].	“We carried out two retrospective cohort studies to determine incidence rates of […] (primary outcome) and all cause mortality (secondary	“Poisson regression was used to calculate **adjusted** incidence rate ratios, adjusting	“The crude incidence rate ratio was 0.73 (95% confidence interval 0.65 to 0.81, P<0.001).	“In light of the evidence produced by this study, it is recommended that clinical	Even though the use of causal language is not explicit, they compare rates

For peer review only

patients with [...] and without [...].”	The **risk** is increased even in those in whom [...] is not documented. **Guidelines** should be updated to advocate continued use of [...] in patients with [...]”	outcome)in patients with [...] versus randomly selected **matched controls** with [...]. We calculated crude and **adjusted** incidence rate ratios comparing the incidence of [...]”	for the baseline covariates [...]”	Adjusting for potential confounders [...] made little difference to the incidence rate ratio: 0.76 (95% confidence interval 0.67 to 0.85, P<0.001)”	guidelines and schemes designed to incentivise appropriate management [...] are updated”	of the condition in the different groups that have been matched, provide adjusted estimates and suggest to update guidelines to reflect the findings.
“To examine the **association** between [...] at [...] and [...].”	“[...] during the period [...] iis **safe** with respect to the risk of [...].”	“We estimated odds ratios of [...] and [...] and associated Wald type two sided 95% confidence intervals by logistic regression. For [...] and [...], we calculated hazard ratios and associated Wald type two sided 95% confidence intervals from Cox regression models, which allow for detailed	“[...] to adjust for potential **confounding** due to temporal trends, we included [...]. [...] are well established risk factors for [...]. [...] All estimates were calculated by models with	“In analyses without covariate adjustment (model 1), [...] was **associated** with an increased risk of [...] (hazard ratio 1.69 (95% confidence interval 1.18 to 2.41)) and [...] (2.14 (1.39 to	“[...] is **not causally related** to increased risks (...). Instead, our results suggest other factors underlying and confounding the associations between [...]. Furthermore, although our	The abstract suggests a causal aim when describing the intention to establish a temporal relationship between the exposure and the outcome

For peer review only

		adjustment for censoring affecting the length of follow-up of each child. Days since birth was used as the underlying time scale. Each child was followed from birth until a diagnosis of the outcome, death, or end of follow-up at 31 December 2014, whichever event occurred first. ”	increasing complexity, beginning with models without adjustment for covariates (model 1), followed by models adjusting for all included potentially **confounding** covariates (model 2).”	3.30); fig 2). After covariate **adjustment** (model 2), [...] was only associated with an increased risk of [...] (adjusted hazard ratio 1.66 (1.06 to 2.59); fig 2).”	results suggest that [...] are not causally associated [...] could be a causal factor for other outcomes. [...] although the present study did not find a causal link [...], replication of the results is imperative.”	and concluding that the exposure is safe. The main text uses causal language explicitly when describing the strategies to control for confounding and concluding that a causal relationship was discarded.
“To assess the association of [...] and risk factors for [...] with [...] at [...].”	“The **independent association** between [...] and [...] in [...] is comparable in strength and consistency with those for [...]. The results	“We used a generalised additive mixed model (GAMM) to estimate [...], with [...] as fixed effect predictors and [...] as random effect at the intercept and [...] slope. [...] We computed 95% confidence intervals from the uncertainty of the	“We considered [...] as potential confounders.”		“As our analyses relied on cross sectional data, these findings should be interpreted cautiously and should not be considered as causal estimates	The abstract suggests a causal aim when describing the intention to establish a temporal relationship between the

For peer review only

	of this study suggest that **tackling** all these risk factors might substantially increase life years spent in good physical functioning.”	estimated smoothing function. We computed the **number of years of functioning lost** from the mixed model predictions”			of the impact of [...] on [...]. [...] Given that the present study is based on observational data, our study informs about associations but cannot provide evidence of causality.”	exposure and the outcome and concluding that the link is comparable to those for other established risk factors. It is important to note that In the abstract the design of the study is described as “Multi-cohort population based study”. However the method and discussion refer to a “cross sectional” design that limits the possibility to establish causal links.

"To determine outcomes and **safety** of [...] for [...], due to [...], in routine clinical practice."	"In routine clinical practice, [...] for patients with [...] is at least as **effective** and **safe** as in the setting of a randomised controlled trial."	"We used regression models to compare baseline characteristics and outcomes in patients [..] with those in the [...] intervention and control arms. The effect of [...] on [...] at 90 days in patients [...] compared with [...]l was expressed as an **adjusted** common odds ratio, derived from multivariable ordinal logistic regression (shift analysis)."	"We **adjusted** for [...]"	"After **adjustment** for [...], the shift towards [...] was significant for patients [...] compared with those [...] intervention arm (adjusted common odds ratio 1.30, 95% confidence interval 1.02 to 1.67; P=0.03) and control arm (1.85, 1.64 to 2.34; P<0.01; fig 1). "	"The results of our study might have important implications for the future of [...] for [...]. [...] is at least as **effective** and **safe** as in the setting of a randomised controlled trial."	The abstract and main text point to a causal aim as the intention is to assess the safety of an exposure in relation to an outcome and the conclusion is that not only is safe but also effective.
"To investigate whether [...] is **associated** with an **increased risk** of [...]."	"In a **propensity score matched** cohort, [...] use was associated with an increased risk of [...]. This	"Cox proportional hazards regression, with days since start of treatment as the time scale, was used to estimate the hazard ratio for [...], comparing	"We used two major strategies to control for **confounding**. To account for potential **confounding** by	"There was an increased risk of [...] associated with [...] (hazard ratio 1.66; 95% confidence interval 1.12 to	"An important concern in any observational study is the possibility of confounding. We used an active	Both the abstract and main text use causal language and causal methods

	association appeared to be largely **driven by** [...].".	episodes of [...] and [...] use."	indication [...], we used an **active comparator design**, [...] To control for potential **confounding** from differences in baseline health status, we used a **propensity score matched** design, taking into account demographic characteristics, medical history, [..]."	2.46). This increase corresponded to an absolute difference of 82 (95% confidence interval 15 to 181) cases of [...] per 1 million treatment episodes in the 60 day risk period."	comparator to limit confounding by factors associated with [...], including confounding by indication, and propensity score matching derived from a range of covariates. Despite this, the possibility of residual confounding (for example, due to [...]) cannot completely be ruled out."	including propensity score matching. They discuss the possibility of residual confounding mainly because of the observational nature of the study but also suggest possible confounders missed.
"To examine the **risks** of [...] in patients with [...] and in a general population comparison cohort."	"[...] was associated with increased risks of [...]. [...] may be an important risk factor for [...]."	"We did a population based **matched cohort** study based on routinely and prospectively collected data. [...] We calculated the 0-1 year, >1-5 years, and >5-19 years	"Using the full hospital history (inpatient and outpatient diagnoses) recorded in the DNPR before the	"After adjustment for the covariables, [...] was associated with [...] (adjusted hazard ratio 1.49,	"Although we and adjusted the analyses for a wide range of potential **confounders** identified a priori	Both the abstract and main text describe the exposure-outcome relation in a

For peer review only

		cumulative incidence per 1000 people for each outcome, accounting for the competing risk of death. Correspondingly, we used matching factors stratified (conditional) Cox proportional hazards regression to estimate hazard ratios, adjusting for the categorical comorbidities listed above as covariables."	index date, we obtained information on the following [...] risk factors: [...]"	95% confidence interval 1.36 to 1.64), [...] (2.26, 2.11 to 2.41), and [...] (1.94, 1.68 to 2.23), as well as [...] (1.59, 1.45 to 1.74) and [...] (1.25, 1.16 to 1.36) (fig 2). We found no association with [...] (adjusted hazard ratio 1.12, 0.96 to 1.30) or [...] (1.04, 0.93 to 1.16).	on the basis of excisting literature, we cannot exclude influence of unknown or **residual confounding**, for example, by [...]"<br><br>** Typos copied as in the published version	matched cohort. They discuss the possibility of residual confounding and suggest possible confounders missed. All of these elements point to a causal aim.
"To determine if [...] is a **critical determinant** of [...] and [...]."	"[...] does not have a **clinically important association** with [...] or [...]."	"We assessed the effect of [...] compared with [...], using multivariable regression. Modified Park's tests were used to determine the appropriate regression models (gamma, Poisson, and logistic) for discrete [...]	"In all of our primary analyses we adjusted for the following key **confounders**: [...]"	"Table 2 shows that there was no strong evidence of a **clinically important association** of [...] and [...] with [...] or [...]."	"We recognise that we assessed multiple associations and the isolated positive association of [...] and [...] may reflect a chance	Both abstract and main text use causal language and explain that the aim is to identify whether the exposure is a

For peer review only

		outcomes. We also assessed the effect of [...] on an individual's repeat [...] outcomes scores. [...] Linear mixed effects models were fitted with time as a fixed effect and a random effect of subject."			finding, particularly as there was no consistent association with [...] at any other [...]. [...] We would suggest the overall impact would be potentially small as there was no **clinically important impact** on [...] at any age. We acknowledge that [...] may have attenuated to the null any potential detrimental effect of [...] on [...] outcomes, but this would further support that [...] **does not have** permanent consequences for [...]."	cause of the outcome and after adjusting for potential confounders conclude that it is not, given that they only identify one positive association when multiple were assessed and consider it to be by chance.

For peer review only

"To determine if [...] is **associated** with an increased risk of [...] in the general population."	"[...] is **associated** with a **greater risk** of [...] compared with [...], but not a greater risk of death. The relative risk increase is similar across population groups, but the **higher baseline risk** among those [...] **translates into higher absolute risks of** [...] in these groups."	"We calculated odds ratios for each outcome [...] within 14 days of [...] comparing each [...] adjusting for potential **confounders** using logistic regression."	"Based on a priori knowledge, we considered the following variables as potential **confounders** of the relation between [...] and [...]: [...]. All covariates other than sex and ethnicity were updated over time. [...] We initially adjusted for sex and age only, and then fitted an adjusted model using [...]."	"In the 14 days after [...], [...] is associated with the highest odds of [...] (adjusted odds ratio 1.72, 95% confidence interval 1.31 to 2.24) and [...] (2.27, 1.49 to 3.45) of all the [...] investigated. [...] The odds of death within 14 days of [...] were similar to [...] for [...] (0.90, 0.76 to 1.07) and the other [...]."	"We saw minimal differences in the odds ratios for [...]. [...] analyses using multivariable regression and inverse probability treatment weighting approaches [...] were consistent. [...] our study also had greater ability to adjust for detailed characteristics, such as [...], which are likely to have reduced **residual confounding**. "	The elements that point to a causal aim: confounder adjustment by regression models, sensitivity analysis using inverse probability of treatment weighting and discussing residual confounding.
"To evaluate the [...] **safety** of [...],	"In this large cohort study,	"For each comparison and for all outcomes, we	"We considered the following	"Table 3 shows that after	"Randomized controlled trials	Both abstract and main text

in direct comparisons with [...], as used in routine practice.”	[...] was associated with a **lower risk** of [...]l and with a **similar risk** of [...] in direct comparisons with [...] as used in routine care.”	calculated unadjusted and **propensity score matched** number of events, incidence rates, and hazard ratios with 95% confidence intervals.”	covariates as potential **confounders**: [...] To control for imbalances in patient characteristics between cohorts, we calculated exposure **propensity scores** as the predicted probability of receiving the treatment of interest (ie, [...] v each comparator) conditional upon the subjects’ baseline covariates using three separate multivariable logistic regression models. All variables were included and no	**propensity score matching**, for [...] primary outcome, the number of events for [...] and the [...] comparator were 91 and 124 respectively (8.9 v 12.8 per 1000 person years; hazard ratio 0.70, 95% confidence interval 0.54 to 0.92) in cohort 1; 94 and 148 (7.5 v 12.4; 0.61, 0.47 to 0.78) in cohort 2; and 77 and 154 (7.3 v 14.4; 0.51, 0.38 to 0.67) in cohort 3.”	are the best way to assess drug efficacy [...] On the other hand, strict inclusion and exclusion criteria and rigorous safety monitoring limit the **generalizability** of randomized controlled trial results. Our study [...] allowing better generalizability to routine care [...] provides data from direct comparisons. [...] while we used **propensity score matching** to balance more than 100 baseline characteristics between the groups, **residual**	use causal language and they are explicit to state that the aim is to evaluate safety of the exposure and use causal methods (propensity score matching). They discuss why the design was observational and consider that due to this nature, residual confounding cannot be excluded that may have led to downtown of conclusion which is phrased more

For peer review only

			further selection was conducted. We 1:1 **matched** cohorts on their **propensity score** using a caliper width equal to 0.2 of the standard deviation of the logit of the propensity score."		**confounding** by some unmeasured characteristic(s) cannot be ruled out. "	in terms of association.
INCONSISTENT						
"To evaluate the relation between […] and development of […]"	" […] was associated with an increased risk of […] that was mediated by […]. Systematically addressing […] may be an important public health strategy to	"We calculated the hazard ratios for the relation of […] to the risk of MRSA using Cox proportional hazard models. […] We also calculated the absolute risk difference. […] We performed mediation analyses to examine the extent to which the effect of […] on the risk of […] was through	"We performed a matched cohort study […]matched on age (one year either way), sex, and study entry time (within one year either way). Such comparators were chosen to	"The matched and multivariable adjusted hazard ratios for patients with […] were 1.69 (1.51 to 1.90) for […] and 1.26 (1.12 to 1.40) for […]."	"Our GP practice based dataset could have missed the detection of some inpatient cases of […]; however, these potential non-differential misclassifications would have	

For peer review only

	reduce the incidence of […] among patients wih […]."	[…]. Using marginal structural models we then estimated the natural direct effect […] and the natural indirect effect […] while adjusting for the same confounding variables"	further ensure the comparability […] In the multivariable Cox model we adjusted for […]."		biased our results towards the null, rendering our findings conservative"	
"To quantify the effects of varying […]"	"[…] is associated with a large increase in […] among […] patients. The data from this study suggest that […] rather than […] is more strongly associated with […]"	"For adjusted analysis of time until […] we used Cox proportional hazards models."	"Adjusted models included […]"	"Each additional […] increased the rate of […] by 70.7% (95% confidence interval 54.6% to 88.4%) before adjustment and increased the hazard of […] by 44.0% (40.8% to 47.2%, P<0.001) after adjusting for covariates."	"To determine the extent to which strong unobserved confounding might explain the observed association, we included this synthetic confounder in a Cox model. […] As part of a sensitivity analysis, we constructed models that removed potential	

					confounders. […]”	
“To evaluate the long term association between […]”	“Widespread utilisation of […] may be contributing to long term increased risk of […]. The potential for […] should be considered when[…].”	“We conducted Poisson regression analyses using person years as observations.”	“We included several variables as known confounders or effect modifiers in the relation between. […]The final fully adjusted model adjusted for […]”	“After adjustment for covariates, the rate ratio was […] indicating that during the entire period of follow-up the risk of […] was 21% higher during […] than at other times.”	“The registered active […] population is generally representative of the UK population in terms of age, sex, and regional distribution”	
“To investigate the association of […]”	“The shape of the association between […] and […] was determined by […].This finding suggests that the […] may be largely explained by […]”	“We used Cox proportional hazards models to estimate hazard ratios and 95% confidence intervals. We stratified the analysis by age in months and calendar year of the questionnaire cycle.”	“For the main analysis, we used […] measured at baseline to minimize the effect of underlying diseases on mortality […] In multivariable models, we adjusted for potential	“A multivariable adjusted model showed a positive association between […] and all cause mortality, whereas […] showed a U shaped association with all cause mortality. In a	“Our findings remained robust in several sensitivity analyses […] we cannot entirely rule out the possibility of unmeasured or unknown confounding factors that may account for the	

For peer review only

			confounders including […]”	mutually adjusted model including both […] and […] we consistently observed a strong positive association between […] and all cause mortality.”	associations observed in this study.”	
“ To examine the associations of […]”	“This association could be explained by the finding that […] These results emphasise the importance of revisiting […] or establishing specific guidelines for management among […]”	“We performed Cox models with penalised splines”	“In final Cox models with penalised splines, we made adjustments for: […]”	“[A]fter adjustment for confounding factors, the U shaped association with […]”		

" To estimate long term survival, health, and educational/social functioning in patients with […]"	"[…] had no substantial effect on […]"	"We calculated mortality rate ratios and incidence rate ratios as measures of relative risk."	"For each […] patient, we used the Danish Civil Registration System and the DNPR to identify all Danish residents with the same sex and date of birth as the patient who had not tested positive […] and who met the study's inclusion and exclusion criteria […]. From this population, we extracted 10 people at random for each patient. People in the population comparison cohort were assigned the same date of study inclusion as	Patients and members of the comparison cohort were well matched with respect to […] Mortality was not higher among patients in the […] cohort"		

For peer review only

			[…] patients to whom they were matched.”			
“To compare the risk of […]”	“ Although residual confounding cannot be excluded, this finding deserves consideration when […] is used for […]”	“We estimated the crude hazard ratio of […] using Cox proportional hazard regression, and the adjusted hazard ratio was obtained using propensity score matching”	“ We identified potential confounders that were plausibly associated with both […]based on clinical knowledge […] In the context of this study, the propensity score is the probability of receiving […] as opposed to […], given the baseline characteristics. Patients who received […] were matched to patients who […] using a 1:1 nearest neighbor matching algorithm with a	“The crude hazard ratio of death in the unmatched cohort was 1.51 (95% confidence interval 1.22 to 1.85) and the adjusted hazard ratio in the matched cohort was 1.50 (1.14 to 1.96)”	“Comparison of the baseline characteristics in the unmatched cohort provided little evidence of confounding […] it is unlikely that a few additional unmeasured variables can explain a 50% increase in the risk independent of all other confounder and proxies of confounders that are adjusted for in our study.”	

For peer review only

			caliper of 0.2 of the standard deviation of the propensity score on the logit scale. Covariate balance between the two groups was assessed after matching, and we considered an absolute standardized difference less than 0.1 as evidence of balance”			
“To determine whether […] is associated with […]”	“[…] was independently associated With […]”	“We fitted both a mixed effect logistic regression model (in which the outcome was defined as dichotomous […] and the Prentice, Williams, and Peterson (PWP) model”	“We examined the relation between […] and […] adjusted for …”	“The rate of distinct criteria met per year increased by 24% if a patient had been admitted to hospital (hazard ratio 1.24, 95% confidence interval 1.20 to 1.28) when	“We did a sensitivity analysis using propensity score matching to assess whether the association between […] and […] could be due to unmeasured confounders […]	

For peer review only

				controlled for the other covariates"	Although we adjusted for a range of characteristics of patients, as with any observational study potential exists for unmeasured confounding, which may partly or fully explain the relation between […]"	
"To investigate associations between […]and to analyse the effect of changes […]"	"Risks of […] are inversely associated with […]"	"We used multivariable Cox regression analysis to compare the rates of […] and […]."	"Confounders included in the final models were based on the literature or statistical significance (P<0.10). The full model included […]"	"Compared with […], […] had increased hazard ratios of […]"	"We believe that our findings are widely applicable and provide justification for […] and continuing […]"	

For peer review only

"To estimate the rates of […]"	"In cases of […], approximately […] will become […], of which a third will have […]."	" We present denominators where data for the secondary outcome are missing. We defined the population attributable fraction as (Re–Run)/Re=(RR–1)/RR, calculated using Stata. To test the robustness of our findings, we did a sensitivity analysis."	"We compared the demographic and clinical variables of […]. We used the binomial Wilson score to calculate confidence intervals of single proportions and the Pearson exact method to calculate confidence intervals of risk ratios and medians."	"[…] had a higher risk of […]The population attributable fraction of […] was 47% for […] and 61% for […]"	" Considering these results when counselling potentially exposed […] seems reasonable"	
To perform an expedited assessment of […] risk associated with exposure to […]".	"The results do not imply a markedly increased short term overall risk of […] in […]."	"We used Cox regression to estimate the hazard ratio with 95% confidence intervals for […] associated with […], both for ever use and for the predefined categories of cumulative use"	"Analyses were, however, performed as crude comparisons adjusted only for […] as well as adjusted for […] and the potential	"Overall, exposure to […] showed no association with […] compared with exposure to […] (adjusted hazard ratio 1.09, 95% confidence interval 0.85 to	"This ensured that the estimates were not affected by immortal time bias [..] As all comparisons were performed within users of […], the exposure	

For peer review only

			confounding factors.”	1.41) and no evidence of a dose-response relation”	to […] can reasonably be expected to be a random event, and confounding is thus expected to be limited.”	
“To investigate the risks of […] in […]”	“No increased risk of […] was detected in […], but increased risks of […] were found in this study. Our results suggest that […] risks could be due to […], rather than […].”	“To calculate expected […], we multiplied the person years at risk by corresponding national incidence rates (by 5 year age band and individual calendar year) for the general female population of England and Wales. Standardised incidence ratios were calculated by the comparison of observed values with expected values.”	“We obtained data relating to potential confounding factors such as […]”	“There was no overall increased risk of […] (2578 observed v 2641.2 expected […]; standardised incidence ratio 0.98 (95% confidence interval 0.94 to 1.01); absolute excess risk −2.8 cases per 100 000 person years (95% confidence interval −7.1 to 1.8); table 2)”.	“Given previous inconsistent results, small study size, and lack of information on potential confounders, we undertook a population based linkage study in […]”	
CONSISTENTLY NOT CAUSAL						

For peer review only

"To determine whether [...], compared [...], is **associated** with an increased risk of [...]"	"In this population based cohort study, [...] was **associated** with an increased risk of [...]. The association was particularly elevated among people using [...] for more than five years. **Additional studies**, with long term follow-up, are needed to investigate the **effects** of [...] on [...]."	"We calculated crude incidence rates of [...] and 95% confidence intervals, based on the Poisson distribution, for each exposure group. We used time dependent Cox proportional hazards models to estimate hazard ratios and 95% confidence intervals of [...] associated with [...] compared with [...], using multiple imputation for variables with missing values."	"Potential **confounders**. All models were **adjusted** for the following variables measured at cohort entry: [...]. [...] as an alternate means of controlling for **confounding**, we repeated the analysis by stratifying the model on tenths of disease risk score. Finally, we repeated the analysis using a **marginal structural** Cox proportional hazards model using **inverse probability of treatment** and censoring	"Compared with [...], [...] were **associated** with an overall 14% **greater risk of** [..] (1.6 v 1.2 per 1000 person years; hazard ratio 1.14, 95% confidence interval 1.01 to 1.29)."	"We introduced a one year exposure lag period to account for a minimum latency time window and to minimize **reverse causality**. [...]The association between [...] and [...] is **biologically plausible**. [...] although we were able to adjust for several important **confounders**, this study lacked information on other potential **confounders** such as [...] In this large, population based study, [...] was associated with an elevated risk of [...] overall, along with	In the abstract they only describe associations but in the full text their interest points to a causal aim given the different methods applied to adjust for confounding and reverse causality. They also consider elements as biologically plausibility and duration response relation. Residual confounding seems to be a concern because they

			weighting—a method designed to adjust for time dependent **confounding** associated with time varying exposures”		evidence of a **duration-response relation.**”	lacked information on relevant cofounders which could lead to down tone of the conclusions.
“To determine whether […] and […] are **associated** with an **increased risk of** […] in adults with […].”	“Compared with […], […], and […], **might be associated** with an increased risk of […] in adults with […].”	“For each exposure category we calculated crude incidence rates of […] with 95% confidence intervals, based on the Poisson distribution. Time dependent Cox proportional hazards models were used to estimate hazard ratios and 95% confidence intervals of […] associated with […] and […], separately, when compared with […].”	“The models were **adjusted** for the potential **confounders** measured at cohort entry: […] To minimise potential **confounding** by indication, we compared […]. […] we fit a **marginal structural model** to investigate the impact of potential time dependent	“Compared with […], […] was **associated** with a 77% increase in the hazards of […] (hazard ratio 1.77, 95% confidence interval 1.04 to 3.01).”	“Finally, we excluded those with less than one year of follow-up after cohort entry, to allow for a sufficient latency period and to minimise **reverse causality**. […] To assess possible **duration-response relations**, we investigated the association between	The abstract only refers to association but the full text mentions adjusting for confounders and ways to minimise reverse causality. They also describe its biological plausibility. There is a concern for residual confounding

For peer review only

			**confounding** using **inverse probability of treatment** and censoring weighting."		cumulative duration of […] on the risk of […]. An association between […] and incidence of […] is **biologically plausible.** […] as with all observational studies, **residual confounding** is possible. We conducted several sensitivity and ancillary analyses specifically designed to assess the potential impact of **residual** confounding."	due to the observational nature rather than missing information on particular relevant confounders.
"To examine the **association**	"[…] **could increase the risk of** […].	"[…] was categorised by percentiles (<10, 10-20, 20-50, 50-80, 80-90, ≥90).	"Characteristics that might influence the risk	"[…] was significantly associated with	"[…] the mechanism that might be	The abstract only considers associations

For peer review only

between […] and […] risk of […].”	However, confirmation of these findings are warranted, preferably in an intervention setting”	[…] With these same categories of exposure, the association between […] and […] was examined by Cox regression. We used […] age from birth up to May 2016 as the underlying timescale censoring if death or emigration from Denmark occurred (1217 events).”	of [….] were identified a priori and included as potential **confounders** in our **adjusted** analysis. In model 1, we adjusted for: […].In model 2, additional adjustments were made for […]”	**increased risk of** […] in both unadjusted and covariate **adjusted** analyses (table 3). Compared with […], offspring of those with […] had double the risk of […] during follow-up (hazard ratio 2.00 (95% confidence interval 1.02 to 4.00)). Risk of […] was positively **associated** with […]: the **association** was significant (Ptrend=0.016) and increased monotonically. Only minor differences were observed between the unadjusted and	responsible for this **effect** is not known, but could include […]. […] the role of unmeasured or unidentified confounders can never be fully excluded in observational studies.”	but the full text mentions confounder adjustments and discusses potential mechanism (biological plausibility). Concern of residual confounding is due to the observational nature of the study.

For peer review only

				covariate **adjusted** analyses.”		
“To ascertain compliance **rates** with […]; to identify features **associated** with non-compliance; to **rank** […] by compliance; and to build a tool for live ongoing audit of compliance.”	“Compliance with […] has been poor, with half of all […] non-compliant. […] commonly contain inconsistencies that might prevent even […] assessing compliance. Accessible and timely information on the compliance status of […] and […] may help to improve reporting **rates**.”	“We constructed a logistic regression model with all these **explanatory** variables, as they were selected prospectively on the basis of clinical and methodological interest.”	“**Explanatory** variables. We created variables for a range of features of each […], selected prospectively on the basis of clinical and methodological interest.”	“In the **adjusted** multivariable analysis, […] with a […] were **significantly more likely to** […] (adjusted odds ratio 23.3, 95% confidence interval 19.2 to 28.2); as were […] (18.4, 15.3 to 22.1).”	--	Although adjusted estimates are present, both abstract and full text limit to describes associations, rates and ranks. No causal language is used.

"To assess **how often** older adults […] were […], and to identify markers of […]."	"**One in seven** older adults […] were […]. **More than half of** […] occurred in patients with […]. More attention is needed to reduce potentially harmful […] as older adults […]."	"We did multivariable mixed effect logistic regression analyses to determine **associations** between the outcome of […] and primary **predictors** of […]."	"Our primary **predictor** variables were […]. **Adjusted** analyses included the covariates noted above, a random effect term to account for clustering by hospital, and an interaction term to account for the relation between […] and […]."	"A total of 2074 (14%) patients were […]; 1293 (9%) were […] and 300 (2%) were […]. Additionally, 628 (4%) patients were […]. […] Patients with […] had a 25% (95% confidence interval 23% to 78%) probability of […]."	--	The abstract indicates that the aim is to assess the frequency of a condition and that is reflected in the main text. No causal language used.
"To describe **trends** in the **rate** and daily dose of […] used among […] from 2007 to 2016."	"[…] **rates** were high during the study period of 2007-16, with the **highest rates** in […] versus […] and […]. […] and average daily dose have not	"Endpoints were defined at the person quarter level. We used logistic regression to model the **proportion** of the population […] each quarter. The average […] per person day by quarter was modelled by a generalized linear model with negative binomial	"All analyses were stratified by beneficiary category including commercially insured, aged Medicare, and disabled Medicare	"Averaged across the entire study period, 51.5% of disabled Medicare beneficiaries […] per year (n=1 128 088), compared with 14.3% (n=18 721 915) of	--	The abstract indicates that the aim is to describe the frequency of a condition which is reflected in the main text. No

	**substantially declined** from their peaks, despite increased attention to […] and awareness of their **risks**."	family and log link. The dependent variable was the total […] per person in the quarter, with an exposure variable representing the number of days of insurance coverage for each person included to **standardize** daily […]."	(beneficiaries with Medicare coverage who were under age 65 years)"	commercial beneficiaries and 25.7% (n=3 847 676) of aged Medicare beneficiaries."		causal language used.
"To **describe** […] related mortality in the United States during 1999-2016 by age group, sex, race, cause of […], and geographic region."	"Mortality due to […] has been **increasing** in the US since 2009. Driven by deaths due to […], people aged 25-34 have experienced the **greatest relative increase** in mortality. White Americans,	"Our primary aim was to describe temporal trends in death rates attributable to […] and […] as the primary or underlying cause of death for adults in the USA.[…] We then evaluated trends in death rates using the National Cancer Institute's Joinpoint program. This enabled us to identify if there were years in the study period where the rate of change in mortality was statistically	"We adjusted rates for age—that is, age specific mortality was weighted according to the age distribution in a standard year (2000). We also sought to describe how these trends differed based on demographic subgroups; age, sex, race (Asian or	"During the study period, a total of 460 760 deaths were attributed to […] (20 661 in 1999 and 34 174 in 2016) and 136 442 to […] (5112 in 1999 and 11 073 in 2016) (table 1). Men had a higher **burden** of age **adjusted** mortality due to […] compared	"[…] though we have detected worsening mortality since 2009, the precise reasons for this trend and the geographic heterogeneity in our analysis require further study. For example, we identify the states most at risk, but granular data are	The abstract indicates that the aim is to describe the frequency of a condition which is reflected in the main text. No causal language used.

	Native Americans, and Hispanic Americans experienced the **greatest increase** in deaths from […]. Mortality due to […] is improving in Maryland but worst in Kentucky, New Mexico, and Arkansas. The rapid increase in death rates among young people due to […] highlight new challenges for optimal care of patients with […].”	significantly different. The program uses a piecewise linear regression approach to determine whether rates over time are best described by a straight line (0 joinpoints) or by multiple linear segments (≥1 joinpoints)”	Pacific Islander, Native American (designated as “American Indian” in the census database) or Alaska Native, black or African American, and white American), Hispanic ethnicity, and geographic area of residence.”	with women by a 2:1 ratio and a higher **burden** of mortality due to […] by a nearly 4:1 ratio.”	needed to determine the root causes”	

For peer review only

"To examine whether [...] are **associated** with an increased risk of [...] after [...]."	"Women with [...], especially [...], may be at **higher risk of** [...]. If these findings are replicated elsewhere, a massive amount of data exists that could aid in identifying women at **higher risk of** [...] and that could be conveyed to them or their healthcare providers."	"The main model assessed the primary [...] composite outcome, as well as the individual outcomes of [...], in relation to each [...] for the screened cohort, with censoring at a woman's death or arrival at the end of the study period of 31 March 2016, allowing for a maximum follow-up of 22 years. We did time to event analyses using multivariable Cox regression models,to derive a hazard ratio and 95% confidence interval for each study outcome."	"Hazard ratios were **adjusted** for variables chosen a priori, based on the existing literature, including: [...]"	"A total of 6209 women developed the primary [...] composite outcome, which was typically about 1.2 to 1.3 times **more likely** to occur in a [...], even after **adjustment** for other covariates"	"Potential **confounders** between [...] and the risk of [...], including [...] were each accounted for in the models. Nevertheless, about 10% of [...] lacked information on [...], and [...] and [...] were entirely unknown."	The abstract indicates that the aim is to identify the association between an exposure and an outcome. They consider residual confounding due to lack of information on relevant confounders.
"To examine the **association** between [...] and the risk of [...]	"Among [...], increasingly worse [...] was **associated** with	"Using generalised linear models with a robust sandwich estimator, we estimated risk ratios for	"Analyses were **adjusted** for [...]"	"In analyses based on [...] levels, the **adjusted** risk	"It is also possible that the previously demonstrated	The abstract indicates that the aim is to identify the

according to levels of [...].”	a **progressively increased** risk of [...]. Even with [...] within target levels recommended by guidelines [...], the risk of [...] was increased more than twofold. The risk of [...] was not statistically significantly increased at any of the [...] levels examined; the study had limited statistical power for this outcome and was based on [...] only.”	[...], comparing [...] according to levels of [...] with [...]. To take into account possible dependence from repeated [...], we constructed models with [...] as a cluster variable. [...] were assumed to follow a poisson distribution, and we estimated risk ratios using a log link function”		ratios for [...] were 2.17 (95% confidence interval 1.37 to 3.42) for [...], 3.17 (2.45 to 4.11) for [...], 2.79 (1.90 to 4.12) for [...], and 6.23 (4.32 to 9.00) for [...] versus [...]. The corresponding **adjusted** risk differences were 17 (95% confidence interval 5 to 36), 32 (21 to 46), 26 (13 to 46), and 77 (49 to 118) cases per 1000 [...].”	**association** [...] has resulted in increased clinical surveillance for defects among [...]. [...] health registers do not record data on [...] and hence we could not account for these factors.”	association between an exposure and an outcome. They consider residual confounding due to lack of information on relevant confounders.

"To examine the **association** between risk factor burdens—categorized as optimal, borderline, or elevated—and the lifetime risk of [...]."	"Regardless of index ages at 55, 65, or 75 years, an optimal **risk factor** profile was associated with a lifetime risk of [...] of about one in five; this risk rose to more than one in three in individuals with at least one elevated risk factor."	"We calculated the lifetime risks for the first incident [...] from index ages 55, 65, and 75 yearsup to age 95 years. [...] we used a modified Kaplan-Meier estimator with age as the time scale, accounting for the competing risk of death to compute the lifetime cumulative risk of [...] and associated 95% confidence intervals"	"We computed lifetime **risk in subgroups** of participants according to their risk profile at a specified index age (optimal, borderline, and elevated), for each **risk factor** separately and for the combination of **risk factors**. [...] we fitted a multivariable Fine and Gray model, **adjusted** for competing risk of death to **predict** the lifetime risk of [...]."	"The associated lifetime risk of [...] was lowest if the risk factor profile was optimal. The lifetime risk of [...] increased gradually as the risk factor profile changed from optimal to borderline and elevated at each index age."	"Our observational study design limits the ability to establish **causal pathways**, and only **associations** between **risk factor** profiles and lifetime risk of [...] can be concluded from our study."	The abstract and main text indicate that the study mainly aims at identifying associations. There is a concern for residual confounding due to the observational nature rather than missing information on particular relevant confounders.

For peer review only

"To compare **rates** of [...] for patients [...], with patients [...]."	"[...] was **associated** with lower [...] **rates** compared with [...]."	"We used a Cox proportional hazards regression model, adjusting for (...), to estimate hazard ratios and 95% confidence intervals for [...] comparing [...] with [...]. To summarize switchback estimates across [...], we conducted inverse variance weighted random effects meta-analyses"	"[...] **adjusting** for basic demographics (age, sex, and calendar year)"	"Figure 5 shows that in the adjusted analysis, the [...] rates remained consistently lower among [...] than [...]. The magnitude of this effect was largest for [...] (hazard ratio 0.52, 95% confidence interval 0.43 to 0.63) and smallest for [...] (0.86, 0.77 to 0.97). The pooled hazard ratio across [...] suggested that [...] was associated with a 28% lower rate of [...] compared with b[...] (0.72, 0.64 to 0.81)."	"[...] our results indicate that [...] may in part be **driven by** [...]."	Even though causal language is not used and both the abstract and main text mainly describe associations, their conclusion at the end of the text suggests a causal relationship.

"To assess whether [...] is **associated** with the incidence of [...] in patients with [...]."	"In this first population based study, [...] was **associated** with an increased risk of [...]. Although these findings need to be replicated, physicians should be aware of this possible association."	"We calculated crude incidence rates of [...] with 95% confidence intervals based on the Poisson distribution for the entire cohort and for each exposure group. For all analyses, we used time dependent Cox proportional hazards models to estimate hazard ratios and 95% confidence intervals for [...] associated with [...] compared with [...]. We also calculated the **number needed to harm** for patients followed over a two year and four year period by using methods accounting for varying patient follow-up times."	"The models were **adjusted for** the following potential **confounders** measured at cohort entry: [...]"	"Compared with [...], [...] was **associated** with a 75% **increase in risk** of [...] (53.4 v 34.5 per 100 000 per year; hazard ratio 1.75, 95% confidence interval 1.22 to 2.49). The **number needed to harm** corresponded to 2291 patients followed over a two year period and 1177 over a four year period."	"[...] as with all observational studies, **residual confounding** from unknown or unmeasured variables remains possible. However, on the basis of the rule out method, a hypothetical confounder would need to be strongly associated with both the exposure (odds ratio >4.7) and the outcome (relative risk >5.0) to move the point estimate towards the null."	The abstract mainly describes associations but in the main text they describe how their estimates were adjusted for potential confounders and they estimate numbers needed to harm. Also they consider residual confounding and suggest that only a strong unmeasured confounder will remove the association observed. All of these, point

						to a causal analysis.
“To assess the prospective **associations** between [...] and risk of [...].	“In this large prospective study, a 10% increase in the proportion of [...] associated with a **significant increase** of greater than10% in risks of [...]. Further studies are needed to better understand the relative effect of the various dimensions of [...] in these associations.”	“We used Cox proportional hazards models with age as the primary timescale to evaluate the association between [...] and incidence of [...]. In these models. We estimated hazard ratios and 95% confidence intervals with the lowest quarter as the reference category.”	“Models were **adjusted** for [...] we made additional adjustments [...]. In addition we did **mediation** analyses [...]”	“In model 1, [..]. was associated with increased risks of overall cancer (hazard ratio for a 10 point increment in the proportion of [...] 1.12 (95% confidence interval 1.06 to 1.18), P<0.001) and [...] (1.11 (1.02 to 1.22), P=0.02). .”	“Lastly, although we included a large range of **confounding factors** in the analyses, the hypothesis of **residual confounding** resulting from unmeasured factors (...) cannot be entirely excluded owing to the observational design of this study”	The abstract mainly describes associations but in the main text they describe how their estimates were adjusted for potential confounders and they use mediation analysis which suggest a causal aim. They also consider residual confounding due to the observational nature of the study.

"To assess the **association** between [...] and all cause mortality in [...] with [...]."	"Giving [...] to [...] with [...] was **associated** with an increased rate of [...] but a paradoxical lowered rate of all cause mortality. Careful consideration should be given before [...]. There remains an urgent need for adequately powered randomised trials in this population to explore these findings and to provide clarity on correct clinical management."	"We calculated the incidence of [...] and all cause mortality per 100 person years of follow-up. We generated Kaplan-Meier survival curves for the outcomes of interest grouped by [...] status. Cox proportion regression were reported as **adjusted** hazard ratios with 95% confidence intervals. Because all baseline characteristics were balanced in the **propensity matched** model, [...] was the only independent variable in our Cox regression model."	"We used propensity score matching with demographic and clinical variables to adjust for potential confounding from imbalances in clinical characteristics between patients receiving and not receiving [...]. Demographic variables were [...]. Clinical variables were [...]. Patients who received anticoagulation were matched in a 1:1 ratio"	"The crude rates for [...] and [...] were 4.6 and 1.2 after [...], and 1.5 and 0.4 in patients who [....] per 100 person years, respectively. In the Cox proportion regression models, the hazard ratios for [...], [...] and all cause mortality for patients [...] were 2.60 (95% confidence interval 2.00 to 3.38), 2.42 (1.44 to 4.05), and 0.82 (0.74 to 0.91) compared with those who [...]."	"The study population was derived from real world evidence with the inherent limitations of diagnostic coding and case ascertainment Despite well **matched** groups after **propensity score matching**, we cannot exclude that the reported associations were **confounded** by indication"	The abstract limits to describe associations and rates of the condition but the main text suggest a causal aim as they use propensity score matching to control for confounding.

"To assess the **temporal change** in [...] and to identify [...] with the greatest increase in use."	"Total [...] use has increased markedly over time, in both sexes, and across all age groups [...]. Of the patients who [...], the **proportion** who had [...] increased significantly over time."	"For comparison across years, we **standardised** crude rates to the mid-2015 UK population. Rates were calculated for [...]. Joinpoint regression was used to model the temporal changes in age and sex **standardised** rates"	"For each year we calculated the total number [...], **stratified** by age and sex. We calculated total person years of observation in each age and sex stratum for each year."	"The age and sex **adjusted** rate of [...] increased from 14 869 tests per 10 000 person years in 2000/1 to 49 267 in 2015/16 (table 1, fig 1), an annual increase of 8.5% (95% confidence interval 7.6% to 9.3%). [...] The slope of the trend line changed significantly at two points: 2004/5 (P<0.001) and 2008/9 (P=0.004) (fig 1)."	"We analysed the temporal change in [...] from UK primary care. The total use of tests increased markedly over time, even after adjustment for population growth"	The abstract and full text state that they aim to quantify changes of a condition over time. It is a descriptive study that does not use causal language accordingly.
To investigate the **association** of [...] with disease specific incidence and mortality and	"[...] was **associated** with a range of health outcomes and	"[...] we investigated the associations of [...] with cause specific incidence and mortality over follow-up with Cox proportional	"We treated [...] as potential **confounders**. For Cox proportional hazard analyses,	"As shown in figure 1, in both men and women, [...] was **associated** with a	"To minimise the potential contribution of **reverse causality** to the findings,	The abstract mentions associations and suggests that the aim is

For peer review only - http://bmjopen.bmj.com/site/about/guidelines.xhtml

whether [...] enhances the **prediction** ability of an established office based **risk score**.""	improved **prediction** of an office based **risk score**. Further work on the use of [...] in risk scores or risk screening is needed to establish its potential clinical utility."	hazard models. We reported the results as hazard ratios together with 95% confidence intervals."	we ran four models that included an increasing number of covariates: model 0 (minimally adjusted) included [...]"	higher hazard for all cause mortality and incidence of and mortality from [...] in model 0. The **associations** were similar after **adjustment** for [...] in model 1; after further adjustment, the magnitude of associations were slightly attenuated in models 2, 3, and 4"	we did a landmark analysis excluding events occurring within the two years after recruitment in model 4 (landmark analysis). This landmark analysis was adjusted as in model 3. [...] may, therefore, be a useful method of **identifying** people with [...] who are at high risk of a wide range of diseases. [...] **Reverse causality** is possible in any observational study. [...] Similarly, **residual confounding** is always possible and the	prediction. In the main text, the authors conclude that the exposure of interest enhances prediction and identification of patients with risk of certain diseases. However, there is use of causal language, including confounder adjustment and discussing reverse causality and residual confounding. They note that their goal is to do prediction and that

For peer review only

					associations observed may not imply **causality**. However, given that we are largely interested in **prediction** and identification of people at increased risk, and not seeking to make strong causal inferences, **reverse causality** is not a major limitation. ”	reverse e causality is not a major limitation but still adjust for it.
To **externally validate** four commonly used rules in [...] for [...].“”	“Application of the [...] rules can lead to a wide variation in [...] among patients with [...], **resulting** in many unnecessary [...] findings.	“The sensitivity, specificity, and proportion of patients [...] (with 95% confidence intervals) were assessed for each of the four decision rules. [...] The Cochran’s Q test was used to directly compare the sensitivities and specificities between the	--	“The sensitivity for identifying patients with [...] ranged from 72.5% for the [...] criteria to 98.8% for the [...] rule (table 4; appendix 3). [...] The [...] criteria would	--	Both the abstract and the full text state that they aim to validate four decision rules for a particular condition. They

For peer review only

	Until an existing decision rule has been updated, any of the four rules can be used for patients presenting [...] at the emergency department. Use of the [...] rule is recommended because it **leads to** a substantial reduction in [..] while missing few potential [...].”	four decision rules [...]. Net proportional benefit has been proposed to incorporate such weighting in calculation of clinical usefulness of decision rules. For each rule, we expressed the net proportional benefit using theformula: (true positives/total number) − weight × (false positives/total number). ”		have missed 11 of 74 patients with [...] (appendix 4). The CHIP criteria would have missed two patients with [...], who both had [...]. The specificity for identifying [...] was lowest for the [...] rule (4.4%) and highest for the [...] criteria (60.9%). [...] The sensitivity and specificity differed significantly between all the rules (Cochran’s Q P<0.001). ”		compared the tests in terms of sensibility or specificity and concluded that the tests are similar and recommended the use of a particular one given that it can help avoid false negatives. The wording ’resulting’ and ‘leads to’ are in fact to discuss the potential for false positives/ false negatives rather than a casual claim.
“To develop and **validate** a set of	“The **prediction** models reliably	“The **association** between predictor variables and [...]	“In a published systematic	“Bootstrap resampling	--	The abstract and main text

practical **prediction tools** that reliably estimate the outcome of [...].”	estimate the outcome of patients who were managed in various settings for [...]. The **predictor** items are readily derived at hospital admission. The web based [...] **prognostic** calculator [...] and the related app could be adjunctive tools to support management of patients.”	was analysed by fitting proportional odds logistic regression models **adjusting** for the fixed effect of study. Prognostic strength was quantified as odds ratios with 95% confidence intervals. The relative importance of each **predictor** in the models was estimated with partial R2 statistic, which estimates the independent **contribution** of the **predictor** to the variance of the outcome”	review, we identified relevant **predictors** of outcome in patients [...]. Based on the results of this published review, we selected the following **predictor** variables that are assessable early at hospital admission and are consistently associated with outcomes for inclusion in the **prediction** models: [...]”	showed negligible model optimism. The models had **internally validated AUCs** between 0.77 and 0.83. There was no significant lack of fit (goodness of fit P≥0.2 in all models). Cross validated performance was variable across studies [...]. The partial R2 values ranged between 4% and 46%, and the pooled AUC values were between 0.74 and 0.77”		state that the goal of the study is to validate a tool. Consistent with the prediction aim, no causal language is used.
“To prospectively **validate** [...] to triage patients	“In a population of patients referred for	“To examine model performance, we constructed a logistic	--	“The triaging strategy [...] predicted true	--	The abstract and main text describe that

For peer review only

with [...] in routine clinical practice.”	[...], this new triaging approach accurately **classified** [...] for most, with half the utilisation of ABPM compared with usual care. This triaging strategy can therefore be recommended for diagnosis or management of [...] in patients where [...] is being considered, particularly in settings with limited resources.”	regression model with true [...] as the dependant outcome variable and classification using [...] as the independent predictor variable. From this model we estimated the **area under the receiver operating characteristic (AUROC) curve** statistic.”		[...] (true positives 66%, 95% confidence interval 63% to 69%; true negatives 24%, 22% to 27%) with a low error rate (false positives 8%, 6% to 10%; false negatives 2%, 1% to 3%) (table 2). The triaging strategy resulted in 49% (46% to 52%) being referred for [...] and the remainder managed on the basis of their clinic measurements.”		the aim is to validate a triage tool and assessed its performance compared to the standard of reference. As the aim is prediction, no causal language is used accordingly.

For peer review only

For peer review only

For peer review only - http://bmjopen.bmj.com/site/about/guidelines.xhtml
